# Design of Diarylheptanoid Derivatives as Dual Inhibitors Against Class IIa Histone Deacetylase and β-amyloid Aggregation

**DOI:** 10.3389/fphar.2018.00708

**Published:** 2018-07-03

**Authors:** Liang-Chieh Chen, Hui-Ju Tseng, Chang-Yi Liu, Yun-Yi Huang, Cheng-Chung Yen, Jing-Ru Weng, Yeh-Lin Lu, Wen-Chi Hou, Tony E. Lin, I-Horng Pan, Kuo-Kuei Huang, Wei-Jan Huang, Kai-Cheng Hsu

**Affiliations:** ^1^Graduate Institute of Pharmacognosy, College of Pharmacy, Taipei Medical University, Taipei, Taiwan; ^2^School of Pharmacy, College of Pharmacy, Taipei Medical University, Taipei, Taiwan; ^3^Ph.D. Program in Biotechnology Research and Development, College of Pharmacy, Taipei Medical University, Taipei, Taiwan; ^4^Graduate Institute of Cancer Biology and Drug Discovery, College of Medical Science and Technology, Taipei Medical University, Taipei, Taiwan; ^5^Department of Marine Technology and Resources, College of Marine Sciences, National Sun Yat-sen University, Kaohsiung, Taiwan; ^6^Herbal Medicinal Product Division, Industrial Technology Research Institute, Hsinchu, Taiwan; ^7^Ph.D. Program for the Clinical Drug Discovery from Botanical Herbs, College of Pharmacy, Taipei Medical University, Taipei, Taiwan; ^8^School of Pharmacy, National Defense Medical Center, Taipei, Taiwan

**Keywords:** Alzheimer’s disease, Aβ aggregation, histone deacetylase, isoform-selective inhibitors, dual inhibitors

## Abstract

Alzheimer’s disease (AD) is a progressive neurodegenerative disorder with multiple etiologies. Beta-amyloid (Aβ) self-aggregation and overexpression of class IIa histone deacetylases (HDACs) are strongly implicated with AD pathogenesis. In this study, a series of novel diarylheptanoid derivatives were designed, synthesized and evaluated for use as dual Aβ self-aggregation and class IIa HDAC inhibitors. Among these compounds, **4j, 5c**, and **5e** displayed effective inhibitions for Aβ self-aggregation, HDAC5 activity and HDAC7 activity with IC_50_ values of <10 μM. The compounds contain three common features: (1) a catechol or pyrogallol moiety, (2) a carbonyl linker and (3) an aromatic ring that can function as an HDAC cap and create hydrophobic interactions with Aβ_1-42_. Furthermore, compounds **4j, 5c**, and **5e** showed no significant cytotoxicity to human neuroblastoma SH-SY5Y cells and also exhibited neuroprotective effect against H_2_O_2_-induced toxicity. Overall, these promising *in vitro* data highlighted compounds **4j, 5c**, and **5e** as lead compounds that are worthy for further investigation.

## Introduction

Alzheimer’s disease (AD) is a progressive neurodegenerative disorder that occurs more frequently in the elderly and is one of the most common causes of dementia. The pathology of AD is multifactorial and not yet fully understood. Current hypotheses include: extracellular deposition of beta-amyloid (Aβ), formation of intracellular neurofibrillary tangles by hyperphosphorylated tau protein, acetylcholine deficiency, glutamate excitotoxicity, neuroinflammation and widespread neuronal loss (Ahn et al., 2017; Hung and Fu, 2017). Due to the multiple pathogenic mechanisms in AD, a single target drug may not ameliorate the disease. Therefore, multi-target drugs may be a more prudent choice for developing AD therapeutics (Leon et al., 2013).

The amyloid hypothesis is generally believed to be responsible for the formation of senile plaques in the brain of patients with AD (Hardy and Selkoe, 2002; Bu et al., 2016). Beta-amyloid plaques are formed when amyloid precursor protein (APP) is cleaved by β- and γ-secretase to produce Aβ monomers with 38 to 42 amino acids (Hardy and Selkoe, 2002; Kuperstein et al., 2010; Bu et al., 2016). Among them, Aβ_1-40_ is the most abundant. However, Aβ_1-42_ has lower solubility than Aβ_1-40_ and thus, is more susceptible to self-aggregation, which can lead to different degrees of neurotoxicity (Gilbert, 2013; Bu et al., 2016). Studies have shown that increasing the Aβ_1-42_: Aβ_1-40_ ratio can stabilize toxic Aβ oligomers (Kuperstein et al., 2010). Therefore, the inhibition of Aβ self-aggregation is a potential strategy for prevention or treatment of AD.

The class IIa isozymes HDAC5 and 7 are closely associated with AD (Koseki et al., 2012; Anderson et al., 2015). Additionally, class IIa HDACs are highly expressed in the brain regions associated with learning and memory and distributed in specific tissues, such as the brain, lung or heart (Bolden et al., 2006; Volmar and Wahlestedt, 2015). HDAC5 is increased in the frontal cortex of AD patients compared to control patients (Anderson et al., 2015). Overexpression of HDAC7 decreased β-catenin activity (Margariti et al., 2010). Downregulation of Wnt/β-catenin signaling has been implicated AD progression (Fischer et al., 2010; Vallee and Lecarpentier, 2016). Furthermore, *in vitro* studies using trichostatin A (TSA), a histone deacetylase (HDAC) inhibitor, resulted in a significant up-regulation of Aβ degrading enzyme neprilysin (NEP) expression in SH-SY5Y cells (Kerridge et al., 2014). These studies suggest that the inhibition of class IIa HDAC activity is a promising strategy for the treatment of AD.

**FIGURE 1:**
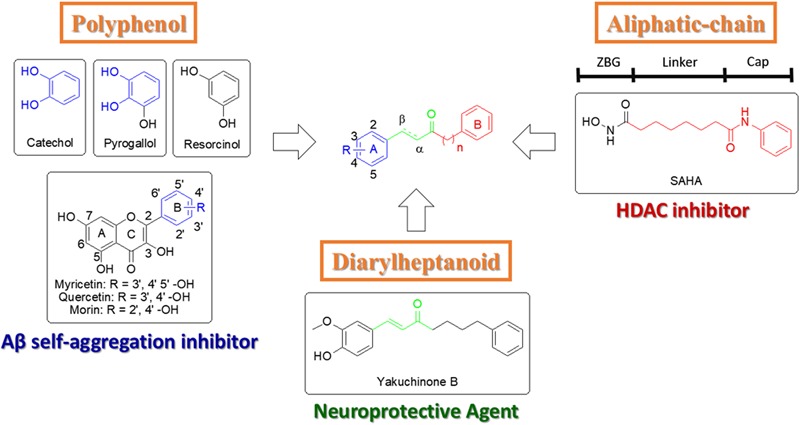
Design strategy for new catechol-modified diarylheptanoid derivatives.

Due to the complex etiology of AD, developing a multi-target drug may present significant clinical efficacy (Leon et al., 2013). This strategy can avoid disadvantages seen in single target drugs that only offer a palliative treatment (Leon et al., 2013). In recent years, many studies have focused on combining at least two pharmacophoric moieties into one molecule to increase efficacy of drugs (De Simone et al., 2017; Taguchi et al., 2017; Xu et al., 2018). Flavonoids (e.g., morin, myricetin, and quercetin) are polyphenolic compounds that contain a catechol or pyrogallol scaffold and have shown inhibition of Aβ self-aggregation (**Figure 1**, blue section) (Bu et al., 2016). The FDA approved HDAC inhibitor suberoylanilide hydroxamic acid (SAHA) contains an aliphatic-chain linker and a hydrophobic benzene cap (**Figure 1**, red section) (Miller et al., 2003). Furthermore, another compound with neuroprotective effect is yakuchinone B, which contains an α, β-unsaturated carbonyl of yakuchinone B extends a π-electron system in conjugation with the phenyl ring (**Figure 1**, green section) (Jang et al., 2009; Lee et al., 2014). Thus, these structures, which are potential therapies for AD, can be exploited to create a dual Aβ and HDAC inhibitor.

In this study, we designed and synthesized a series of diarylheptanoid derivatives as dual Aβ self-aggregation and class IIa HDAC inhibitors. The inhibitory potency of the compounds was evaluated for Aβ_1-42_ self-aggregation and HDAC activity. Then, the compounds were virtually docked to analyze their structure-activity relationship (SAR). Furthermore, *in vitro* cytotoxicity and neuroprotective effects of the synthesized compounds were investigated. The compounds in this study may have the potential for use in AD treatments.

## Materials and Methods

### Materials

The NMR spectra (^1^H and ^13^C NMR) were obtained with a Bruker Fourier 300 and AVIII 500 using standard pulse programs, and chemical shifts are cited in parts per million (ppm, δ) downfield from TMS as an internal standard. NMR data were processed using Bruker TOPSPIN software. The MS data was measured on Finnigan Mat TSQ-7000 mass spectrometer (HRESIMS) (Thermo, Ringoes, NJ, United States). The HPLC was performed on a C18 column (150 mm × 4.6 mm, Ascentis) by an L-2130 pump (Hitachi, Ibaraki, Japan) and a UV/vis L-2420 detector (Hitachi, Ibaraki, Japan). The column chromatography was performed on silica gel (70–230 mesh, Merck, Darmstadt, Germany). All TLC analyses were performed on silica gel plates (KG60-F254, Merck). The microplate spectrophotometer was used for fluorometric analysis by Victor 2X (Perkin Elmer, Fremont, CA, United States) and absorbance analysis by Sunrise microplate reader (TECAN, Männedorf, Switzerland) or Synergy HT (Bio-Tek, Winooski, VT, United States). The microplate incubator shaker was used for ThT assay by BioShake iQ ThermoMixer (Quantifoil Instruments GmbH, Jena, Germany). Unless otherwise mentioned, reagents and materials were used without further purification and purchased from ACROS (Geel, Belgium). 3, 4-Dihydroxybenzaldehyde and 2, 3-dihydroxybenzaldehyde was purchased from Alfa Aesar (Haverhill, MA, United States). 3-Fluoro-4-hydroxybenzaldehyde, 3-chloro-4-hydroxybenzaldehyde, 3-bromo-4-hydroxybenzaldehyde and 1-(triphenylphosphoranylidene)propan-2-one was purchased from AK Scientific (Union City, CA, United States). Tributyl(1-ethoxyvinyl)tin was purchased from Combi-Blocks (San Diego, CA, United States). 10% Pd-C was purchased from Sigma–Aldrich (Saint Louis, MO, United States). Dry dichloromethane (DCM) was distilled from CaH_2_ under nitrogen atmosphere. All reactions were conducted under an atmosphere of dry N_2_.

### Chemistry

#### General Procedure for the Synthesis of Compounds 2 and 11 by Wittig Olefination

To a solution of 1-(triphenylphosphoranylidene)propan-2-one (1.2 equiv) in dry DCM was added compound **1** or **10** (1 equiv) dropwise by syringe, respectively. The resulting mixture was heated to reflux and stirred for 12 h. The reaction mixture was concentrated in vacuo, diluted with EtOAc (100 mL) and washed with distilled H_2_O (3 × 50 mL). The organic layer was dried over Na_2_SO_4_, filtered and concentrated in vacuo. The residue was purified by silica gel chromatography (EtOAc: *n*-hexane = 1:9) to give α, β-unsaturated ketones **2** (5.1 g, 79%) and **11** (213 mg, 87%).

#### General Procedure for the Synthesis of Compounds 3, 5a–i, 6d, and 8a–d by Hydrogenation

A catalytic amount of 10% Pd-C was added to a solution of unsaturated compounds in EtOH and the mixture was stirred at RT under H_2_ atmosphere for 5 h. The reaction mixture was filtered with celite and the filtrate was concentrated in vacuo. The residue was purified by silica gel chromatography (EtOAc: *n*-hexane = 1:11) to give α, β-saturated ketones **3** (5.1 g, 99%), **5a** (35 mg, 97%), **5b** (52 mg, 95%), **5c** (51 mg, 99%), **5d** (70 mg, 77%), **5e** (79 mg, 78%), **5f** (55 mg, 61%), **5g** (37 mg, 37%), **5h** (39 mg, 39%), **5i** (96 mg, 99%), **6d** (187 mg, 95%), **8a** (30 mg, 60%), **8b** (21 mg, 52%), **8c** (33 mg, 66%) and **8d** (27 mg, 67%).

#### General Procedure for the Synthesis of Compounds 4a–k and 7a–d by Aldol Condensation

To a solution of compound **3** (1.5 equiv) in THF was added pyrrolidine (1.5 equiv), acetic acid (1.5 equiv) and benzaldehyde (1 equiv). The mixture was heated to reflux for 6 h. The reaction mixture was diluted with EtOAc (100 mL) and washed with distilled H_2_O (3 × 50 mL). The organic layer was dried over Na_2_SO_4_, filtered and concentrated in vacuo. The residue was purified by silica gel chromatography (EtOAc: *n*-hexane = 1:15) to give diarylheptanoid **4a** (226 mg, 73%), **4b** (156 mg, 89%), **4c** (94 mg, 50%), **4d** (54 mg, 34%), **4e** (225 mg, 67%), **4f** (125 mg, 74%), **4g** (61 mg, 34%), **4h** (110 mg, 54%), **4i** (109 mg, 59%), **4j** (85 mg, 48%), **4k** (95 mg, 62%), **7a** (145 mg, 73%), **7b** (51 mg, 29%), **7c** (130 mg, 72%), and **7d** (104 mg, 72%).

#### Synthesis of Compound 6b by Stille Coupling Reaction

To a mixture of compound **9** (1 equiv) and PdCl_2_(PPh_3_)_2_ (0.1 equiv) in toluene (5 mL) was added tributyl(1-ethoxyvinyl)tin (1.1 equiv). The resulting solution was heated to 80°C and stirred for 20 h. After cooling to RT, the reaction mixture was acidified with 1 M HCl _(aq)_ and stirred for 3 h. The mixture was diluted with EtOAc (30 mL) and washed with distilled H_2_O (3 × 30 mL). The organic layer was dried over Na_2_SO_4_, filtered and concentrated in vacuo. The residue was purified by silica gel chromatography (EtOAc: n-hexane = 1:9) to give compound **6b** (421 mg, 79%). The experimental details and physical data of all synthetic compounds can be found in the Supplementary Materials.

### Pharmacological Assay

#### Inhibition of Aβ_1-42_ Self-Aggregation

Inhibition of Aβ_1-42_ self-aggregation was measured by thioflavin-T (ThT) fluorescence assay (Hudson et al., 2009). Aβ_1-42_ peptides were purchased from Kelowna International Scientific Inc. To avoid Aβ_1-42_ self-aggregation before the start of the experiment, Aβ_1-42_ peptides were dissolved in hexafluoroisopropanol (HFIP) to a concentration of 1 mM. The clear solution containing the dissolved peptide was then aliquoted in micro centrifuge tube. The solution was evaporated under vacuum to remove HFIP to give monomeric Aβ_1-42_. The monomeric Aβ_1-42_ was dissolved in NaOH_(aq)_ (60 mM) to give a stock solution (1 mM). The Aβ_1-42_ stock solution was diluted with 50 mM phosphate buffer (containing 0.15 M NaCl, pH 7.4) before use. Fresh ThT stock solution was prepared by dissolving ThT (Sigma, T3516) in phosphate buffer and filtrated through 0.45 μM Nylon syringe filter. The ThT stock solution and 400 mM ethylenediaminetetraacetic acid (EDTA) were diluted into the phosphate buffer to generate the working solution as background. A mixture of working solution (ThT final concentration: 10 μM), Aβ_1-42_ stock solution (Aβ_1-42_ final concentration: 10 μM) and the tested compounds were incubated on BioShake iQ ThermoMixer (37°C, 700 rpm) for 24 h. Fluorescence was measured by Victor 2X microplate spectrophotometer (excitation λ = 440 nm, emission λ = 486 nm), and adapted for 96-well microtiter plates. The fluorescence intensities were recorded and the percent inhibition due to the presence of the inhibitors were calculated by the following formula: (1 - IF_i_/IF_c_) × 100% where IFi and IF_c_ were the fluorescence intensities obtained for Aβ_1-42_ in the presence and in the absence of inhibitors after subtracting the background, respectively. The IC_50_ values were calculated by the linear regression model of the data. All experiments were performed in triplicate.

#### Preparation of HDAC4

Preparation of HDAC4 was performed as previously described (Huang et al., 2012). Genes encoding HDAC4 (residues 648–1057) flanked with NdeI and EcoRI sites at the 5′- and 3′-ends, were synthesized by GenScript Corporation (Piscataway, NJ, United States) and subcloned into expression vectors pET-28a(ϸ) and pET-24b(ϸ). Proteins were expressed in BL21(DE3) cells by overnight induction with IPTG (1 mM) at 20–25°C and purified from cleared cell lysates by sequential chromatography on Ni-Sepharose 6 fast flow, Mono Q 5/50 GL, and Superdex 75 10/300 GL columns (GE Healthcare). Protein concentrations were quantified with Bradford Reagent (Bio-Rad).

#### Inhibition of HDACs

Inhibition of HDACs were measured by the HDAC fluorometric activity assays (Wegener et al., 2003). Enzyme, inhibitors and substrate were diluted with HDAC buffer (15 mM Tris-HCl pH 8.1, 0.25 mM EDTA, 250 mM NaCl, 10% v:v glycerol). Briefly, 10 μL diluted HDAC, i.e., HeLa nuclear extract (Enzo), HDAC6 (BPS), HDAC4, HDAC5 (BPS), HDAC7 (BPS) and HDAC9 (BPS), as well as 50 μL of testing compounds at different concentrations were added to each well of a 96-well microtiter plate and pre-incubated at 30°C for 5 min. The enzymatic reaction was started by addition 40 μL substrate, including Boc-Lys(Ac)-AMC (Bachem) 10 μM for HeLa nuclear extract and HDAC6, and Boc-Lys(TFA)-AMC (Bachem) 10 μM for HDAC4, fluorogenic HDAC class IIa substrate, Catalog #: 50040 (BPS Bioscience, San Diego, CA, United States) 10 μM for HDAC5, HDAC7 and HDAC9 into the HDAC buffer. After incubation at 37°C for 30 min, the reaction was stopped by adding 100 μL trypsin solution (10 mg/mL trypsin in 50 mM Tris-HCl pH 8, 100 mM NaCl, 2 mM SAHA). After incubation at 37°C for another 20 min, fluorescence was measured (excitation λ = 355 nm, emission λ = 460 nm) with VICTOR X2 microplate spectrophotometer. The fluorescence in wells without test compounds (0.1% DMSO, negative control) was set as 100% enzymatic activity and the fluorescence in wells with enzyme eliminated was set as 0% enzymatic activity. The fluorescence ratio of test compounds to negative control was defined as percentage of remaining enzyme activity. The IC_50_ values were calculated by linear regression of the data. All experiments were performed in triplicate.

#### Molecular Modeling Analysis

The computational analysis was executed using Discovery Studio (DS) (BIOVIA et al., 2016). The CDOCKER program in DS was used for molecular docking of Aβ_1-42_ and HDAC7. Structures for Aβ_1-42_ (PDB ID: 1Z0Q) and HDAC7 (PDB ID: 3ZNS) were obtained from Protein Data Bank (PDB). The structures were then prepared by the automatic ligand preparation function in DS Macromolecules Tools for protonation, removal of water molecules and mismatched residues fixing. The protonated state was set to 7.4 pH. The Aβ_1-42_ binding site was detected automatically with a 10 Å radius sphere by DS. The HDAC7 binding site was defined by the co-crystallized ligand within a 10 Å radius sphere. The compounds were prepared by the ligand preparation function in DS Small Molecules Tools for protonation and fixing bad valence. The compounds were then docked to the binding sites by the Docking Optimization function of CDOCKER using the following parameters: random conformations of each ligand were set to 30, refined orientations for each conformation was set to 30 and a value of maximum bad orientations was set to 1600. The simulated annealing for each orientation was set for heating to 700 K over 2,000 steps followed by cooling to 300 K over 5,000 steps. The CHARMm forcefield and Momany-Rone ligand partial charge method were employed for the scoring function to generate the docking score called -CDOCKER interaction energy. In total, the program generated 10 top-ranked poses for each compound and generated an interaction profile of all poses using the DS interaction analysis function. Final binding poses of each compound in Aβ_1-42_ and HDAC7 were selected according to their -CDOCKER interaction energy. All binding poses have a -CDOCKER interaction energy score of at least 30 for HDAC7 and at least 20 for Aβ_1-42_. Total energy was generated for each pose using the CHARMm forcefield in DS with the following parameters: solvent model set to none, non-bond list radius set to 14.0 Å and electrostatics set to automatic. We identified a maximum common substructure (MCSS) of all compounds. This was used to create a root-mean-square deviation (RMSD) analysis. The absorption, distribution, metabolism, excretion and toxicity (ADMET) was calculated using the ADMET component in DS. Each category is separated into different levels: blood brain barrier (BBB) penetration levels 0 (very high) to 4 (undefined), human intestinal absorption levels 1 (very low solubility) to 5 (very soluble) and absorption level 0 (good solubility) to 3 (very low solubility). The ADMET component used default parameters.

#### Cell Culture

SH-SY5Y cells were kindly provided by Professor Zhi-Hong Wen (National Sun Yat-sen University) and cultured in DMEM/F12 (Gibco, Grand Island, NY, United States) supplemented with 10% heat-inactivated fetal bovine serum and penicillin (100 U/ml)/streptomycin (100 μg/ml) (Invitrogen, Carlsbad, CA, United States) at 37°C in an atmosphere of 5% CO_2_.

#### Cytotoxicity Effect on SH-SY5Y Cells

Cell viability was assessed using the alamar blue (7-hydroxy-10-oxidophenoxazin-10-ium-3-one) cell viability assay in 6 replicates as described previously (Feng et al., 2016). Briefly, 5 × 10^3^ cells/well were seeded in 96-well flat-bottomed plates; 24 h later, cells were treated with compounds or DMSO as control. After 24 h of incubation, the medium was removed, 100 μL medium containing 10 μL of alamar blue was added, and cells were incubated in the 5% CO_2_ incubator at 37°C for 4 h. Absorbance at 570 nm was determined by Synergy HT microplate reader. The results were averaged over three different independent experiments. The cell viability was also evaluated by trypan blue exclusion test.

#### Neuroprotection Against H_2_O_2_-Induced Neurotoxicity on SH-SY5Y Cells

H_2_O_2_-induced neurotoxicity on SH-SY5Y cells was used to evaluate the neuroprotective effect (Rafatian et al., 2012; Omar et al., 2017). Briefly, a density of 2 × 10^4^ SH-SY5Y cells were seeded per well in 96-well plates. After 24 h of incubation, the medium was replaced with serum free medium containing H_2_O_2_ at a concentration of 500 μM and treated with different concentrations of compounds (40, 20, and 10 μM). SH-SY5Y cells were also cultured without any treatment as control group and results were indicated by percentage of control. After 3 h, cell viability was performed by MTT assay. The cells were treated with MTT (5 mg/ml in PBS) for 4 h at 37°C. The formazan crystals were generated by viable mitochondrial succinate dehydrogenase from MTT. The supernatant was then aspirated off and the formazan crystals were dissolved with DMSO. Absorbance at 570 nm was determined by Tecan Sunrise microplate reader. The trypan blue exclusion test was also used to evaluate the neuroprotection.

## Results and Discussions

### Chemistry

**Scheme 1:**
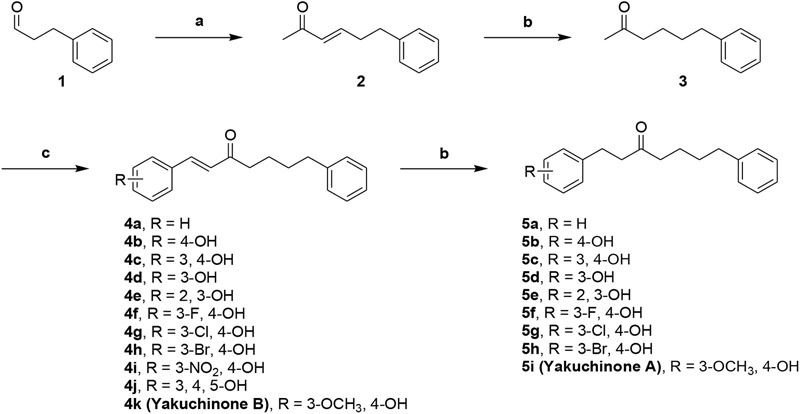
Synthetic route to compounds **4a–k** and **5a–i**. Reagents and conditions: (**a**) 1-(triphenylphosphoranylidene)propan-2-one, CH_2_Cl_2_, Δ, 79%; (**b**) 10% Pd-C, H_2_, EtOH, RT, 37-99%%; (**c**) benzaldehyde or substituted benzaldehyde, pyrrolidine, acetic acid, THF, Δ, 34–89%.

We designed dual inhibitors using the diarylheptanoid scaffold as the core structure, due to its linear structure and neuroprotective capabilities seen in previous studies (Jang et al., 2009) (**Scheme 1**). First, we synthesized various compounds with different substituted groups on the A ring. Polyphenols were used to achieve Aβ_1-42_ self-aggregation and HDAC IIa inhibition (Bu et al., 2016). Therefore, we added a number of hydroxy groups and electron-donating or electron-withdrawing groups on the A ring. Compounds **4a–4k** and **5a**–**5i** were synthesized as described in **Scheme 1**. Wittig olefination of compound **1** with 1-(triphenylphosphoranylidene)propan-2-one generated compound **2**. Compound **2** treated with the catalytic amount of 10% Pd-C to afford compound **3**. Aldol condensation of compound **3** using various substituted benzaldehyde gave desired diarylheptanoids **4a**–**4k**. Repeated hydrogenation of compounds **4a–4k** produced compounds **5a**–**5i**, respectively.

Next, to investigate the effects of carbon linker chain on the Aβ_1-42_ self-aggregation and HADC inhibition, we carried out the synthesis of compounds **7a**–**7d** and **8a**–**8d**. These compounds contain various chain lengths of the carbon linker as described in **Scheme 2**. Stille coupling of compound **9** with tributyl(1-ethoxyvinyl)tin in the presence of PdCl_2_(PPh_3_)_2_ afforded compound **6b**. Wittig olefination of compound **10** yielded compound **11**. Hydrogenation of compound **11** gave compound **6d**. Aldol condensation of varied methyl ketones **6a–6d** with 3,4-dihydroxybenzaldehyde produced corresponding α,β-unsaturated ketones **7a**–**7d**. Finally, hydrogenation of compounds **7a**–**7d** yielded compounds **8a**–**8d**, respectively. The ^1^H and ^13^C NMR spectra can be found in the Supplementary Figures S1–S61. All compounds had an estimated purity of at least 97% as determined by HPLC analysis (Supplementary Figures S62–S89).

**Scheme 2:**
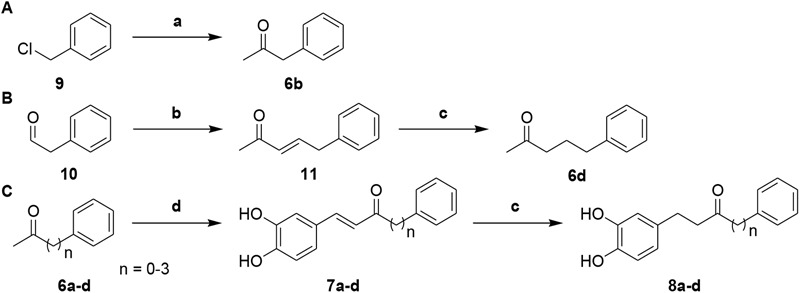
**(A)** Synthetic route to intermediate compound **6b**. **(B)** Synthetic route to intermediate compound **6d**. **(C)** Synthetic route to compounds **7a–d** and **8a–d**. Reagents and conditions: (**a**) tributyl(1-ethoxyvinyl)tin, PdCl_2_(PPh_3_)_2_, toluene, 100°C, 79%; (**b**) 1-(triphenylphosphoranylidene)propan-2-one, CH_2_Cl_2_, Δ, 87%; (**c**) 10% Pd-C, H_2_, EtOH, RT, 52-95%; (**d**) 3, 4-dihydroxybenzaldehyde, pyrrolidine, acetic acid, THF, Δ, 29-73%.

### Biological Assays and Molecular Docking Analysis

#### Inhibition of Aβ_1-42_ Self-Aggregation

The synthesized compounds were evaluated for their inhibitory potency of Aβ_1-42_ self-aggregation, using a ThT fluorescence assay (Hudson et al., 2009). First, the compounds were screened at a single concentration of 10 μM. Then, the compounds with an inhibition ratio of ≥50% were selected to determine their IC_50_ values. The inhibition ratio and IC_50_ values of the compounds were listed in **Table 1**. In general, the compounds were divided into two groups according to their chemical structures. Compounds in group one retains the diarylheptanoid scaffold and the substituents on the A ring synthesized from **Scheme 1** (compounds **4a**–**k** and **5a**–**i**). Among this group, compounds **4c, 4e, 4j, 5c**, and **5e** contain either a catechol or pyrogallol moiety. They exhibited potent inhibitions for Aβ_1-42_ self-aggregation with IC_50_ values of 6.1, 3.4, 3.6, 5.8, and 6.0 μM, respectively. Additionally, these five compounds showed better inhibitory activity than the reference compounds morin and SAHA. Interestingly, these five compounds have catechol (compounds **4c, 4e, 5c**, and **5e**) or pyrogallol (compound **4j**) groups on the A ring. These results were in line with the inhibition of Aβ_1-42_ self-aggregation by polyphenol. However, when we modified the 3-position of the A ring with an electron-donating or electron-withdrawing group (e.g., fluoro, nitro, etc.), we did not observe a significant increase in inhibitory activity. These results suggested that a catechol or pyrogallol moiety connected with α, β-unsaturated carbonyl group plays a key role for the inhibition of Aβ_1-42_ self-aggregation.

**Table 1:** Inhibitions of compounds **4a**–**k, 5a**–**i, 7a**–**d, 8a**–**d** and reference compounds against Aβ_1-42_ self-aggregation.

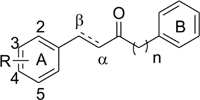					
Compound	Substitution (R)	Chain length (*n*)	α, β-saturation	Beta-Amyloid Aggregation	
				Inhibition (%) ^a,b^	IC_50_ (μM) ^b^
**4a**	H	4	Unsaturated	11.9 ± 1.0%	>10
**4b**	4-OH	4	Unsaturated	28.7 ± 6.3%	>10
**4c**	3, 4-OH	4	Unsaturated	76.2 ± 2.7	6.1 ± 1.3
**4d**	3-OH	4	Unsaturated	0.7 ± 1.5	>10
**4e**	2, 3-OH	4	Unsaturated	91.7 ± 2.5	3.4 ± 0.6
**4f**	3-F, 4-OH	4	Unsaturated	34.9 ± 9.2	>10
**4g**	3-Cl, 4-OH	4	Unsaturated	27.8 ± 7.1	>10
**4h**	3-Br, 4-OH	4	Unsaturated	28.1 ± 2.1	>10
**4i**	3-NO_2_, 4-OH	4	Unsaturated	49.0 ± 2.9	>10
**4j**	3, 4, 5-OH	4	Unsaturated	78.7 ± 6.2	3.6 ± 1.0
**4k**	3-OCH_3_, 4-OH	4	Unsaturated	23.1 ± 6.4	>10
**5a**	H	4	Saturated	14.0 ± 3.0	>10
**5b**	4-OH	4	Saturated	30.4 ± 1.0	>10
**5c**	3, 4-OH	4	Saturated	67.8 ± 3.4	5.8 ± 0.2
**5d**	3-OH	4	Saturated	-22.1 ± 2.0	>10
**5e**	2, 3-OH	4	Saturated	65.9 ± 0.2	6.0 ± 0.9
**5f**	3-F, 4-OH	4	Saturated	22.1 ± 11.1	>10
**5g**	3-Cl, 4-OH	4	Saturated	17.8 ± 2.8	>10
**5h**	3-Br, 4-OH	4	Saturated	1.0 ± 3.0	>10
**5i**	3-OCH_3_, 4-OH	4	Saturated	4.6 ± 11.7	>10
**7a**	3, 4-OH	0	Unsaturated	83.1 ± 1.9	2.3 ± 0.2
**7b**	3, 4-OH	1	Unsaturated	79.4 ± 2.6	2.1 ± 0.2
**7c**	3, 4-OH	2	Unsaturated	83.7 ± 0.8	3.4 ± 0.4
**7d**	3, 4-OH	3	Unsaturated	74.0 ± 9.5	3.1 ± 0.3
**8a**	3, 4-OH	0	Saturated	66.4 ± 1.0	8.6 ± 1.1
**8b**	3, 4-OH	1	Saturated	68.6 ± 4.1	8.1 ± 1.2
**8c**	3, 4-OH	2	Saturated	71.3 ± 3.8	8.0 ± 1.6
**8d**	3, 4-OH	3	Saturated	59.0 ± 5.8	8.5 ± 0.2
**Morin**				56.9 ± 5.9	8.8 ± 0.7
**SAHA**		–		-13.3 ± 1.8	>10

*^*a*^The thioflavin-T (ThT) fluorescence assay was used to evaluate the inhibitory activity of compounds at a concentration of 10 μM. ^*b*^Each values are presented as the mean of three independent experiments.*

The compounds of group two were synthesized to assess the effect of the linker length. Thus, compounds **7a–d** and **8a–d** retain the catechol and α, β-unsaturated carbonyl moieties, but possess shorter linkers. The most potent inhibitor was compound **7b**, which has an IC_50_ value of 2.1 μM. Furthermore, we found that α, β-unsaturated compounds significantly increased activity when compared to saturated compounds. Moreover, the compounds with the shorter linkers showed increased inhibitory activity compared to those with the longer linkers, such as compound **4c**. These experimental results indicated that compounds **4c, 4e, 4j, 5c, 5e, 7a**–**d**, and **8a**–**d** can reduce Aβ_1-42_ self-aggregation.

#### Active Compounds Have Conserved Interactions With Aβ_1-42_

**FIGURE 2:**
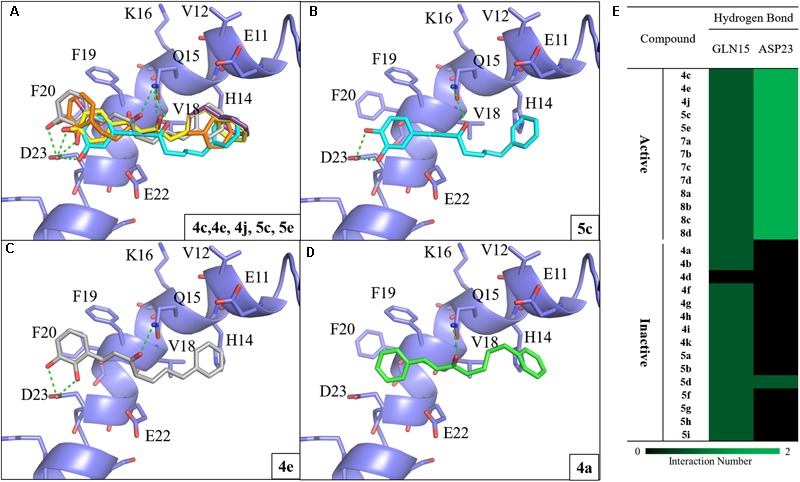
Molecular docking analysis between Aβ_1-42_ and synthesized compounds. **(A)** Docking poses of compounds **4c** (yellow), **4e** (gray), **4j** (purple), **5c** (blue) and **5e** (orange) in the binding site of Aβ_1-42_ structure (PDB ID: 1Z0Q). Docking poses of active compounds **(B) 5c (C) 4e** and **(D) 4a** with Aβ_1-42_ structure. The inactive compound **4a** (green) lacks the hydrogen bonds with residue D23. Hydrogen bonds are colored in green dash lines. **(E)** Interaction heatmap of synthesized compounds. Hydrogen bonds are colored in green.

To investigate the SAR of the designed compounds, we performed molecular docking and interaction analysis using DS (BIOVIA et al., 2016). The synthesized compounds were docked into Aβ_1-42_ (PDB ID: 1Z0Q) and contained a –CDOKER energy score ranging from 24 to 31. The total energy of protein-compound complexes can be found in Supplementary Table S1. In general, the Aβ_1-42_ active compounds have a lower total energy range. This indicates that the active compounds are more stable when bound to Aβ_1-42_. The active compounds of group one (compounds **4c, 4e, 4j, 5c**, and **5e**) produce similar interactions with the Aβ_1-42_ helix (**Figure 2A**). Compounds **4c** and **5c** contain a 3,4-dihydroxy group on the A ring, while compound **4j** contain a 3,4,5-trihydroxy group. The hydroxy groups on the A-ring 3 and 4 positions serve as hydrogen donors that form hydrogen bonds with residue D23. Simultaneously, the carbonyl group serve as a hydrogen acceptor and a hydrogen bond is formed with residue Q15. This type of binding conformation also produces favorable van der Waals (VDW) interactions between residues E11, H14, V18, F19 and E22 (**Figure 2B**). Similarly, compounds **4e** and **5e** contain a 2,3-dihydroxy group that can form hydrogen bonds with residue D23. The carbonyl group form a hydrogen bond with residue Q15. In addition, the B ring position is closer to residues V18 and H14. This forms the π-alkyl interaction between residue V18 and compound **4e**, and VDW interactions with E11, H14, F19, and E22 (**Figure 2C**). Meanwhile, the inactive compound **4a** (IC_50_ > 10 μM) does not contain hydroxy groups on the A ring and does not form interactions with D23 (**Figure 2D**). These results suggest hydrogen donors are necessary to produce two hydrogen bonds with residue D23. In summary, we identified common interactions between the active compounds. In particular, two hydrogen bonds with residue D23, and one hydrogen bonds with Q15 (**Figure 2E**).

**FIGURE 3:**
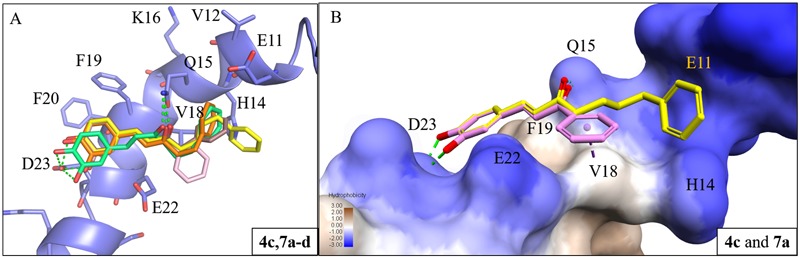
Interaction analysis between Aβ_1-42_ and active compounds **4c, 7a**-**d**. **(A)** Docking poses of compounds **4c** (yellow), **7a** (pink), **7b** (brown), **7c** (orange), and **7d** (green) in the binding site of Aβ_1-42_ structure (PDB ID: 1Z0Q). Hydrogen bonds are represented as green dash lines. **(B)** Surface model of with Aβ_1-42_ structure docked with compounds **4c** and **7a** with Aβ_1-42_ hydrophobic surface. Area with log *P* = 3 colored in brown, area with log *P* = –3 colored in blue.

To study the effect of the linker length on activity, we compared the docking poses of compound **4c** with the active compounds (compounds **7a–d**) of group two (**Figure 3A**). Like compound **4c**, compounds **7a**–**d** displayed similar polar contacts to residues Q15 and D23 (**Figure 2E**). The shorter chain length allows the B ring of group two compounds to generate π-alkyl interaction with residue V18, which stabilize the binding of the compounds. Thus, compounds **7a**–**d** displayed better activity than compound **4c** (**Figure 3B**). The results showed that our compounds with the shorter carbon-linker had the best inhibitory results for Aβ_1-42_ self-aggregation.

We also identified the MCSS of all synthetic compounds (Supplementary Figure S90) to analyze the docking poses. The RMSD value between two binding poses was calculated according to the MCSS. The RMSD matrix was generated for all of the selected binding poses and revealed two clusters. Cluster 1 contains all active compounds and two inactive compounds, while the compounds in cluster 2 are inactive. RMSD values of intra binding poses in each cluster are <2 Å (Supplementary Figure S91). The compounds in cluster 2 do not contain a catechol or pyrogallol moiety.

During the Aβ self-aggregation process, the protein secondary structure element α-helix undergoes a conformational change to β-sheet, which is linked to fibrillogenesis (Ding et al., 2003; Zhou et al., 2010). The synthesized compounds in our docking procedure form a hydrogen bond to residue Q15 as an anchor to the middle cavity of the Aβ_1-42_ structure. When comparing active and inactive compounds, hydrogen bonds with residue D23 appear to be the key for inhibitory activity. Therefore, the active compounds form interactions that disrupted Aβ_1-42_ α-helix to β-sheet conversion to prevent Aβ self-aggregation.

#### Inhibition of HDACs

Next, we looked to test the synthesized compounds HDACs inhibitory activity with a HDAC fluorogenic assay and using SAHA as the control (Wegener et al., 2003). We determined their enzyme inhibiting effects against a panel of HDAC isoforms including class IIa (HDAC4, 5, 7, and 9), class IIb (HDAC6) and HaLa nuclear HDACs, which contain class I HDACs (**Table 2**). Group one compounds (compounds **4a–k** and **5a–i**) contain a diarylheptanoid scaffold and various substituents on the A ring. We observed poor inhibitory activity on HeLa nuclear HDACs. However, group one compounds **4c, 4e, 4j, 5c**, and **5e** displayed significant inhibitions of class IIa HDACs. Furthermore, the compounds showed an increased inhibition and selectivity toward HDAC class IIa compared to SAHA. Among these, compound **5c** was the most potent, with IC_50_ values of 18.1, 3.8, and 5.3 μM for HDAC4, 5, and 9, respectively. Compound **5c** showed enhanced inhibitory activity against class IIa HDACs compared to compound **4c**, suggesting that α, β-saturated compounds had slightly increased HDAC class IIa inhibitory activity compared to unsaturated compounds. Compound **4j** was the most potent HDAC7 inhibitor with an IC_50_ value of 3.8 μM. The compounds that showed effective inhibitions of HDAC5 or HDAC7 contain a catechol or pyrogallol moiety as the A ring. This result demonstrated that catechol and pyrogallol moieties are an effective ZBG for class IIa HDACs.

**Table 2:** IC_50_ values (μM)^a^ of compounds **4a**–**k, 5a**–**i, 7a**–**d, 8a**–**d** and reference compound for HDAC inhibition.

Compound	HeLa Nuclear HDACs	Class IIa				Class IIb
		HDAC4	HDAC5	HDAC7	HDAC9	HDAC6
**4a**	>40	>40	>40	>40	>40	>40
**4b**	>40	>40	>40	>40	>40	>40
**4c**	>40	30.5 ± 0.7	15.9 ± 1.5	8.4 ± 0.3	17.6 ± 0.3	18.3 ± 0.9
**4d**	>40	>40	>40	>40	>40	>40
**4e**	>40	38.8 ± 0.1	14.6 ± 0.2	8.6 ± 1.0	18.1 ± 0.4	29.4 ± 1.5
**4f**	>40	>40	>40	>40	>40	>40
**4g**	>40	>40	>40	>40	>40	>40
**4h**	>40	>40	>40	>40	>40	>40
**4i**	>40	>40	36.1 ± 3.7	32.7 ± 2.1	>40	>40
**4j**	>40	>40	7.1 ± 0.2	3.8 ± 0.4	15.4 ± 1.8	11.9 ± 0.2
**4k**	>40	>40	>40	>40	>40	>40
**5a**	>40	>40	>40	>40	>40	>40
**5b**	>40	>40	>40	>40	>40	>40
**5c**	>40	18.1 ± 0.8	3.8 ± 0.1	4.3 ± 0.5	5.3 ± 0.3	>40
**5d**	>40	>40	>40	>40	>40	>40
**5e**	>40	35.9 ± 0.1	4.8 ± 0.4	8.7 ± 0.7	9.3 ± 0.8	>40
**5f**	>40	>40	>40	>40	>40	>40
**5g**	>40	29.9 ± 2.3	>40	30.7 ± 0.7	>40	>40
**5h**	>40	>40	>40	>40	>40	>40
**5i**	>40	>40	>40	>40	>40	>40
**7a**	>40	31.1 ± 0.8	13.1 ± 4.4	9.6 ± 0.2	18.4 ± 0.6	26.2 ± 4.3
**7b**	>40	39.8 ± 0.2	13.7 ± 0.8	15.4 ± 3.0	29.2 ± 1.7	37.2 ± 1.2
**7c**	>40	38.3 ± 0.0	15.6 ± 0.4	13.6 ± 1.1	36.8 ± 0.7	34.5 ± 2.2
**7d**	>40	35.0 ± 2.8	12.6 ± 2.3	9.4 ± 0.2	17.3 ± 0.1	37.5 ± 1.6
**8a**	>40	>40	6.2 ± 0.4	12.0 ± 1.0	14.2 ± 1.6	>40
**8b**	>40	>40	9.0 ± 0.2	13.7 ± 0.7	37.1 ± 0.9	>40
**8c**	>40	>40	8.2 ± 0.1	8.9 ± 0.4	33.8 ± 3.8	>40
**8d**	>40	>40	7.5 ± 0.3	7.4 ± 1.6	17.3 ± 3.2	>40
**SAHA**	0.02 ± 0.00	30.1 ± 4.9	12.4 ± 0.6	37.1 ± 1.3	37.7 ± 1.6	0.008 ± 0.001

*^*a*^Each values are presented as the mean of three independent experiments.*

Group two (compounds **7a–d** and **8a**–**d**) differ in the linker chain length. These compounds display selectivity toward class IIa HDAC (**Table 2**). The α, β-saturated groups on compounds **8a–d** slightly increase their potency of HDAC5 when compared to compounds **7a–d**. Nevertheless, the class IIa HDACs inhibitory activity of compounds **7a**–**d** and **8a**–**d** are inferior to that of compound **5c** (**Table 2**). In addition, compounds **7a–d** and **8a–d** show relatively poor inhibition toward HDAC7 when compared to compound **4j**. These results suggest that shortening the chain length and increasing the α, β-unsaturation does not enhance the HDAC inhibitory activity in comparison with compound **5c**. Interestingly, the compounds **4c, 4e, 4j, 5c, 5e, 7a–d**, and **8a–d** show effective Aβ_1-42_ self-aggregation inhibition as described above.

#### Molecular Docking Analysis of Compounds With HDAC7

**FIGURE 4:**
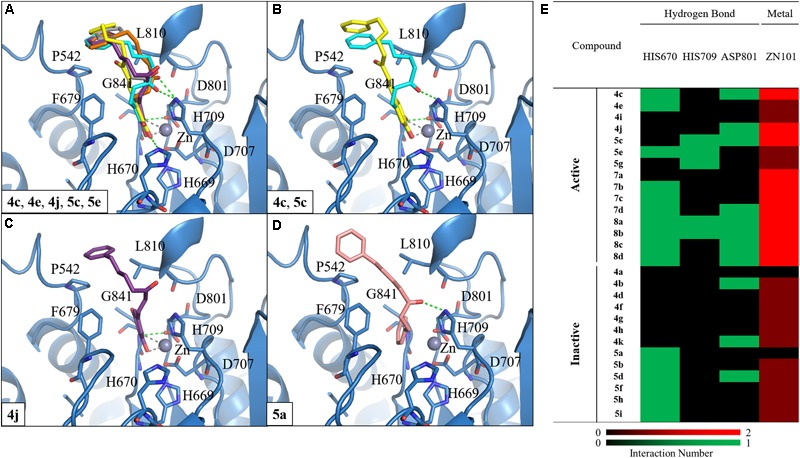
Molecular docking analysis between HDAC7 and compounds. **(A)** Docking poses of compounds **4c** (yellow), **4e** (gray), **4j** (purple), **5c** (blue) and **5e** (orange) in the binding site of HDAC7 structure (PDB ID: 3C10). Docking poses of compounds **(B) 4c, 5c, (C) 4j**, and **(D) 5a**. Hydrogen bonds and metal atom are represented as green dash line and gray sphere, respectively. **(E)** Interaction heatmap of synthesized compounds. Hydrogen bond are colored in green, and metal coordination are colored in red.

We selected HDAC7 as the representative of class IIa HDACs to analyze compound interactions between our synthesized compounds. The compounds were docked into the binding site of HDAC7 (PDB ID: 3C10) with –CDOKER Energy score ranged from 33 to 51. The RMSD analysis revealed that most binding poses are similar (Supplementary Figure S92). The total energy of protein-compound complexes can be found in Supplementary Table S2. The group one active compounds **4c, 4e, 4j, 5c**, and **5e** contain a catechol or pyrogallol group as the ZBGs. The ZBG also facilitates hydrogen bonds for compounds **4c, 4e, 4j**, and **5c** and residue G841. In addition, all active compounds form a hydrogen bond with residue H670 (**Figure 4A**). Residue D801 also form hydrogen bonds with compounds **4c, 4j**, and **5c**. The carbonyl, which is located on the linker, of compound **5c** has a hydrogen bond interaction with residue H709. In addition, the benzene functions as the cap to create hydrophobic interactions with surface residues P542 or L810.

We observed different interactions between our synthesized active compounds and HDAC7. The active compounds **4c, 4j**, and **5c** contain polar contacts to D801, G841 and hydrophobic contacts with P542 and L810. However, compound **5c** forms an extra hydrogen bond with residue H709. This increases the binding ability of compound **5c** (**Figure 4B**). In contrast, compound **4j** only forms hydrophobic interactions with residue H709 (**Figure 4C**). However, compound **4j** is a more potent inhibitor compared to compound **5c** (**Table 2**). The inactive compound **5a** (IC_50_ > 40 μM) does not contain hydroxy groups on the A ring; therefore, it cannot coordinate with the zinc ion within the HDAC7 binding site (**Figures 4D,E**).

**FIGURE 5:**
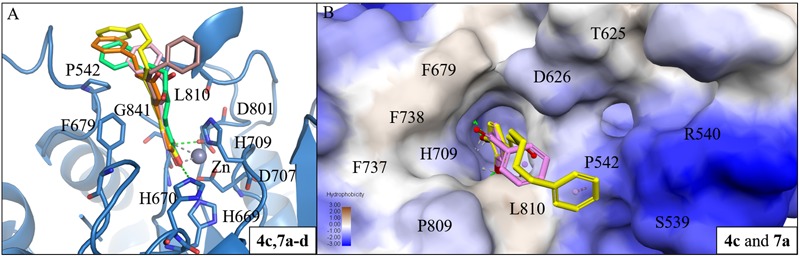
Interaction analysis between HDAC7 and active compounds **4c, 7a**-**d**. **(A)** Docking poses of compounds **4c** (yellow), **7a** (pink), **7b** (brown), **7c** (orange) and **7d** (cyan) in the binding site of HDAC7 (PDB ID: 3C10). Hydrogen bonds and metal atom are represented as green dash line and gray sphere, respectively. **(B)** Surface model of with HDAC7 docked with compounds **4c** and **7a**. Area with log *P* = 3 colored in brown, area with log *P* = -3 colored in blue.

Next, we analyzed the interactions of group two active compounds **7a–d** and **8a–d**. We compared the docking poses of compounds **4c** and **7a**–**d** in HDAC7 (**Figure 5A**). Compound **4c** make hydrophobic contacts with cap residues P542, H709, and L810. In contrast, the shorter linker in compounds **7a–d** does not allow the benzene cap to sufficiently form interactions with cap residue L810. Compounds **7c** and **7d** contain a longer linker, two and three respectively, and were able to form a π-alkyl interaction with residue P542. Previous studies have suggested that the behavior of the cap group plays an important role in HDAC binding affinity (Wang et al., 2005). Thus, the shorter linker in our compounds reduces their ability to sufficiently interact with surface residues of HDAC7 (**Figure 5B**).

#### Interaction Analysis of Compounds in Aβ_1-42_ and HDAC7

**FIGURE 6:**
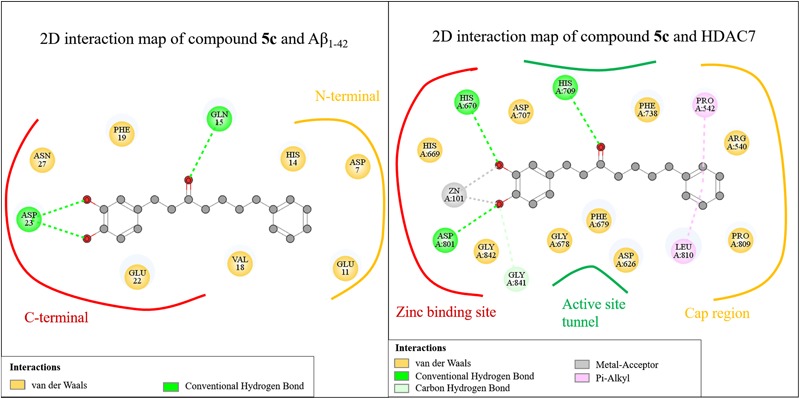
Common interactions of the compounds.

We further analyzed common interactions of the compounds to elucidate their dual inhibitions against Aβ_1-42_ self-aggregation and HDAC7 (**Figure 6**). The interaction analysis of compound **5c** revealed structural features that contributes to the inhibition of Aβ_1-42_ self-aggregation and HDAC7. Compound **5c** contain features typical of HDAC inhibitors, which include a ZBG, a linker and a cap. The catechol moiety not only functions as the ZBG in HDAC7 but generates polar contacts with the electrically charged amino acid residue D23 in Aβ_1-42_. The carbonyl group, located on the linker, generate polar contacts with residue H709 in HDAC7. The carbonyl moiety also creates a hydrogen bond with residue Q15 in Aβ_1-42_. Almost all of the compounds synthesized in this study form hydrogen bonds with this residue in Aβ_1-42_. Finally, the cap is a phenyl ring with a resonance system that created VDW interactions with residue V18 in Aβ_1-42_ and Pi-alkyl interactions with residues P542 and L810 HDAC7. The compounds synthesized in this study may provide an effective design for dual inhibitors of HDAC7 and Aβ_1-42_ self-aggregation.

#### Cytotoxicity of Compounds 4j, 5c, 5e and SAHA

Next, we evaluated the cytotoxicity of the identified compounds. Several studies indicate many methods can be used to evaluate the cytotoxicity, including primary neurons and neuroblastoma cells cultures (Shipley et al., 2016; Marangoni et al., 2018; Peng et al., 2018). Unfortunately, primary neuron cultures are difficult to perform, since mature neurons do not undergo cell division. Previous researchers have used SH-SY5Y cells to test potential neurological drugs. For instance, *Achyranthes bidentata* polypeptides, a potential treatment for Parkinson’s disease, shows 100% viability in SH-SY5Y cells, while rat primary dopaminergic neurons were found to be 95% viable (Peng et al., 2018). Treating SH-SY5Y cells with the neurotoxic agent 1-methyl-4-phenylpyridinium iodide at 100 μM resulted in 80% cell viability, while primary rat mesencephalic neurons were reduced to roughly 75% viability (Luchtman et al., 2013). These results indicate that the cytotoxicity response of compounds in SH-SY5Y cells may be similar to primary cultured neurons. Due to the above reasons, we used SH-SY5Y cells to evaluate the cytotoxicity of our compounds.

**FIGURE 7:**
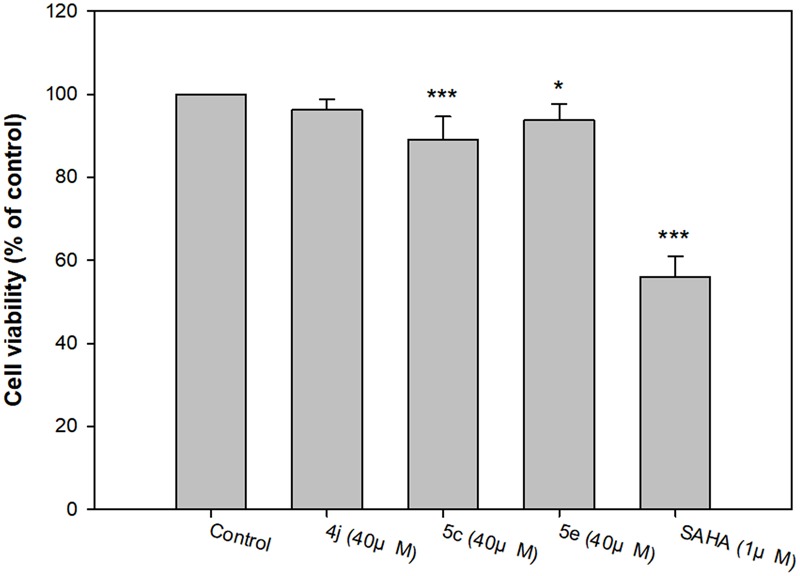
Determination of the viability of compounds **4j, 5c, 5e** and SAHA of human neuroblastoma SH-SY5Y cells by alamar blue assay. All data were expressed as mean ± SD of three experiments and each consisted of six replicates. ^∗^*p* < 0.05, ^∗∗^*p* < 0.01, ^∗∗∗^*p* < 0.001 vs. control. Statistical analysis was performed using one way ANOVA followed by Bonferroni test.

Compounds **4j, 5c**, and **5e** were the most potent inhibitors for HDAC5 or 7, and also showed Aβ_1-42_ self-aggregation inhibitory activity. The HDAC inhibitor SAHA was used as the reference to compare their cytotoxic effects. The cell viability was evaluated by 7-hydroxy-10-oxidophenoxazin-10-ium-3-one (resazurin, alamar blue) assay. As summarized in **Figure 7**, the three compounds did not show significant cytotoxicity at a high concentration (40 μM), which suggested that compounds **4j, 5c**, and **5e** exhibited very low toxicity to SH-SY5Y cells compared to SAHA. In addition, the trypan blue exclusion test also shows the same trends (Supplementary Figure S93). Based on these results, compounds **4j, 5c**, and **5e** were selected to test their neuroprotective effects.

#### Neuroprotective Effect of Compounds 4j, 5c, and 5e on H_2_O_2_-Induced Cell Damage in SH-SY5Y Cells

**FIGURE 8:**
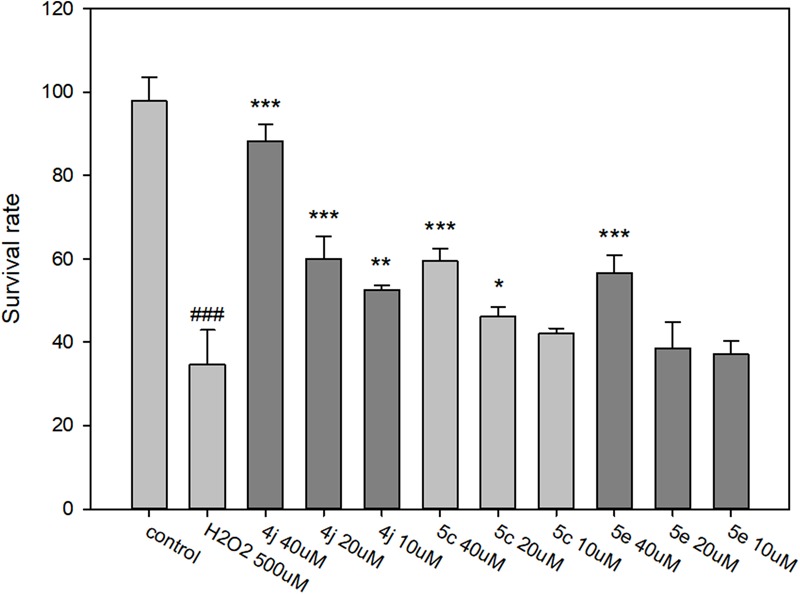
Determination of the neuroprotective effects of compounds **4j, 5c**, and **5e** by MTT assay. The compounds are tested at a concentration of 10, 20, and 40 μM and cell injury is induced by H_2_O_2_ (500 μM) in human neuroblastoma SH-SY5Y cells. All data were expressed as mean ± SD of two experiments and each consisted of quadruplicates. ^#^*p* < 0.05, ^##^*p* < 0.01, ^###^*p* < 0.001 vs. control; ^∗^*p* < 0.05, ^∗∗^*p* < 0.01, ^∗∗∗^*p* < 0.001 vs. H_2_O_2_ alone. Statistical analysis was performed using one way ANOVA followed by Bonferroni test.

The neuroprotective capacities of compounds **4j, 5c**, and **5e** against oxidative stress were evaluated at three different concentrations (40, 20, and 10 μM). After 500 μM H_2_O_2_ exposure, cell viability was markedly decreased to 34.6%, suggesting a high sensitivity to H_2_O_2_ induced SH-SY5Y cell injury (**Figure 8**). Compared to the untreated control, compounds **4j, 5c**, and **5e** showed protective effects in a dose-dependent manner against H_2_O_2_ induced SH-SY5Y cell injury (**Figure 8**). At concentrations of 40 μM, compounds **4j, 5c**, and **5e** indicated significantly neuroprotective effects and the cell viabilities were 88.3, 61.3, and 58.8%, respectively. Moreover, the trypan blue exclusion test also shows the same trends (Supplementary Figure S94). Therefore, compounds **4j, 5c**, and **5e** were able to protect these neurons from toxic stimuli induced by H_2_O_2_.

#### Compounds 4j, 5c, and 5e Have Potential Use as AD Therapeutics

Next, we looked to determine the compounds’ ADMET properties (Supplementary Table S3). The BBB is one of the major impediments for neurological drugs. Compounds **5c** and **5e** was calculated to have a high BBB penetration, whereas compound **4j** had medium penetration. Furthermore, all three compounds showed good human intestinal absorption after oral administration. Finally, the aqueous solubility descriptor predicts the aqueous solubility of each compound in water at 25°C. The three compounds were predicted to be slightly soluble to soluble in water. Thus, compounds **4j, 5c**, and **5e** are predicted to penetrate the BBB when administered orally.

Alzheimer’s disease is a complex disease with multiple pathogenic mechanisms. Aβ_1-42_ aggregation and HDAC overexpression have been shown to be involved in AD pathology. The Aβ_1-42_ isoform displays lower solubility and aggregation, which leads to toxic Aβ oligomers. Class IIa HDACs inhibitors affect the activities of the β-catenin (Margariti et al., 2010) and HSF1 (Calderwood and Murshid, 2017), which plays an important role in AD progression. Therefore, we developed dual inhibitors of Aβ_1-42_ self-aggregation and class IIa HDACs as potential AD treatment. A dual inhibitor has many advantages over a traditional single target inhibitor (Talevi, 2015). Single target drugs for AD treatment only offer a palliative treatment, whereas multi-target drugs have the potential to have an increased synergistic effect. A multi-target drug has a lower risk of drug-drug interactions compared to multicomponent drugs, or traditional drug cocktails (Morphy and Rankovic, 2005). Because of these reasons, we believe that compounds **4j, 5c**, and **5e** show great promise as AD therapeutics. Further studies will be needed to optimize their potency for use as AD treatment.

## Conclusion

Our study sought to develop novel inhibitors that can target both Aβ and HDAC as a potential AD therapeutic. The Aβ hypothesis is the most widely accepted for the progression of AD. The self-aggregation of Aβ leads to the formation of extracellular plaques and neurotoxicity. Additionally, class IIa HDACs are also implicated in AD development. For instance, overexpression of HDAC5 and HDAC7 is associated with various dysregulated biochemical pathways in AD. Due to this complexity, a multi-target drug has great potential for better AD therapeutics. We designed and synthesized a series of diarylheptanoid derivatives with either a catechol or pyrogallol moiety, for Aβ self-aggregation and HDAC inhibition. These moieties have been shown to inhibit Aβ self-aggregation. Compounds **4j, 5c**, and **5e** displayed potent inhibition of both Aβ_1-42_ self-aggregation and of class IIa isozymes HDAC5 and 7, with IC_50_ values of <10 μM. These active compounds contain three common features: (1) a catechol or pyrogallol moiety that not only creates hydrogen bonds with residue D23 of Aβ, but also acts as a zinc binding group for HDAC inhibition, (2) a carbonyl that occupies the hydrophobic tunnel within the HDAC catalytic site that also creates a hydrogen bond with residue Q15 of Aβ_1-42_ structure, and (3) an aromatic ring that creates hydrophobic interactions with the N-terminal of Aβ_1-42_ but also functions as a cap for HDAC class IIa. In addition, compounds with shorter linkers were only able to inhibit Aβ, but not HDAC7. In contrast, compounds with longer linkers, such as compounds **5j, 5c**, and **5e**, showed favorable inhibitory activity for both Aβ and HDAC7. The optimized linker for dual inhibitors were found to be a four carbon chain. Since our interest is in development of a dual inhibitor, these compounds were excluded from further study. Our *in vitro* studies showed no significant cytotoxicity toward SH-SY5Y cells when treated with compounds **4j, 5c**, and **5e**, as well as a neuroprotective effect against H_2_O_2_-induced cell injury. In addition, the predicted BBB penetration, human intestinal absorption and aqueous solubility were favorable for compounds **4j, 5c**, and **5e**. Taken together, these results reveal compounds **4j, 5c**, and **5e** can act as a dual Aβ self-aggregation and class IIa HDACs inhibitors. We believe that these compounds can be used as lead compounds for AD drug development.

## Author Contributions

L-CC, K-CH, and W-JH conceived and designed the experiments. L-CC, H-JT, C-YL, Y-YH, C-CY, and J-RW performed the experiments. L-CC, H-JT, C-YL, J-RW, Y-LL, W-CH, I-HP, K-KH, K-CH, and W-JH analyzed the data. L-CC, C-YL, TL, K-CH, and W-JH wrote the paper.

## Conflict of Interest Statement

The authors declare that the research was conducted in the absence of any commercial or financial relationships that could be construed as a potential conflict of interest.
