# Supplementary material for: Design of Diarylheptanoid Derivatives as Dual Inhibitors Against Class IIa Histone Deacetylase and β-amyloid Aggregation
Source: Front Pharmacol. 2018 Jul 3;9:708. doi: 10.3389/fphar.2018.00708 (PMC6037852; doi:10.3389/fphar.2018.00708)
Supplement: Supplementary file 1 [file Data_Sheet_1.PDF]

Supplementary Material

Design of diarylheptanoid derivatives as dual inhibitors against class IIa histone deacetylase and β-amyloid aggregation

Liang-Chieh Chen, Hui-Ju Tseng, Chang-Yi Liu, Yun-Yi Huang, Cheng-Chung Yen, Jing-Ru Weng, Yeh-Lin Lu, Wen-Chi Hou, Tony Eight Lin, I-Horng Pan, Kuo-Kuei Huang, Wei-Jan Huang* and Kai-Cheng Hsu*

*** Correspondence:** Corresponding Author: piki@tmu.edu.tw (K-CH); wjhuang@tmu.edu.tw (W-JH)

1 The experimental details and physical data of synthetic compounds

1.1.1 (*E*)-6-phenylhex-3-en-2-one (2)

To a solution of 1-(triphenylphosphoranylidene)propan-2-one (14.1 g, 44.7 mmol) in dry DCM (100 mL) was added compound **1** (4.9 mL, 37.3 mmol) dropwise by syringe. The resulting mixture was heated to reflux and stirred for 12 h. The reaction mixture was concentrated in vacuo, diluted with EtOAc (100 mL) and washed with distilled H_2O (3 × 50 mL). The organic layer was dried over Na_2SO_4, filtered and concentrated in vacuo. The residue was purified by silica gel chromatography (EtOAc: *n*-hexane = 1:9) to give α, β-unsaturated ketones **2** (5.1 g, 79%). a colorless oil; ^{1}H NMR (MeOH-d_4, 300 MHz): δ 7.26 (m, 2H), δ 7.18 (m, 3H), δ 6.93 (dt, *J* = 6.8, 16.0 Hz, 1H), δ 6.05 (dt, *J* = 1.5, 16.0 Hz, 1H), δ 2.79 (t, *J* = 7.2 Hz, 2H), δ 2.55 (m, 2H). ESI-MS *m/z*: 175 $(M+H)^+$.

1.1.2 6-phenylhexan-2-one (3)

A catalytic amount of 10% Pd-C (509 mg) was added to a solution of compound **2** (5.1 g, 29.2 mmol) in EtOH (100 mL) and the mixture was stirred at RT under H_2 atmosphere for 5 h. The reaction mixture was filtered with celite and the filtrate was concentrated in vacuo. The residue was purified by silica gel chromatography (EtOAc: *n*-hexane = 1:11) to give α, β-saturated ketones **3** (5.1 g, 99%). a colorless oil; ^{1}H NMR (MeOH-d_4, 300 MHz): δ 7.23 (m, 2H), δ 7.13 (m, 3H), δ 2.60 (t, *J* = 7.1 Hz, 2H), δ 2.48 (t, *J* = 6.8 Hz, 2H), δ 2.09 (s, 3H), δ 1.57 (m, 4H). ESI-MS *m/z*: 177 $(M+H)^+$.

1.1.3 (*E*)-1,7-diphenylhept-1-en-3-one (4a)

To a solution of compound **3** (206 mg, 1.2 mmol) in THF (10 mL) was added pyrrolidine (96 µL, 1.2 mmol), acetic acid (67 µL, 1.2 mmol) and benzaldehyde (83 µL, 0.8 mmol). The mixture was heated to reflux for 3 h. The reaction mixture was diluted with EtOAc (100 mL) and washed with distilled H_2O (3 × 50 mL). The organic layer was dried over Na_2SO_4, filtered and concentrated in vacuo. The residue was purified by silica gel chromatography (EtOAc: *n*-hexane = 1:15) to give diarylheptanoid **4a** (226 mg, 73%). a colorless oil; ^{1}H NMR ($CDCl_3$, 300 MHz): δ 7.58 (d, *J* = 16.2 Hz, 1H), δ 7.58 (m, 2H), δ7.43 (m, 3H), δ 7.33 (m, 2H), δ 7.22 (m, 3H), δ 6.77 (d, *J* = 16.1 Hz, 1H), δ 2.72 (q, *J* = 7.1 Hz, 4H), δ 1.76 (m, 4H); ^{13}C NMR ($CDCl_3$, 125MHz): δ 200.3, 142.4, 142.2, 134.5, 130.4, 128.9, 128.4,

128.3, 128.2, 126.1, 125.7, 40.7, 35.7, 31.0, 23.9. HR-ESI-MS *m/z*: $[M+H]^+$ calcd for $C_{19}H_{21}O$ 265.1587, found 265.1582.

1.1.4 (*E*)-1-(4-hydroxyphenyl)-7-phenylhept-1-en-3-one (4b)

Following the procedure as described for compound **4a**, reaction of compound **3** (110 mg, 0.6 mmol) in THF (6 mL) with pyrrolidine (51 μL, 0.6 mmol), acetic acid (36 μL, 0.6 mmol) and 4-hydroxybenzaldehyde (76 mg, 0.6 mmol) gave compound **4b** (156 mg, 89%). a yellow solid; ^1H NMR (DMSO-*d*6, 300 MHz): δ 10.01 (s, 1H), δ 7.54 (d, *J* = 8.6 Hz, 1H), δ 7.51 (d, *J* = 15.9 Hz, 1H), δ 7.27 (m, 2H), δ 7.16 (m, 3H), δ 6.80 (d, *J* = 8.6 Hz, 1H), δ 6.65 (d, *J* = 16.2 Hz, 1H), δ 2.66 (t, *J* = 6.7 Hz, 2H), δ 2.59 (t, *J* = 7.0 Hz, 2H), δ 1.59 (m, 4H); ^{13}C NMR ($CDCl_3$, 125MHz): δ 201.4, 158.4, 143.0, 142.2, 130.3, 128.4, 128.3, 126.9, 125.7, 123.7, 116.0, 40.5, 35.7, 31.1, 24.2. HR-ESI-MS *m/z*: $[M+H]^+$ calcd for $C_{19}H_{21}O_2$ 281.1536, found 281.1530.

1.1.5 (*E*)-1-(3, 4-dihydroxyphenyl)-7-phenylhept-1-en-3-one (4c)

Following the procedure as described for compound **4a**, reaction of compound **3** (111 mg, 0.6 mmol) in THF (6 mL) with pyrrolidine (52 μL, 0.6 mmol), acetic acid (36 μL, 0.6 mmol) and 3, 4-dihydroxybenzaldehyde (87 mg, 0.6 mmol) gave compound **4c** (94 mg, 50%). a yellow solid; ^1H NMR (DMSO-*d*6, 300 MHz): δ 9.54 (s, 1H), δ 9.18 (s, 1H), δ 7.27 (m, 2H), δ 7.16 (m, 3H), δ 7.06 (d, *J* = 2.0 Hz, 1H), δ 7.00 (dd, *J* = 2.0, 8.2 Hz, 1H), δ 6.77 (d, *J* = 8.1 Hz, 1H), δ 6.64 (d, *J* = 16.1 Hz, 1H), δ 2.65 (t, *J* = 6.8 Hz, 2H), δ 2.59 (t, *J* = 7.0 Hz, 2H), δ 1.57 (m, 4H); ^{13}C NMR ($CDCl_3$, 125MHz): δ 201.9, 147.0, 144.1, 143.7, 142.2, 128.4, 128.3, 127.3, 125.8, 123.8, 122.9, 115.5, 114.4, 40.4, 35.7, 31.0, 24.2. HR-ESI-MS *m/z*: $[M+H]^+$ calcd for $C_{19}H_{21}O_3$ 297.1485, found 297.1479.

1.1.6 (*E*)-1-(3-hydroxyphenyl)-7-phenylhept-1-en-3-one (4d)

Following the procedure as described for compound **4a**, reaction of compound **3** (100 mg, 0.6 mmol) in THF (10 mL) with pyrrolidine (47 μL, 0.6 mmol), acetic acid (32 μL, 0.6 mmol) and 3-hydroxybenzaldehyde (69 mg, 0.6 mmol) gave compound **4d** (54 mg, 34%). a white solid; ^1H NMR (MeOH-d_4, 300 MHz): δ 7.53 (d, *J* = 16.2 Hz, 1H), δ 7.23 (m, 3H), δ 7.13 (m, 3H), δ 7.07 (d, *J* = 7.6 Hz, 1H), δ 7.01 (t, *J* = 2.2 Hz, 1H), δ 6.83 (ddd, *J* = 1.0, 2.5, 8.1 Hz, 1H), δ 6.73 (d, *J* = 16.2 Hz, 1H), δ 2.72 (t, *J* = 7.0 Hz, 2H), δ 2.64 (t, *J* = 7.0 Hz, 2H), δ 1.66 (m, 4H); ^{13}C NMR (DMSO-*d*6, 125MHz): δ 200.0, 157.7, 142.1, 142.0, 135.7, 129.9, 128.2, 128.2, 126.3, 125.6, 119.3, 117.6, 114.6, 39.3, 34.9, 30.5, 23.4. HR-ESI-MS *m/z*: $[M+H]^+$ calcd for $C_{19}H_{21}O_2$ 281.1536, found 281.1533.

1.1.7 (*E*)-1-(2, 3-dihydroxyphenyl)-7-phenylhept-1-en-3-one (4e)

Following the procedure as described for compound **4a**, reaction of compound **3** (300 mg, 1.7 mmol) in THF (16 mL) with pyrrolidine (140 μL, 1.7 mmol), acetic acid (97 μL, 1.7 mmol) and 2, 3-dihydroxybenzaldehyde (157 mg, 1.1 mmol) gave compound **4e** (225 mg, 67%). a brown solid; ^1H NMR (MeOH-d_4, 300 MHz): δ 7.95 (d, *J* = 16.3 Hz, 1H), δ 7.23 (m, 2H), δ 7.16 (m, 3H), δ 7.02 (dd, *J* = 1.4, 7.8 Hz, 1H), δ 6.84 (d, *J* = 16.3 Hz, 1H), δ 6.81 (dd, *J* = 1.5, 7.8 Hz, 1H), δ 6.67 (t, *J* = 7.8 Hz, 1H), δ 2.71 (t, *J* = 7.0 Hz, 2H), δ 2.64 (t, *J* = 7.1 Hz, 2H), δ 1.67 (m, 4H); ^{13}C NMR (DMSO-*d*6, 125MHz): δ 200.0, 145.6, 145.6, 142.1, 137.5, 128.3, 128.2, 125.6, 125.6, 121.6, 119.1, 118.4, 116.8, 39.5, 35.0, 30.5, 23.5. HR-ESI-MS *m/z*: $[M+H]^+$ calcd for $C_{19}H_{21}O_3$ 297.1485, found 297.1482.

1.1.8 (*E*)-1-(3-fluoro-4-hydroxyphenyl)-7-phenylhept-1-en-3-one (4f)

Following the procedure as described for compound **4a**, reaction of compound **3** (100 mg, 0.6 mmol) in THF (10 mL) with pyrrolidine (47 μL, 0.6 mmol), acetic acid (32 μL, 0.6 mmol) and 3-fluoro-4-

hydroxybenzaldehyde (79 mg, 0.6 mmol) gave compound **4f** (125 mg, 74%). a orange solid; ^{1}H NMR (MeOH-d_4, 300 MHz): δ 7.51 (d, *J* = 16.1 Hz, 1H), δ 7.38 (dd, *J* = 2.1, 12.2 Hz, 1H), δ 7.25 (m, 2H), δ 7.15 (m, 4H), δ 6.92 (t, *J* = 8.6 Hz, 1H), δ 6.66 (d, *J* = 16.2 Hz, 1H), δ 2.70 (t, *J* = 7.0 Hz, 2H), δ 2.63 (t, *J* = 7.0 Hz, 2H), δ 1.66 (m, 4H); ^{13}C NMR (DMSO-*d*6, 125MHz): δ 199.7, 152.0, 150.0, 147.3, 147.2, 142.0, 141.2, 141.2, 128.2, 128.2, 126.3, 126.3, 126.0, 126.0 125.6, 124.6, 117.8, 117.8, 115.6, 115.5, 39.3, 34.9, 30.5, 23.5. HR-ESI-MS *m/z*: $[M+H]^+$ calcd for $C_{19}H_{21}O_3F$ 299.1442, found 299.1440.

1.1.9 (*E*)-1-(3-chloro-4-hydroxyphenyl)-7-phenylhept-1-en-3-one (4g)

Following the procedure as described for compound **4a**, reaction of compound **3** (100 mg, 0.6 mmol) in THF (10 mL) with pyrrolidine (47 μL, 0.6 mmol), acetic acid (32 μL, 0.6 mmol) and 3-chloro-4-hydroxybenzaldehyde (89 mg, 0.6 mmol) gave compound **4g** (61 mg, 34%). a light yellow solid; ^{1}H NMR (MeOH-d_4, 300 MHz): δ 7.60 (d, *J* = 2.2 Hz, 1H), δ 7.49 (d, *J* = 16.2 Hz, 1H), δ 7.41 (dd, *J* = 2.1, 8.4 Hz, 1H), δ 7.23 (m, 2H), δ 7.15 (m, 3H), δ 6.93 (d, *J* = 8.4 Hz, 1H), δ 6.66 (d, *J* = 16.1 Hz, 1H), δ 2.70 (t, *J* = 7.0 Hz, 2H), δ 2.63 (t, *J* = 7.0 Hz, 2H), δ 1.65 (m, 4H); ^{13}C NMR (DMSO-*d*6, 125MHz): δ 199.7, 155.8, 142.0, 141.0, 130.0, 128.6, 128.2, 128.2, 126.2, 125.6, 124.2, 120.5, 117.0, 39.3, 34.9, 30.5, 23.5. HR-ESI-MS *m/z*: $[M+H]^+$ calcd for $C_{19}H_{21}O_2Cl$ 315.1146, found 315.1142.

1.1.10 (*E*)-1-(3-bromo-4-hydroxyphenyl)-7-phenylhept-1-en-3-one (4h)

Following the procedure as described for compound **4a**, reaction of compound **3** (100 mg, 0.6 mmol) in THF (10 mL) with pyrrolidine (47 μL, 0.6 mmol), acetic acid (32 μL, 0.6 mmol) and 3-bromo-4-hydroxybenzaldehyde (114 mg, 0.6 mmol) gave compound **4h** (110 mg, 54%). a light yellow solid; ^{1}H NMR (MeOH-d_4, 300 MHz): δ 7.76 (d, *J* = 2.1 Hz, 1H), δ 7.48 (d, *J* = 16.1 Hz, 1H), δ 7.45 (dd, *J* = 2.1, 8.6 Hz, 1H), δ 7.23 (m, 2H), δ 7.13 (m, 3H), δ 6.90 (d, *J* = 8.4 Hz, 1H), δ 6.65 (d, *J* = 16.1 Hz, 1H), δ 2.69 (t, *J* = 7.0 Hz, 2H), δ 2.63 (t, *J* = 7.0 Hz, 2H), δ 1.65 (m, 4H); ^{13}C NMR (DMSO-*d*6, 125MHz): δ 199.7, 156.1, 142.0, 140.7, 133.2, 129.1, 128.2, 128.2, 127.2, 125.6, 124.5, 116.5, 109.9, 39.3, 34.9, 30.5, 23.5. HR-ESI-MS *m/z*: $[M+H]^+$ calcd for $C_{19}H_{21}O_2Br$ 359.0641, found 359.0638.

1.1.11 (*E*)-1-(4-hydroxy-3-nitrophenyl)-7-phenylhept-1-en-3-one (4i)

Following the procedure as described for compound **4a**, reaction of compound **3** (100 mg, 0.6 mmol) in THF (10 mL) with pyrrolidine (47 μL, 0.6 mmol), acetic acid (32 μL, 0.6 mmol) and 3-nitro-4-hydroxybenzaldehyde (95 mg, 0.6 mmol) gave compound **4i** (109 mg, 59%). a yellow solid; ^{1}H NMR (MeOH-d_4, 300 MHz): δ 8.29 (d, *J* = 2.2 Hz, 1H), δ 7.90 (dd, *J* = 2.2, 8.8 Hz, 1H), δ 7.58 (d, *J* = 16.3 Hz, 1H), δ 7.24 (m, 2H), δ 7.11 (m, 4H), δ 6.79 (d, *J* = 16.1 Hz, 1H), δ 2.73 (t, *J* = 7.0 Hz, 2H), δ 2.64 (t, *J* = 7.0 Hz, 2H), δ 1.67 (m, 4H); ^{13}C NMR (DMSO-*d*6, 125MHz): δ 199.8, 153.7, 142.0, 140.0, 137.3, 133.9, 128.2, 128.2, 125.8, 125.7, 125.7, 125.6, 119.7, 39.3, 34.9, 30.5, 23.5. HR-ESI-MS *m/z*: $[M+H]^+$ calcd for $C_{19}H_{20}O_4N$ 326.1387, found 326.1384.

1.1.12 (*E*)-1-(3, 4, 5-trihydroxyphenyl)-7-phenylhept-1-en-3-one (4j)

Following the procedure as described for compound **4a**, reaction of compound **3** (100 mg, 0.6 mmol) in THF (10 mL) with pyrrolidine (47 μL, 0.6 mmol), acetic acid (32 μL, 0.6 mmol) and 3,4,5-trihydroxybenzaldehyde (98 mg, 0.6 mmol) gave compound **4j** (85 mg, 48%). a brown solid; ^{1}H NMR (MeOH-d_4, 300 MHz): δ 7.40 (d, *J* = 16.1 Hz, 1H), δ 7.23 (m, 2H), δ 7.14 (m, 3H), δ 6.64 (s, 2H), δ 6.53 (d, *J* = 16.0 Hz, 1H), δ 2.67 (t, *J* = 6.9 Hz, 2H), δ 2.62 (m, 2H), δ 1.65 (m, 4H); ^{13}C NMR (DMSO-*d*6, 125MHz): δ 199.6, 146.1, 143.1, 142.1, 136.4, 128.3, 128.2, 125.6, 124.7, 123.2, 107.7, 39.0, 34.9, 30.7, 30.5, 23.6. HR-ESI-MS *m/z*: $[M+H]^+$ calcd for $C_{19}H_{21}O_4$ 313.1434, found 313.1431.

1.1.13 (*E*)-1-(4-hydroxy-3-methoxyphenyl)-7-phenylhept-1-en-3-one (4k, yakuchinone B)

Following the procedure as described for compound **4a**, reaction of compound **3** (86 mg, 0.5 mmol) in THF (6 mL) with pyrrolidine (40 μL, 0.5 mmol), acetic acid (28 μL, 0.5 mmol) and 3-methoxy-4-hydroxybenzaldehyde (89 mg, 0.6 mmol) gave compound **4k** (95 mg, 62 %). a yellow solid; ^{1}H NMR (DMSO-d_6, 300 MHz): δ 9.60 (s, 1H), δ 7.50 (d, *J* = 16.2 Hz, 1H), δ 7.27 (m, 3H), δ 7.18 (m, 3H), δ 7.13 (dd, *J* = 2.0, 8.1 Hz, 1H), δ 6.80 (d, *J* = 8.2 Hz, 1H), δ 6.72 (d, *J* = 16.2 Hz, 1H), δ 2.67 (t, *J* = 6.8 Hz, 2H), δ 2.60 (t, *J* = 7.0 Hz, 2H), δ 1.59 (m, 4H); ^{13}C NMR ($CDCl_3$, 125MHz): δ 200.4, 148.1, 146.8, 142.7, 142.2, 128.4, 128.3, 127.0, 125.7, 124.0, 123.4, 114.8, 109.4, 55.9, 40.4, 35.7, 31.1, 24.1. HR-ESI-MS *m/z*: $[M+H]^+$ calcd for $C_{20}H_{23}O_3$ 311.1642, found 311.1635.

1.1.14 1, 7-diphenylheptan-3-one (5a)

Following the procedure as described for compound **3**, reaction of compound **4a** (35 mg, 0.1 mmol), 10% Pd-C (4 mg) in EtOH (5 mL) under H_2 atmosphere gave compound **5a** (35 mg, 97 %). a colorless oil; ^{1}H NMR ($CDCl_3$, 300 MHz): δ 7.34 (m, 2H), δ 7.31 (m, 2H), δ 7.25 (m, 3H), δ 7.21 (m, 3H), δ 2.94 (t, *J* = 7.1 Hz, 2H), δ 2.76 (t, *J* = 7.1 Hz, 1H), δ 2.65 (t, *J* = 7.2 Hz, 1H), δ 2.45 (t, *J* = 7.0 Hz, 2H), δ 1.65 (m, 4H); ^{13}C NMR ($CDCl_3$, 125MHz): δ 210.0, 142.1, 141.1, 128.4, 128.3, 128.3, 128.3, 126.0, 125.7, 44.2, 42.8, 35.7, 30.9, 29.7, 23.4. HR-ESI-MS *m/z*: $[M+H]^+$ calcd for $C_{19}H_{23}O$ 267.1743, found 267.1738.

1.1.15 1-(4-hydroxyphenyl)-7-phenylheptan-3-one (5b)

Following the procedure as described for compound **3**, reaction of compound **4b** (54 mg, 0.2 mmol), 10% Pd-C (6 mg) in EtOH (5 mL) under H_2 atmosphere gave compound **5b** (52 mg, 95 %). a yellow solid; ^{1}H NMR (DMSO-d_6, 300 MHz): δ 9.11 (s, 1H), δ 7.27 (m, 2H), δ 7.15 (m, 3H), δ 6.96 (d, *J* = 8.5 Hz, 2H), δ 6.64 (d, *J* = 8.5 Hz, 2H), δ 2.64 (s, 4H), δ 2.54 (t, *J* = 7.1 Hz, 2H), δ 2.41 (t, *J* = 7.1 Hz, 2H), δ 1.47 (m, 4H); ^{13}C NMR ($CDCl_3$, 125MHz): δ 210.5, 153.9, 142.2, 133.2, 129.4, 128.4, 128.3, 125.7, 115.3, 44.5, 42.9, 35.7, 30.9, 28.9, 23.4. HR-ESI-MS *m/z*: $[M+H]^+$ calcd for $C_{19}H_{23}O_2$ 283.1693, found 283.1688.

1.1.16 1-(3, 4-dihydroxyphenyl)-7-phenylheptan-3-one (5c)

Following the procedure as described for compound **3**, reaction of compound **4c** (51 mg, 0.2 mmol), 10% Pd-C (6 mg) in EtOH (5 mL) under H_2 atmosphere gave compound **5c** (51 mg, 99 %). a yellow solid; ^{1}H NMR (Acetone-d_6, 300 MHz): δ 7.71 (s, 1H), 7.68 (s, 1H), δ 7.28 (m, 2H), δ 7.18 (m, 3H), δ 6.75 (d, *J* = 8.0 Hz, 1H), δ 6.72 (d, *J* = 2.1 Hz, 1H), δ 6.55 (dd, *J* = 2.0, 8.0, 1H), δ2.71 (m, 4H), δ 2.62 (t, *J* = 7.1 Hz, 2H), δ 2.47 (t, *J* = 7.0 Hz, 2H), δ 1.59 (m, 4H); ^{13}C NMR ($CDCl_3$, 125MHz): δ 211.4, 143.6, 142.1, 141.9, 133.9, 128.4, 128.4, 128.3, 128.2, 125.7, 120.5, 115.4, 115.3, 44.4, 42.9, 35.7, 30.9, 29.1, 23.3. HR-ESI-MS *m/z*: $[M+H]^+$ calcd for $C_{19}H_{23}O_3$ 299.1642, found 299.1638.

1.1.17 1-(3-hydroxyphenyl)-7-phenylheptan-3-one (5d)

Following the procedure as described for compound **3**, reaction of compound **4d** (90 mg, 0.3 mmol), 10% Pd-C (9 mg) in EtOH (5 mL) under H_2 atmosphere gave compound **5d** (70 mg, 77 %). a light yellow oil; ^{1}H NMR (MeOH-d_4, 300 MHz): δ 7.23 (m, 2H), δ 7.12 (m, 3H), δ 7.04 (d, *J* = 8.3 Hz, 1H), δ 6.60 (m, 3H), δ 2.72 (m, 4H), δ 2.56 (t, *J* = 7.1 Hz, 2H), δ 2.42 (t, *J* = 7.0 Hz, 2H), δ 1.54 (m, 4H); ^{13}C NMR (DMSO-d_6, 125MHz): δ 209.7, 157.2, 142.6, 142.0, 129.1, 128.2, 128.2, 125.6, 118.7, 115.1, 112.7, 43.2, 41.6, 34.9, 30.4, 29.1, 22.7. HR-ESI-MS *m/z*: $[M+H]^+$ calcd for $C_{19}H_{23}O_2$ 283.1693, found 283.1699.

1.1.18 1-(2, 3-dihydroxyphenyl)-7-phenylheptan-3-one (5e)

Following the procedure as described for compound **3**, reaction of compound **4e** (100 mg, 0.3 mmol), 10% Pd-C (10 mg) in EtOH (5 mL) under H_2 atmosphere gave compound **5e** (79 mg, 78 %). a brown solid; ^{1}H NMR (MeOH-d_4, 300 MHz): δ 7.22 (m, 2H), δ 7.12 (m, 3H), δ 6.62 (dd, *J* = 1.2, 4.5 Hz, 1H), δ 6.55 (t, *J* = 4.5 Hz, 1H), δ 6.52 (dd, *J* = 1.2, 4.5 Hz, 1H), δ 2.80 (t, *J* = 4.2 Hz, 2H), δ 2.72 (t, *J* = 4.2 Hz, 2H), δ 2.56 (t, *J* = 4.2 Hz, 2H), δ 2.43 (t, *J* = 4.1 Hz, 2H),δ 1.54 (m, 4H); ^{13}C NMR (DMSO-d_6, 125MHz): δ 210.2, 144.8, 143.0, 142.0, 128.2, 18.2, 127.9, 125.6, 120.0, 118.6, 113.2, 42.0, 41.5, 34.9, 30.4, 24.2, 22.8. HR-ESI-MS *m/z*: $[M+H]^+$ calcd for $C_{19}H_{23}O_3$ 299.1642, found 299.1639.

1.1.19 1-(3-fluoro-4-hydroxyphenyl)-7-phenylheptan-3-one (5f)

Following the procedure as described for compound **3**, reaction of compound **4f** (90 mg, 0.3 mmol), 10% Pd-C (9 mg) in EtOH (5 mL) under H_2 atmosphere gave compound **5f** (55 mg, 61 %). a white solid; ^{1}H NMR (MeOH-d_4, 300 MHz): δ 7.23 (m, 2H), δ 7.11 (m, 3H), δ 6.87 (m, 1H), δ 6.79 (m, 2H), δ 2.71 (m, 4H), δ 2.56 (t, *J* = 7.0 Hz, 2H), δ 2.42 (t, *J* = 7.0 Hz, 2H), δ 1.54 (m, 4H); ^{13}C NMR (DMSO-d_6, 125MHz): δ 209.7, 151.7, 149.8, 142.7, 142.6, 142.0, 132.5, 132.5, 128.2, 128.2, 125.6, 124.1, 124.1, 117.4, 117.4, 115.8, 115.6, 43.3, 41.6, 34.9, 30.4, 28.0, 22.7. HR-ESI-MS *m/z*: $[M+H]^+$ calcd for $C_{19}H_{22}O_2F$ 301.1598, found 301.1596.

1.1.20 1-(3-chloro-4-hydroxyphenyl)-7-phenylheptan-3-one (5g)

Following the procedure as described for compound **3**, reaction of compound **4g** (100 mg, 0.3 mmol), 10% Pd-C (10 mg) in EtOH (5 mL) under H_2 atmosphere gave compound **5g** (37 mg, 37 %). a white solid; ^{1}H NMR (MeOH-d_4, 300 MHz): δ 7.22 (m, 2H), δ 7.11 (m, 4H), δ 6.92 (dd, *J* = 2.1, 8.2 Hz, 1H), δ 6.79 (d, *J* = 8.3 Hz, 1H), δ 2.70 (m, 4H), δ 2.56 (t, *J* = 7.0 Hz, 2H), δ 2.42 (t, *J* = 7.0 Hz, 2H), δ 1.53 (m, 4H); ^{13}C NMR (DMSO-d_6, 125MHz): δ 209.7, 151.0, 142.0, 132.9, 129.3, 128.2, 128.2, 127.7, 125.6, 119.2, 116.4, 43.3, 41.6, 34.9, 30.4, 27.9, 22.7. HR-ESI-MS *m/z*: $[M+H]^+$ calcd for $C_{19}H_{22}O_2Cl$ 317.1303, found 317.1301.

1.1.21 1-(3-bromo-4-hydroxyphenyl)-7-phenylheptan-3-one (5h)

Following the procedure as described for compound **3**, reaction of compound **4h** (100 mg, 0.3 mmol), 10% Pd-C (10 mg) in EtOH (5 mL) under H_2 atmosphere gave compound **5g** (39 mg, 39 %). a white solid; ^{1}H NMR (MeOH-d_4, 300 MHz): δ 7.27 (d, *J* = 2.1 Hz, 1H), δ 7.23 (m, 2H), δ 7.11 (m, 3H), δ 6.96 (dd, *J* = 2.2, 8.2 Hz, 1H), δ 6.77 (d, *J* = 8.3 Hz, 1H), δ 2.69 (m, 4H), δ 2.56 (t, *J* = 7.0 Hz, 2H), δ 2.41 (t, *J* = 7.0 Hz, 2H), δ 1.53 (m, 4H); ^{13}C NMR (DMSO-d_6, 125MHz): δ 209.7, 152.0, 142.0, 133.4, 132.3, 128.4, 128.2, 128.2, 125.6, 116.1, 108.9, 43.3, 41.6, 34.9, 30.4, 27.8, 22.7. HR-ESI-MS *m/z*: $[M+H]^+$ calcd for $C_{19}H_{22}O_2Br$ 361.0798, found 361.0796.

1.1.22 1-(4-hydroxy-3-methoxyphenyl)-7-phenylheptan-3-one (5i, yakuchinone A)

Following the procedure as described for compound **3**, reaction of compound **4k** (95 mg, 0.3 mmol), 10% Pd-C (10 mg) in EtOH (10 mL) under H_2 atmosphere gave compound **5i** (96 mg, 99 %). a light yellow oil; ^{1}H NMR ($CDCl_3$, 300 MHz): δ 7.31 (m, 2H), δ 7.21 (m, 3H), δ 6.86 (d, *J* = 7.9 Hz, 1H), δ 6.69 (m, 1H), δ 2.69 (m, 4H), δ 3.89 (s, 3H), δ 2.85 (t, *J* = 7.2 Hz, 2H), δ 2.71 (t, *J* = 7.2 Hz, 2H), δ 2.64 (t, *J* = 6.9 Hz, 2H), δ 2.43 (t, *J* = 6.8 Hz, 2H),δ 1.64 (m, 4H); ^{13}C NMR ($CDCl_3$, 125MHz): δ 210.3, 146.3, 143.8, 142.1, 133.0, 128.3, 128.3, 125.7, 120.7, 114.3, 111.0, 55.8, 44.6, 42.9, 35.7, 30.9, 29.5, 23.4. HR-ESI-MS *m/z*: $[M+H]^+$ calcd for $C_{20}H_{25}O_3$ 313.1798, found 313.1791.

1.1.23 1-phenylpropan-2-one (6b)

To a mixture of compound **9** (500 mg, 3.95 mmol) and $PdCl_2(PPh_3)_2$ (277 mg, 0.40 mmol) in toluene (5 mL)was added tributyl(1-ethoxyvinyl)tin (1.5 mL, 4.35 mmol). The resulting solution was heated to 80 °C and stirred for 20 h. After cooling to RT, the reaction mixture was acidified with 1 M HCl (5 mL) and stirred for 3 h. The mixture was diluted with EtOAc (30 mL) and washed with distilled H_2O (3 × 30 mL). The organic layer was dried over Na_2SO_4, filtered and concentrated in vacuo. The residue was purified by silica gel chromatography (EtOAc:n-hexane = 1:9) to give compound **6b** (421 mg, 79%), a colorless oil; ^{1}H NMR (MeOH-d_4, 300 MHz): δ 7.31 (m, 2H), δ 7.23 (m, 3H), δ 3.73 (s, 2H), δ 2.13 (s, 3H); ESI-MS *m/z*: 135 $(M+H)^+$.

1.1.24 (*E*)-5-phenylpent-3-en-2-one (11)

Following the procedure as described for compound **2**, a solution of 1-(triphenylphosphoranylidene)propan-2-one (636 mg, 2.0 mmol) and compound **10** (200 mg, 1.7 mmol) in dry DCM (10 mL) was heated to reflux to give compound **11** (213 mg, 87 %). a yellow oil; ^{1}H NMR (Acetone-d_6, 300 MHz): δ 7.32 (m, 2H), δ 7.23 (m, 3H), δ 6.98 (dt, J = 6.9, 15.9 Hz, 1H), δ 6.05 (dt, J = 1.6, 15.9 Hz, 1H), δ 3.58 (dd, J = 1.5, 6.9 Hz, 1H), δ 2.20 (s, 3H); ESI-MS *m/z*: 161 $(M+H)^+$.

1.1.25 5-phenylpentan-2-one (6d)

Following the procedure as described for compound **3**, reaction of compound **11** (194 mg, 0.3 mmol), 10% Pd-C (20 mg) in EtOH (10 mL) under H_2 atmosphere gave compound **6d** (187 mg, 95 %). a colorless oil; ^{1}H NMR (MeOH-d_4, 300 MHz): δ 7.25 (m, 2H), δ 7.13 (m, 3H), δ 2.59 (t, J = 7.4 Hz, 2H), δ 2.47 (t, J = 7.3 Hz, 2H), δ 2.09 (s, 3H), δ 1.84 (m, 2H); ESI-MS *m/z*: 163 $(M+H)^+$.

1.1.26 (*E*)-3-(3, 4-dihydroxyphenyl)-1-phenylprop-2-en-1-one (7a)

Following the procedure as described for compound **4a**, reaction of compound **6a** (200 mg, 1.7 mmol) in THF (6 mL) with pyrrolidine (137 μL, 1.7 mmol), acetic acid (95 μL, 1.7 mmol) and 3,4-dihydroxybenzaldehyde (115 mg, 0.8 mmol) gave compound **7a** (145 mg, 73%). a yellow solid; ^{1}H NMR (Acetone-d_6, 300 MHz): δ 8.53 (s, 1H), δ 8.11 (m, 2H), δ 7.66 (m, 2H), δ 7.57 (m, 3H), δ 7.34 (d, J = 2.1 Hz, 1H), δ 7.21 (dd, J = 1.8, 8.2 Hz, 1H), δ 6.91 (d, J = 8.2 Hz, 1H); ^{13}C NMR (DMSO-*d*6, 125MHz): δ 189.0, 148.8, 145.6, 145.0, 138.0, 128.7, 128.3, 126.2, 122.2, 118.4, 115.7, 115.5. HR-ESI-MS *m/z*: $[M+H]^+$ calcd for $C_{15}H_{13}O_3$ 241.0859, found 241.0856.

1.1.27 (*E*)-4-(3, 4-dihydroxyphenyl)-1-phenylbut-3-en-2-one (7b)

Following the procedure as described for compound **4a**, reaction of compound **6b** (142 mg, 1.1 mmol) in THF (5 mL) with pyrrolidine (87 μL, 1.1 mmol), acetic acid (60 μL, 1.1 mmol) and 3,4-dihydroxybenzaldehyde (97 mg, 0.7 mmol) gave compound **7b** (51 mg, 29%). a yellow solid; ^{1}H NMR (Acetone-d_6, 300 MHz): δ 7.56 (d, J = 16.0 Hz, 1H), δ 7.27 (m, 5H), δ 7.15 (d, J = 2.0 Hz, 1H), δ 7.05 (dd, J = 2.0, 8.3 Hz, 1H), δ 6.86 (d, J = 8.2 Hz, 1H), δ 6.68 (d, J = 16.1 Hz, 1H), δ 3.95 (s, 2H); ^{13}C NMR (DMSO-*d*6, 125MHz): δ 196.9, 148.6, 145.6, 143.7, 135.4, 129.5, 128.3, 126.4, 125.7, 122.5, 121.8, 115.8, 114.8, 46.7. HR-ESI-MS *m/z*: $[M+H]^+$ calcd for $C_{16}H_{15}O_3$ 255.1016, found 255.1012.

1.1.28 (*E*)-1-(3, 4-dihydroxyphenyl)-5-phenylpent-1-en-3-one (7c)

Following the procedure as described for compound **4a**, reaction of compound **6c** (200 mg, 1.3 mmol) in THF (6 mL) with pyrrolidine (110 μL, 1.3 mmol), acetic acid (77 μL, 1.3 mmol) and 3,4-dihydroxybenzaldehyde (93 mg, 0.7 mmol) gave compound **7c** (130 mg, 72%). a yellow solid; ^{1}H

NMR (Acetone-d_6, 300 MHz): δ 8.44 (s, 1H), δ 8.15 (s, 1H), δ 7.51 (d, *J* = 16.1 Hz, 1H), δ 7.27 (d, *J* = 4.4 Hz, 4H), δ 7.17 (m, 2H), δ 7.06 (dd, *J* = 2.2, 8.2 Hz, 1H), δ 6.87 (d, *J* = 8.2 Hz, 1H), δ 6.63 (d, *J* = 16.2 Hz, 1H), δ 2.96 (m, 4H); ^{13}C NMR (DMSO-*d*6, 125MHz): δ 198.5, 148.2, 145.4, 142.7, 141.2, 128.1, 128.0, 125.6, 125.6, 122.8, 121.4, 115.5, 114.6, 40.9, 29.4. HR-ESI-MS *m/z*: $[M+H]^+$ calcd for $C_{17}H_{17}O_3$ 269.1172, found 269.1168.

1.1.29 (*E*)-1-(3, 4-dihydroxyphenyl)-6-phenylhex-1-en-3-one (7d)

Following the procedure as described for compound **4a**, reaction of compound **6d** (125 mg, 0.8 mmol) in THF (5 mL) with pyrrolidine (63 µL, 0.8 mmol), acetic acid (44 µL, 0.8 mmol) and 3,4-dihydroxybenzaldehyde (71 mg, 0.5 mmol) gave compound **7d** (104 mg, 72%). a yellow solid; ^{1}H NMR (Acetone-d_6, 300 MHz): δ 8.39 (s, 1H), δ 8.20 (s, 1H), δ 7.46 (d, *J* = 16.1 Hz, 1H), δ 7.26 (m, 4H), δ 7.18 (m, 2H), δ 7.05 (dd, *J* = 2.3, 8.2 Hz, 1H), δ 6.87 (d, *J* = 8.2 Hz, 1H), δ 6.62 (d, *J* = 16.1 Hz, 1H) , δ 2.67 (m, 4H), δ 1.94 (m, 2H); ^{13}C NMR (DMSO-*d*6, 125MHz): δ 199.4, 148.4, 145.6, 142.7, 141.8, 128.3, 128.3, 125.8, 125.8, 123.0, 121.6, 115.8, 114.8, 39.0, 34.6, 25.8. HR-ESI-MS *m/z*: $[M+H]^+$ calcd for $C_{18}H_{19}O_3$ 283.1329, found 283.1325.

1.1.30 3-(3, 4-dihydroxyphenyl)-1-phenylpropan-1-one (8a)

Following the procedure as described for compound **3**, reaction of compound **7a** (50 mg, 0.2 mmol), 10% Pd-C (5 mg) in EtOH (5 mL) under H_2 atmosphere gave compound **8a** (30 mg, 60 %). a light yellow solid; ^{1}H NMR (DMSO-d_6, 300 MHz): δ 8.68 (s, 1H), δ 8.61 (s, 1H), δ 7.96 (m, 2H), δ 7.62 (m, 1H), δ 7.51 (m, 2H), δ 6.63 (s, 1H), δ 6.61 (d, *J* = 7.9 Hz, 1H), δ 6.49 (dd, *J* = 2.0, 8.0 1H), δ 3.26 (t, *J* = 7.3 Hz, 2H), δ 2.76 (t, *J* = 7.4 Hz, 2H); ^{13}C NMR (DMSO-d_6, 125MHz): δ 199.4, 145.0, 143.3, 136.7, 133.1, 132.0, 128.7, 127.9, 118.9, 115.8, 115.4, 39.8, 28.9. HR-ESI-MS *m/z*: $[M+H]^+$ calcd for $C_{15}H_{15}O_3$ 243.1016, found 243.1015.

1.1.31 4-(3, 4-dihydroxyphenyl)-1-phenylbutan-2-one (8b)

Following the procedure as described for compound **3**, reaction of compound **7b** (40 mg, 0.2 mmol), 10% Pd-C (5 mg) in EtOH (5 mL) under H_2 atmosphere gave compound **8b** (21 mg, 52 %). a light yellow solid; ^{1}H NMR (DMSO-d_6, 300 MHz): δ 8.69 (s, 1H), δ 8.61 (s, 1H), δ 7.28 (m, 3H), δ 7.16 (m, 2H), δ 6.59 (d, *J* = 8.0 Hz, 1H), δ 6.54 (d, *J* = 2.1 1H), δ 6.39 (dd, *J* = 2.1, 8.0 1H), δ 3.73 (s, 2H), δ 2.71 (t, *J* = 6.8 Hz, 2H), δ 2.58 (t, *J* = 6.8 Hz, 2H); ^{13}C NMR (DMSO-d_6, 125MHz): δ 207.4, 145.0, 143.3, 134.9, 131.8, 129.6, 128.3, 126.5, 118.7, 115.6, 115.4, 48.8, 43.5, 28.6. HR-ESI-MS *m/z*: $[M-H]^-$ calcd for $C_{16}H_{15}O_3$ 255.1016, found 255.1019.

1.1.32 1-(3, 4-dihydroxyphenyl)-5-phenylpentan-3-one (8c)

Following the procedure as described for compound **3**, reaction of compound **7c** (50 mg, 0.2 mmol), 10% Pd-C (5 mg) in EtOH (5 mL) under H_2 atmosphere gave compound **8c** (33 mg, 66 %). a light yellow solid; ^{1}H NMR (DMSO-d_6, 300 MHz): δ 8.69 (s, 1H), δ 8.60 (s, 1H), δ 7.26 (m, 2H), δ 7.15 (m, 3H), δ 6.60 (d, *J* = 8.0 Hz, 1H), δ 6.55 (d, *J* = 2.1 Hz, 1H), δ 6.40 (dd, *J* = 2.1, 8.0 1H), δ 2.73 (m, 4H), δ 2.62 (m, 4H); ^{13}C NMR (DMSO-d_6, 125MHz): δ 209.2, 145.0, 143.3, 141.2, 131.9, 128.2, 128.2, 125.8, 118.7, 115.6, 115.4, 43.8, 43.4, 29.0, 28.5. HR-ESI-MS *m/z*: $[M+H]^+$ calcd for $C_{17}H_{19}O_3$ 271.1329, found 271.1324.

1.1.33 1-(3, 4-dihydroxyphenyl)-6-phenylhexan-3-one (8d)

Following the procedure as described for compound **3**, reaction of compound **7d** (40 mg, 0.1 mmol), 10% Pd-C (5 mg) in EtOH (5 mL) under H_2 atmosphere gave compound **8c** (27 mg, 67 %). a light

yellow solid; ^{1}H NMR (DMSO-d_6, 300 MHz): δ 8.68 (s, 1H), δ 8.60 (s, 1H), δ 7.27 (m, 2H), δ 7.16 (m, 3H), δ 6.60 (d, *J* = 8.0 Hz, 1H), δ 6.55 (d, *J* = 2.1 Hz, 1H), δ 6.40 (dd, *J* = 2.1, 8.0 1H), δ 2.60 (m, 4H), δ 2.50 (m, 8H), δ 2.39 (t, *J* = 7.2 Hz, 2H), δ 1.73 (m, 2H); ^{13}C NMR (DMSO-d_6, 125MHz): δ 209.9, 145.0, 143.3, 141.7, 131.9, 128.3, 125.8, 118.7, 115.6, 115.4, 43.8, 41..3, 34.4, 28.6, 25.0. HR-ESI-MS *m/z*: $[M+H]^+$ calcd for $C_{18}H_{21}O_3$ 285.1485, found 285.1481.

2 Trypan blue exclusion test

The cell viability was also evaluated by trypan blue exclusion test as described previously (Degenhardt et al., 2002;Zanoni et al., 2016;Pan et al., 2017). The cells were detaching from each well by trypsinization. The suspended cells and 0.5% trypan blue solution (BioWest) were mixed 1: 1 and the cells number were counted by hemocytometer.

3 NMR and HPLC data

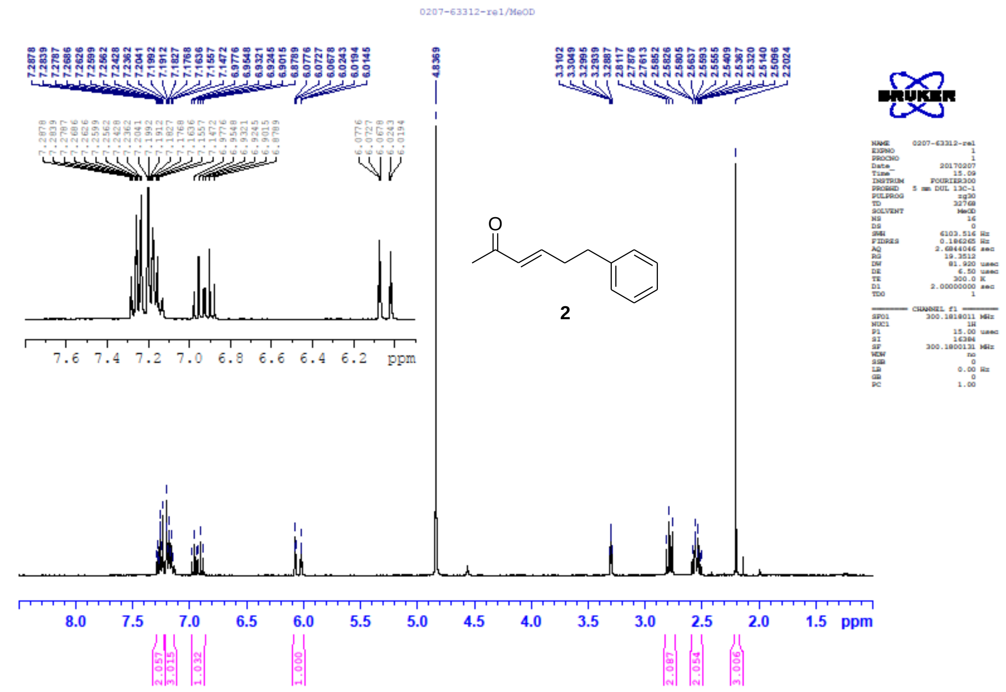

Supplementary Figure S1. ^{1}H NMR (MeOH-d_4, 300 MHz) spectrum of compound **2**

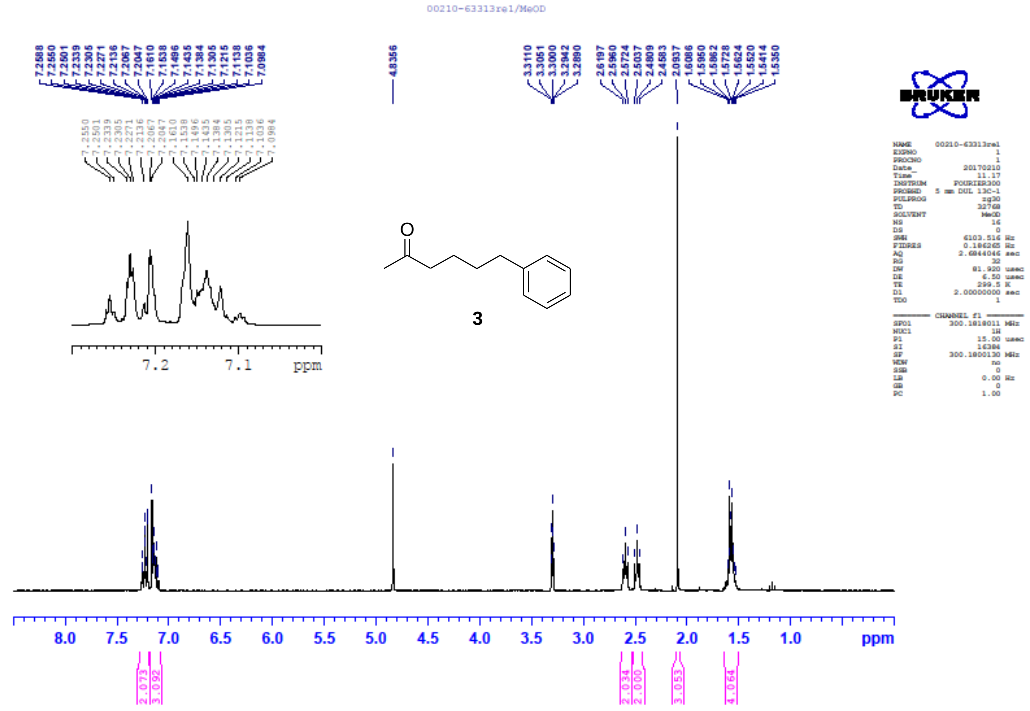

Supplementary Figure S2. ^{1}H NMR (MeOH-d_4, 300 MHz) spectrum of compound **3**

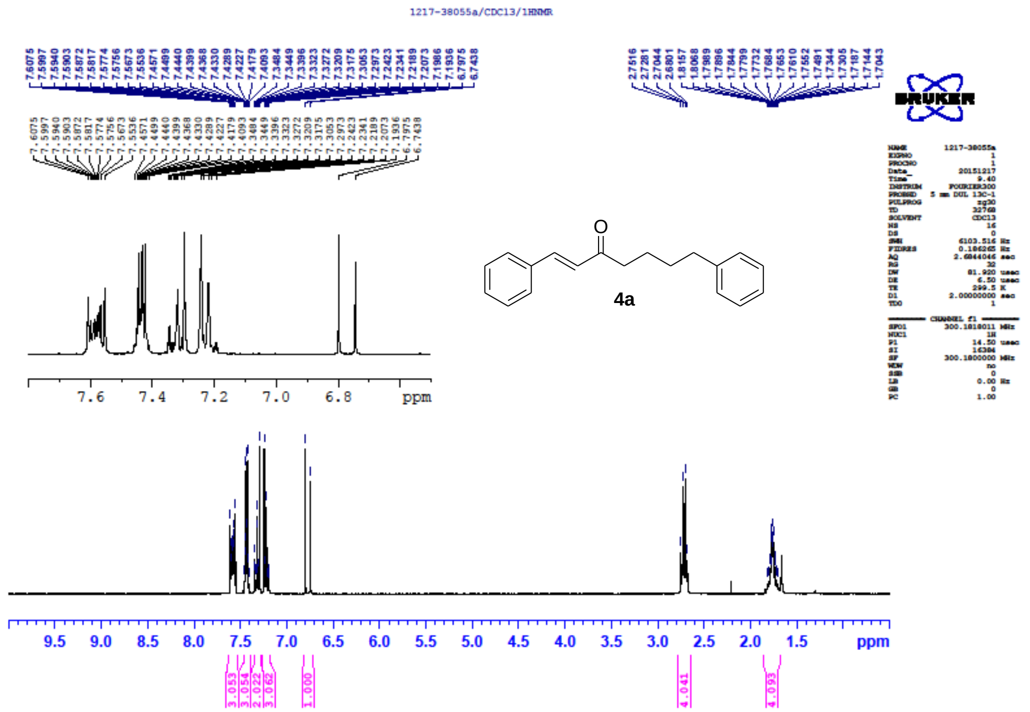

Supplementary Figure S3. ^{1}H NMR ($CDCl_3$, 300 MHz) spectrum of compound **4a**

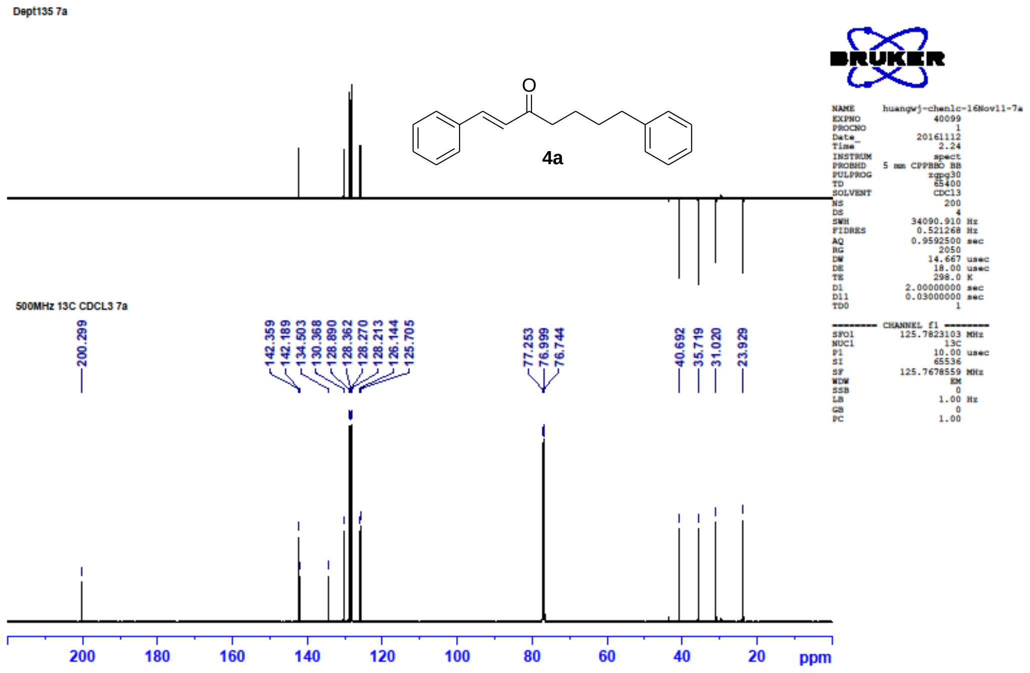

Supplementary Figure S4. ^{13}C NMR ($CDCl_3$, 125 MHz) spectrum of compound **4a**

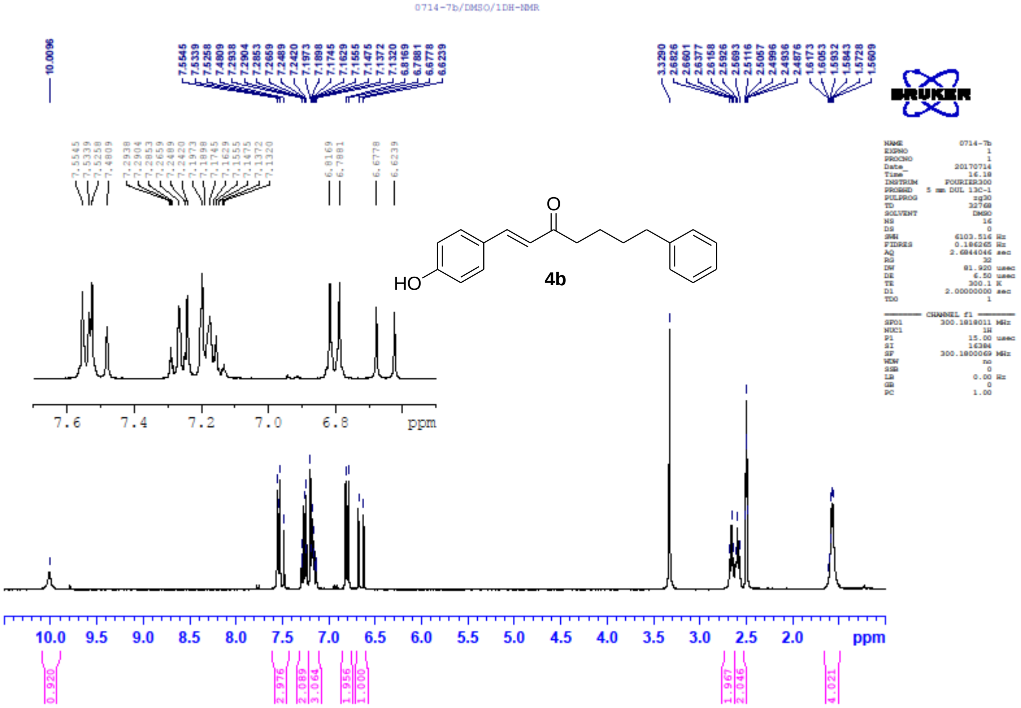

Supplementary Figure S5. ^{1}H NMR (DMSO-d_6, 300 MHz) spectrum of compound **4b**

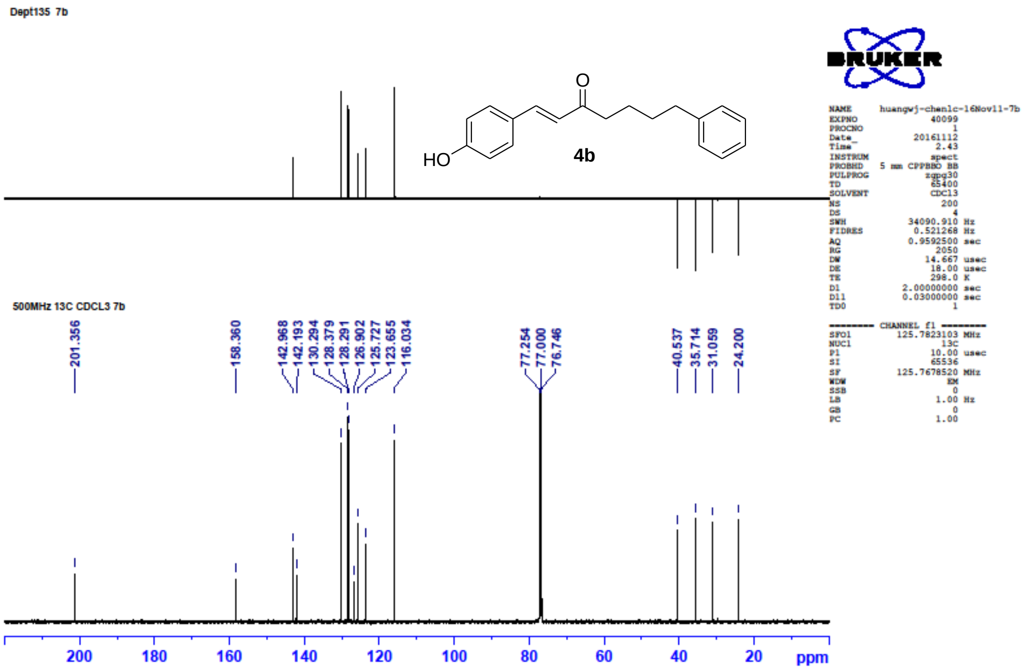

Supplementary Figure S6. ^{13}C NMR ($CDCl_3$, 125 MHz) spectrum of compound **4b**

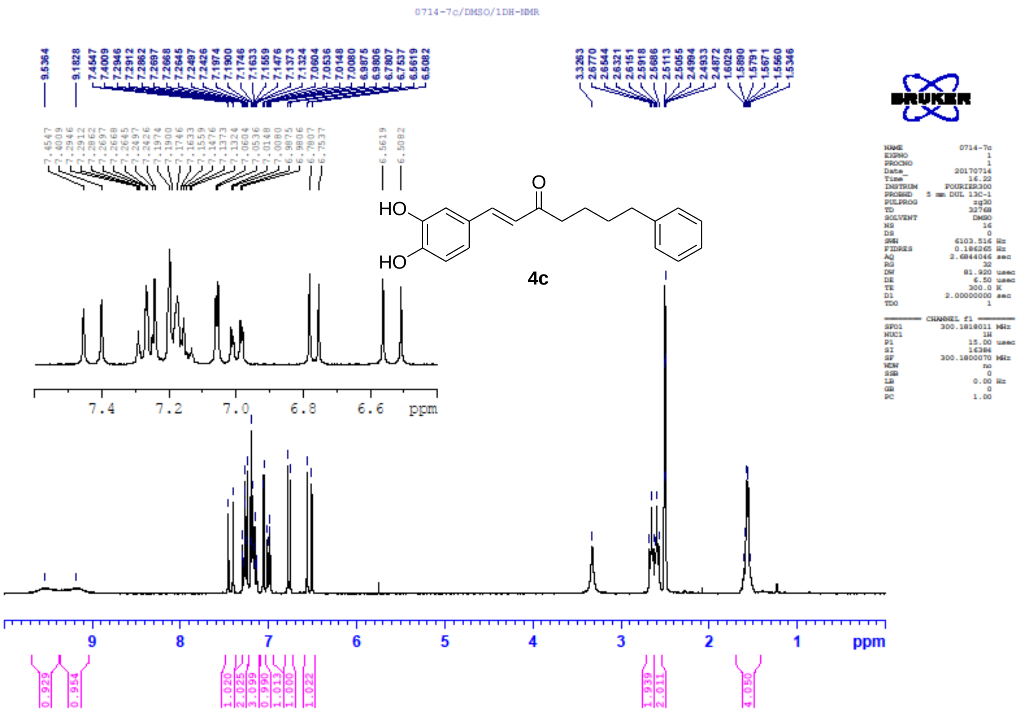

Supplementary Figure S7. ^{1}H NMR (DMSO-*d*6, 300 MHz) spectrum of compound **4c**

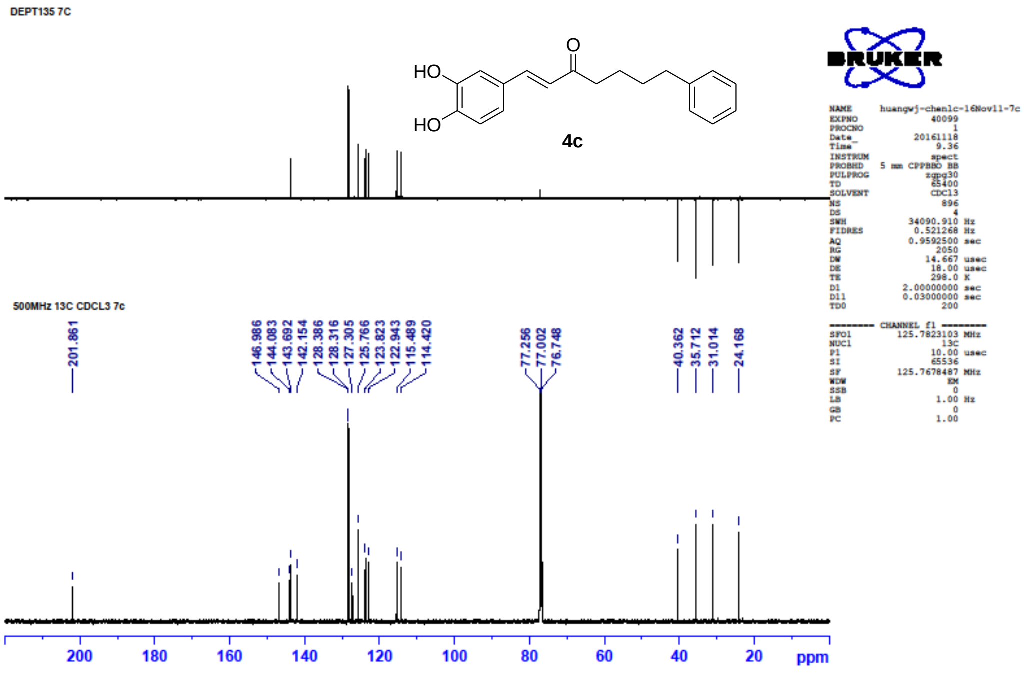

Supplementary Figure S8. ^{13}C NMR ($CDCl_3$, 125 MHz) spectrum of compound **4c**

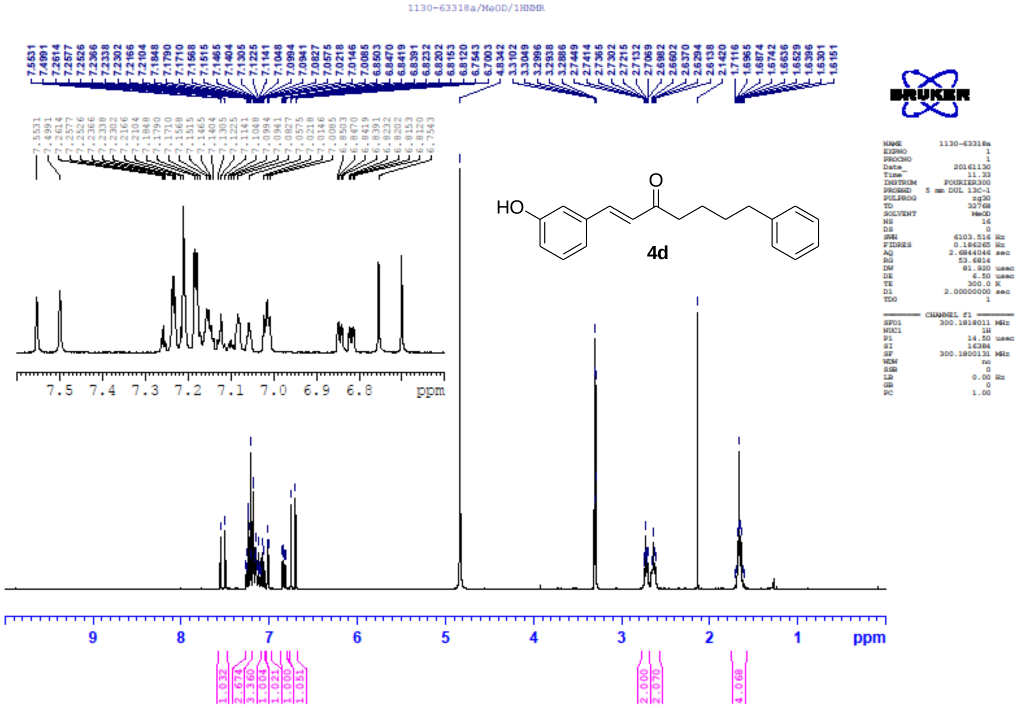

Supplementary Figure S9. ^{1}H NMR (MeOH-d_4, 300 MHz) spectrum of compound **4d**

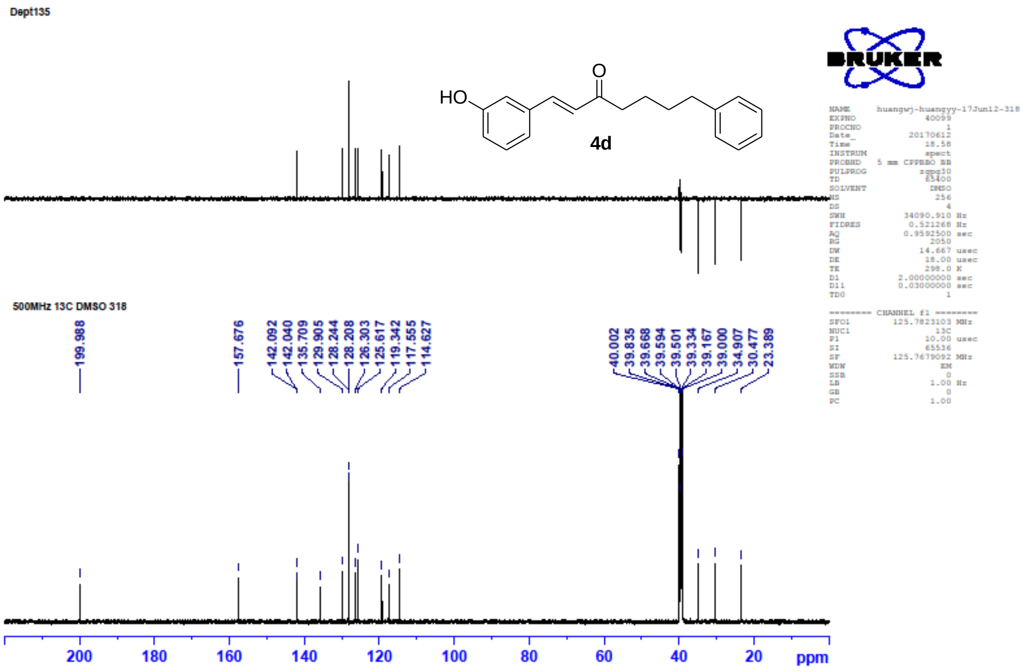

Supplementary Figure S10. ^{13}C NMR (DMSO-d_6, 125 MHz) spectrum of compound **4d**

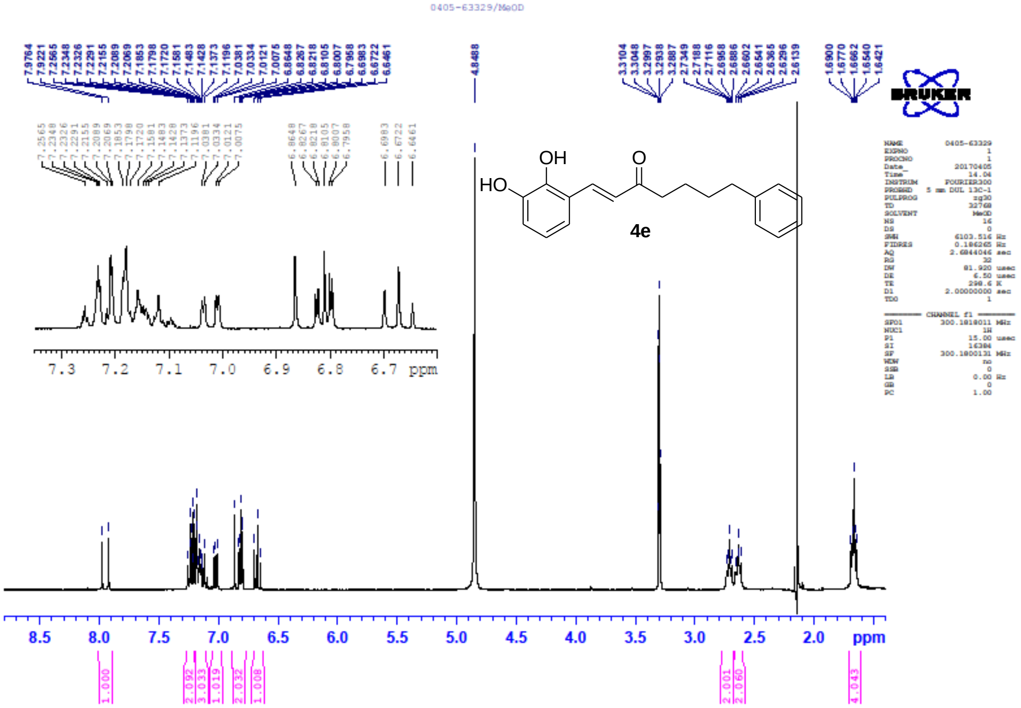

Supplementary Figure S11. ^{1}H NMR (MeOH-d_4, 300 MHz) spectrum of compound **4e**

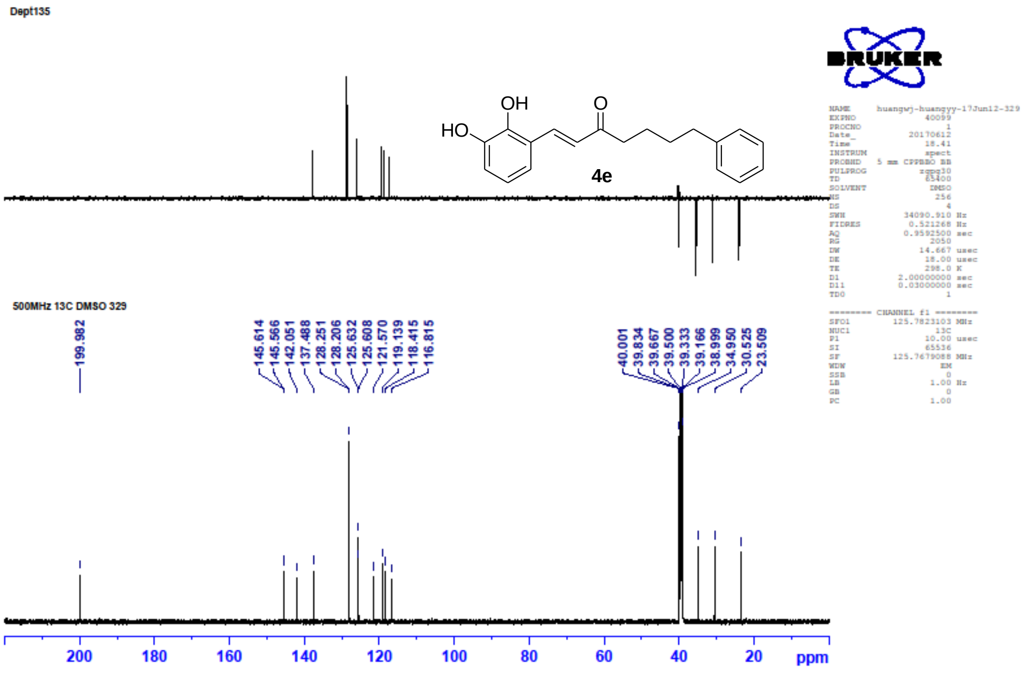

Supplementary Figure S12. ^{13}C NMR (DMSO-d_6, 125 MHz) spectrum of compound **4e**

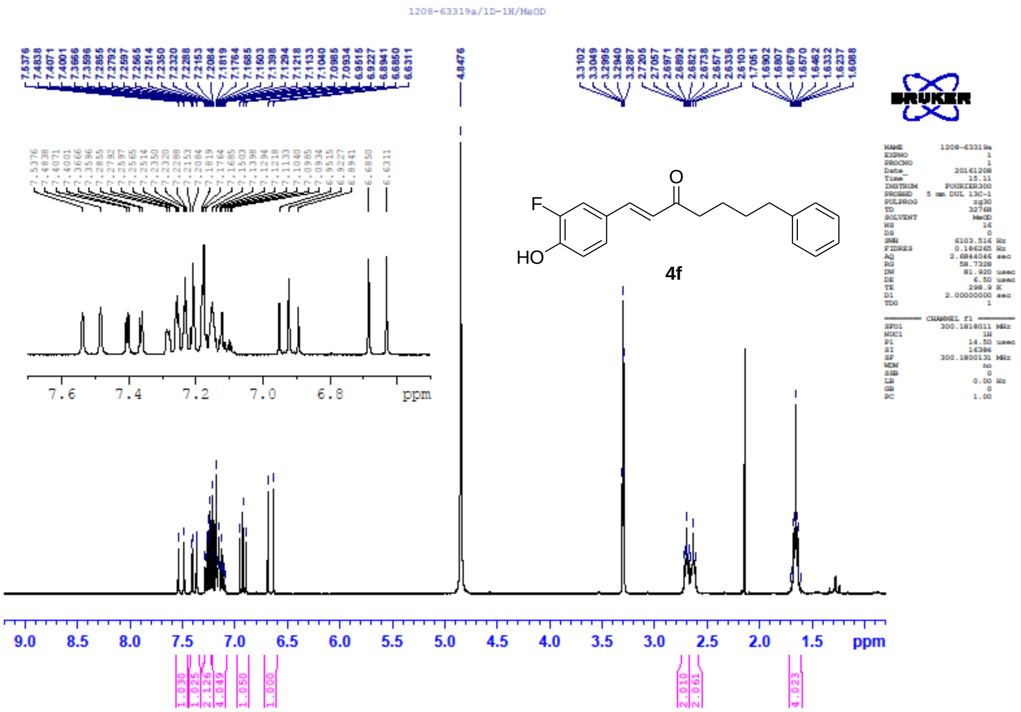

Supplementary Figure S13. ^{1}H NMR (MeOH-d_4, 300 MHz) spectrum of compound **4f**

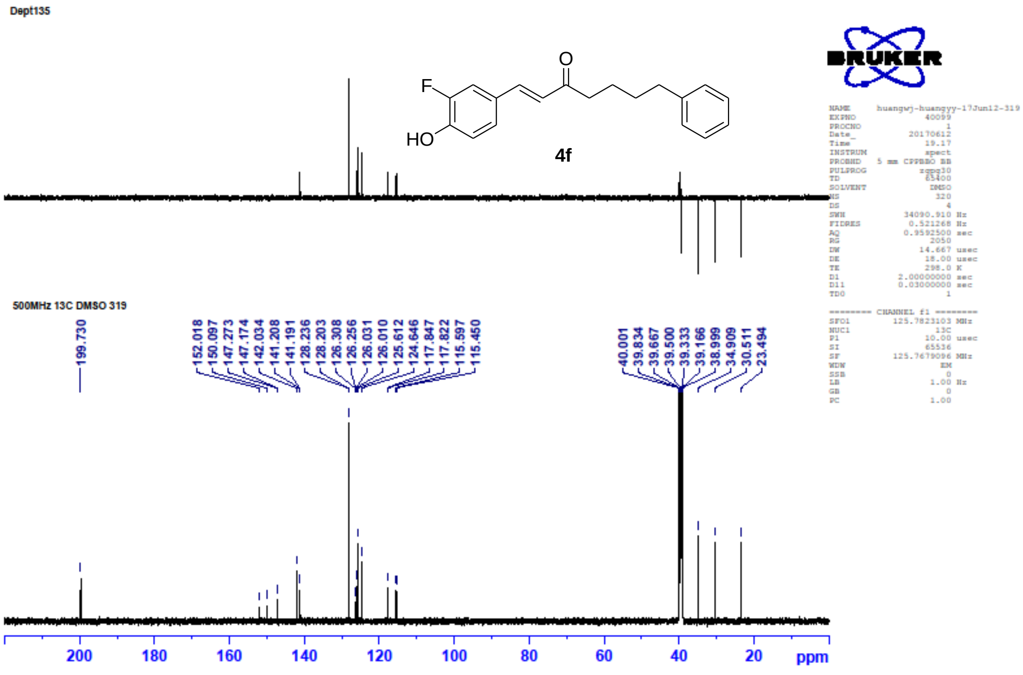

Supplementary Figure S14. ^{13}C NMR (DMSO-d_6, 125 MHz) spectrum of compound **4f**

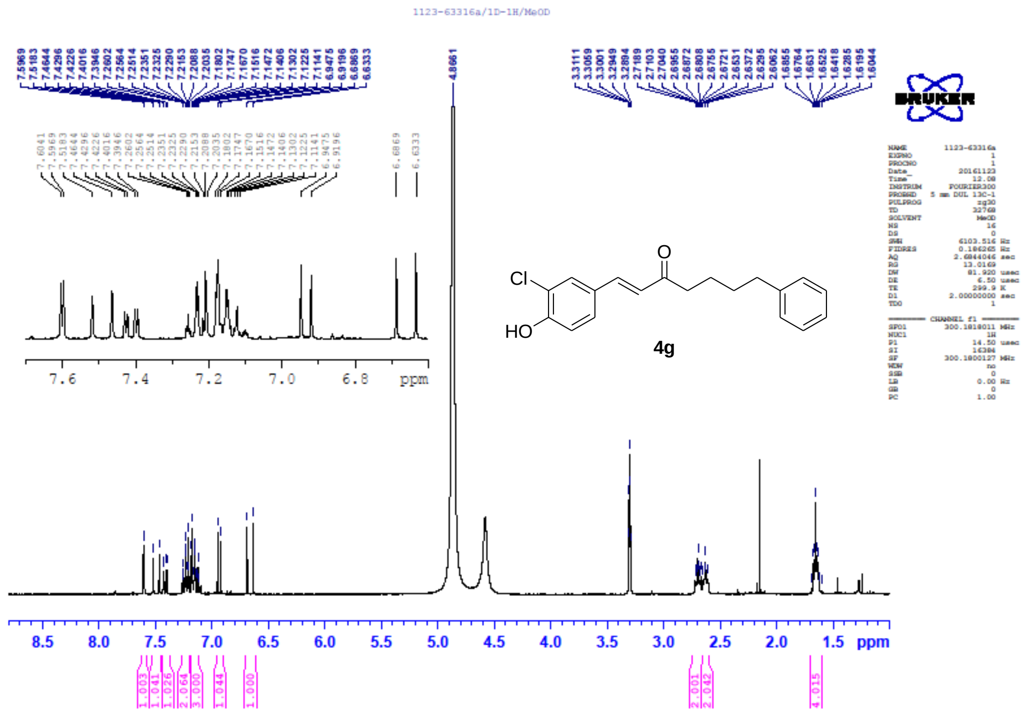

Supplementary Figure S15. ^{1}H NMR (MeOH-d_4, 300 MHz) spectrum of compound **4g**

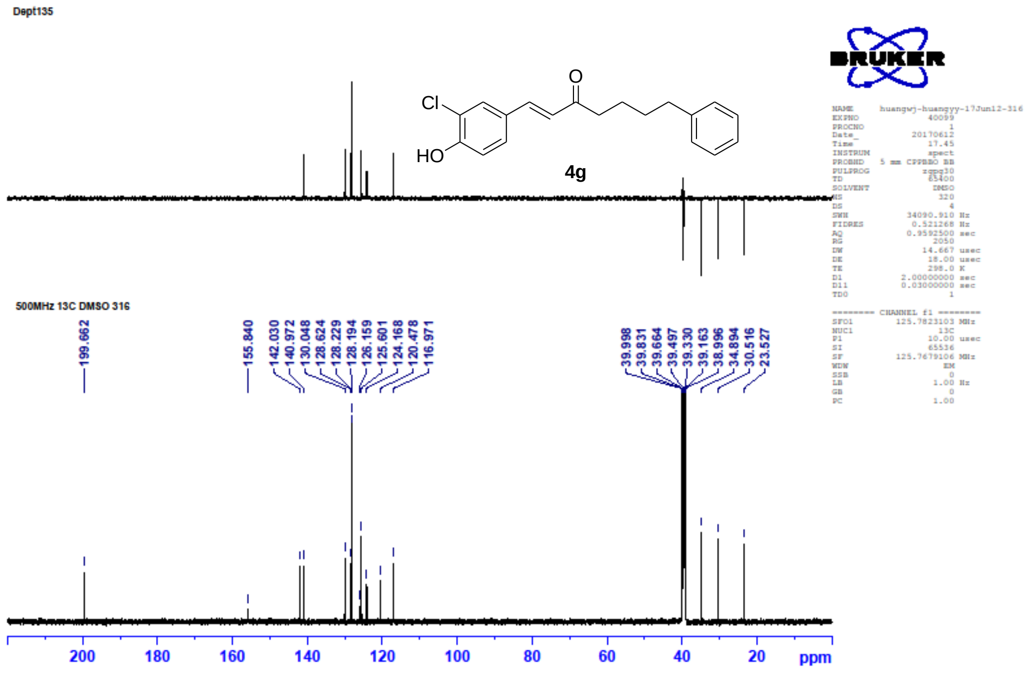

Supplementary Figure S16. ^{13}C NMR (DMSO-d_6, 125 MHz) spectrum of compound **4g**

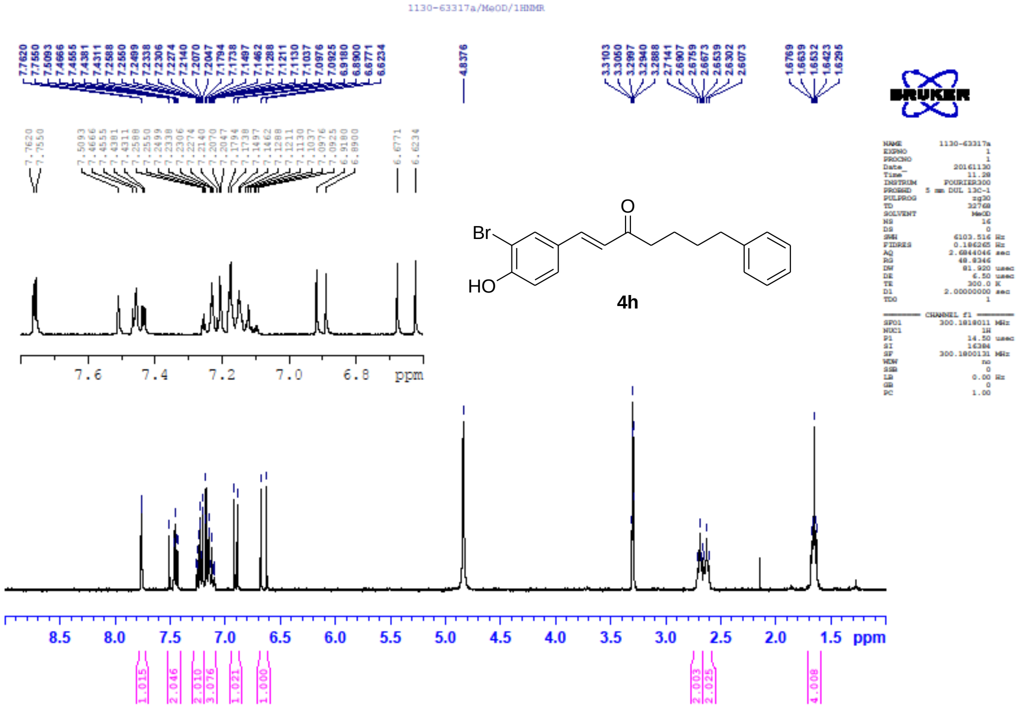

Supplementary Figure S17. ^{1}H NMR (MeOH-d_4, 300 MHz) spectrum of compound **4h**

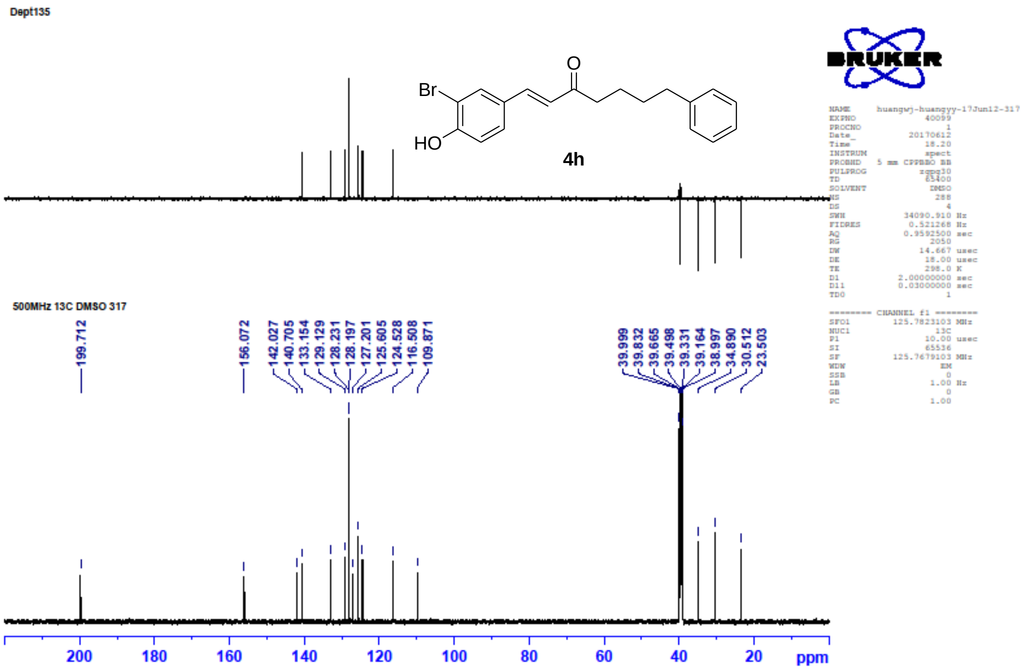

Supplementary Figure S18. ^{13}C NMR (DMSO-d_6, 125 MHz) spectrum of compound **4h**

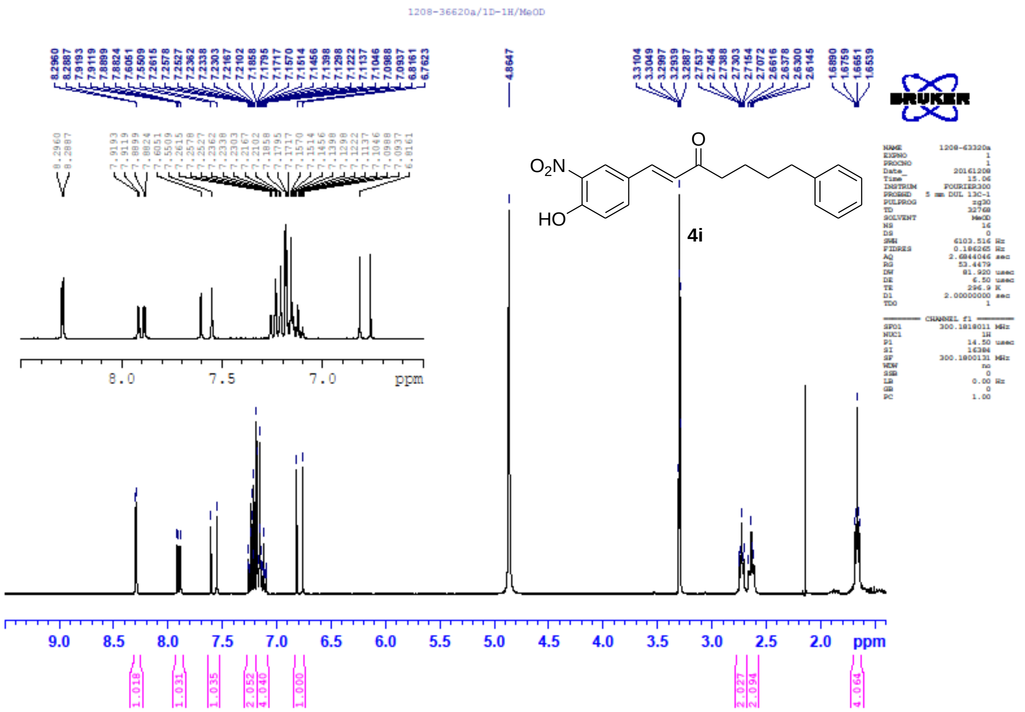

Supplementary Figure S19. ^{1}H NMR (MeOH-d_4, 300 MHz) spectrum of compound **4i**

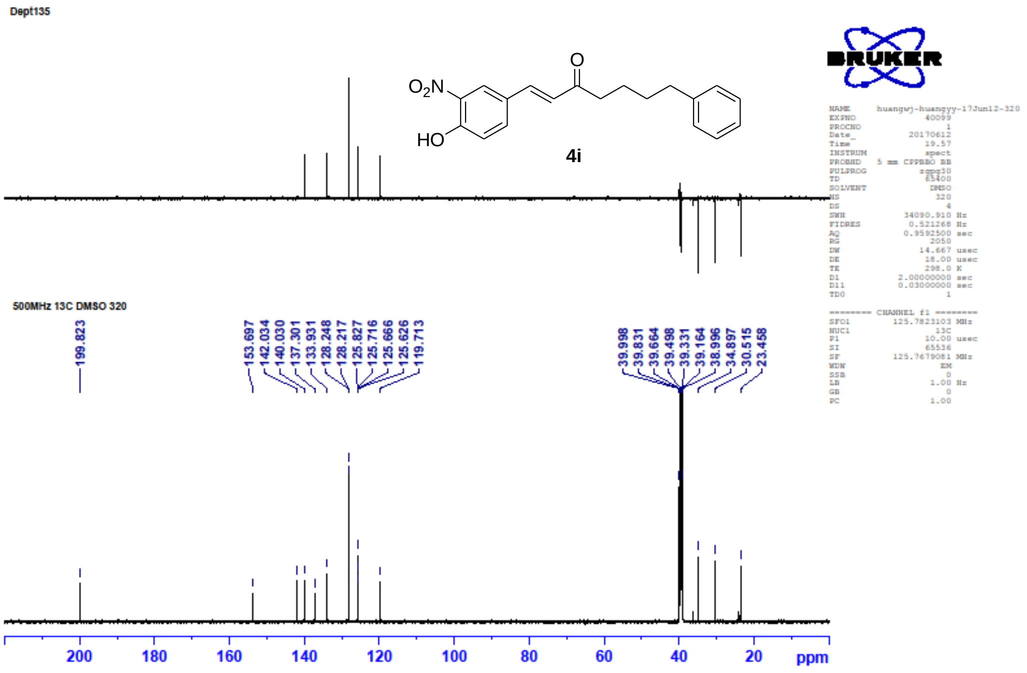

Supplementary Figure S20. ^{13}C NMR (DMSO-d_6, 125 MHz) spectrum of compound **4i**

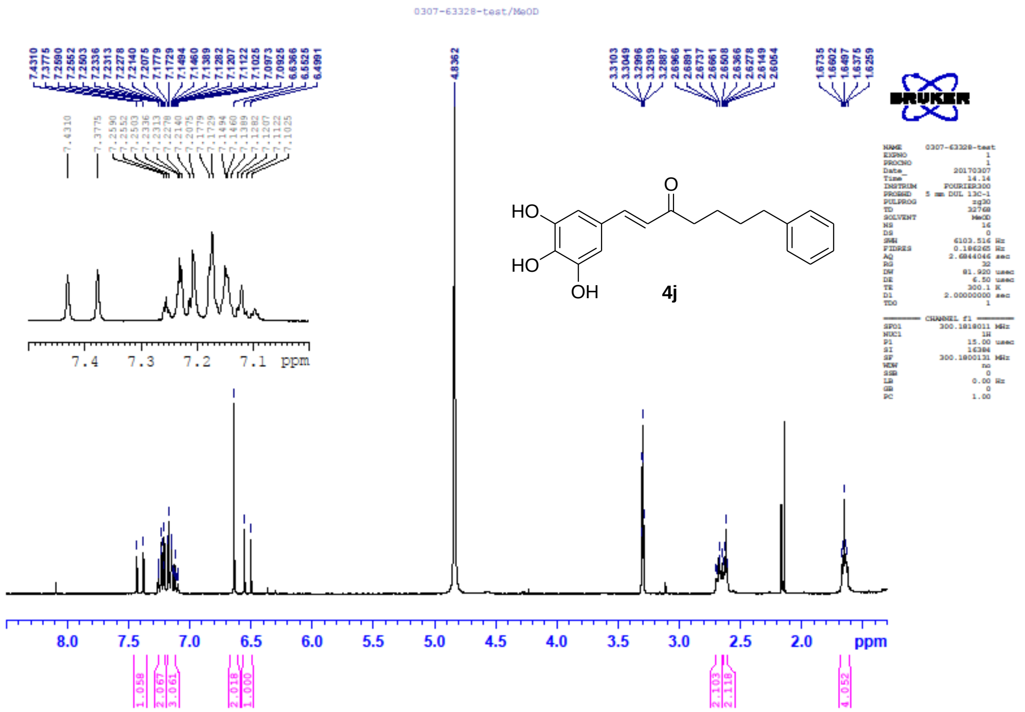

Supplementary Figure S21. ^{1}H NMR (MeOH-d_4, 300 MHz) spectrum of compound **4j**

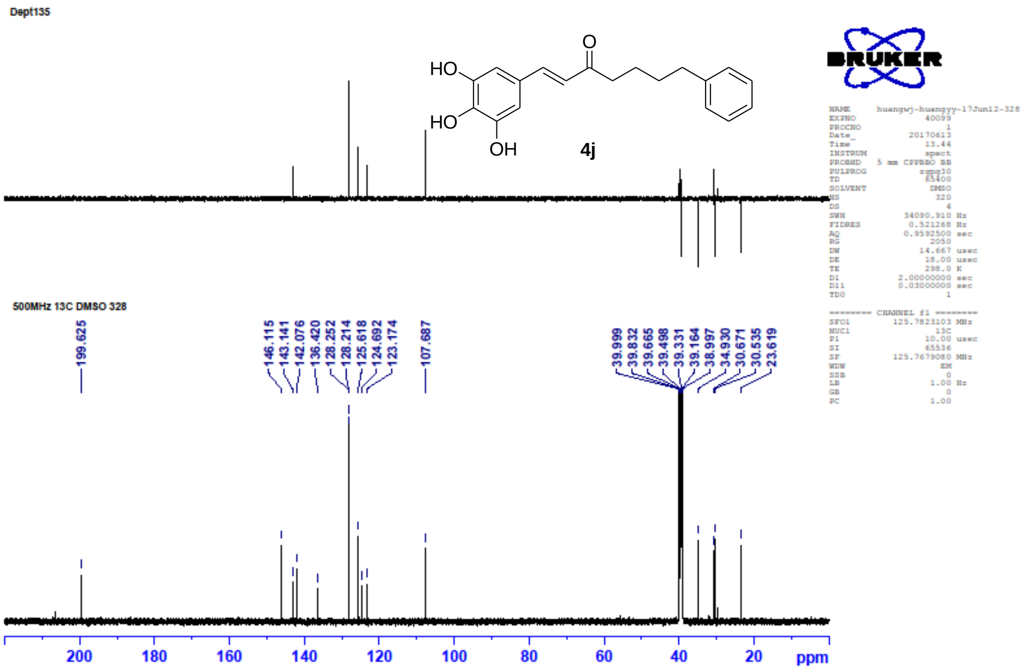

Supplementary Figure S22. ^{13}C NMR (DMSO-d_6, 125 MHz) spectrum of compound **4j**

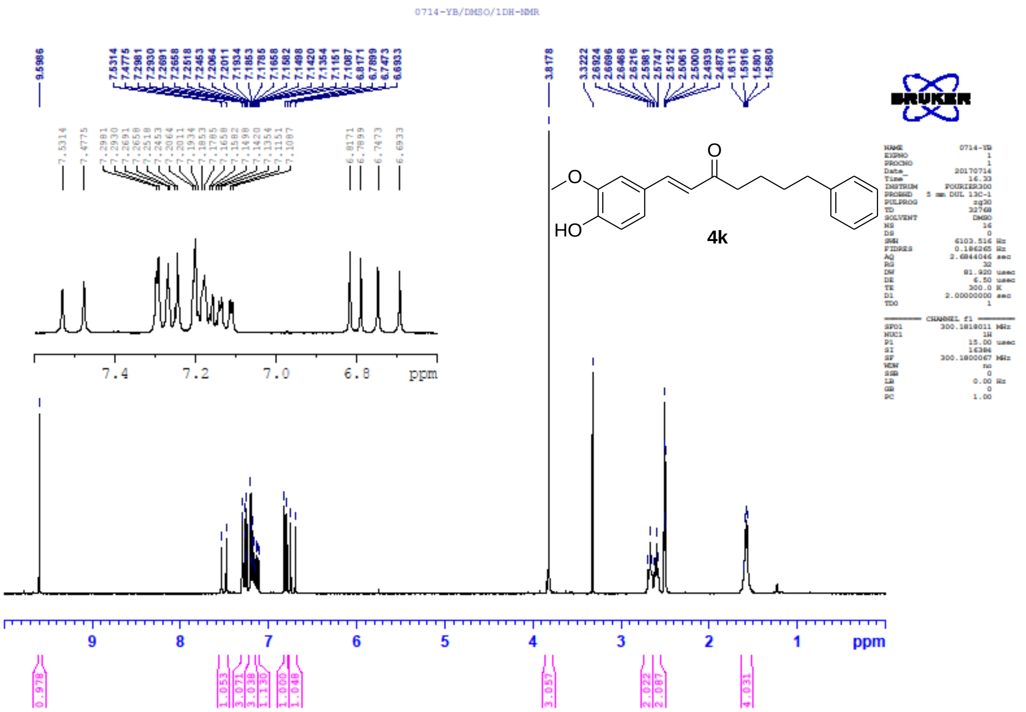

Supplementary Figure S23. ^{1}H NMR (DMSO-d_6, 300 MHz) spectrum of compound **4k**

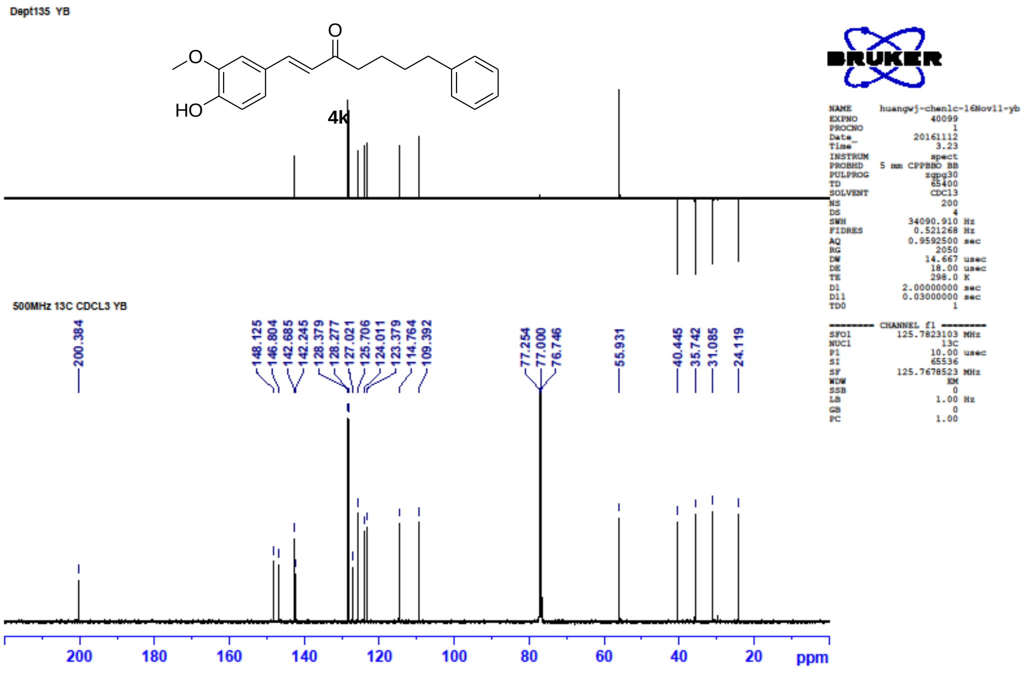

Supplementary Figure S24. ^{13}C NMR ($CDCl_3$, 125 MHz) spectrum of compound **4k**

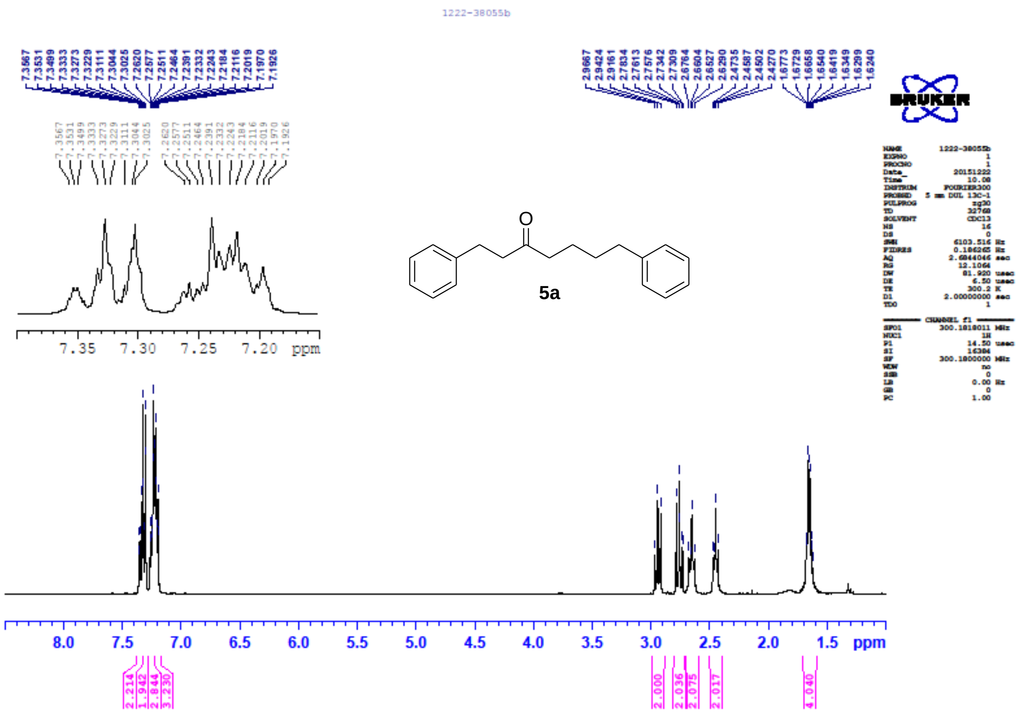

Supplementary Figure S25. ^{1}H NMR ($CDCl_3$, 300 MHz) spectrum of compound **5a**

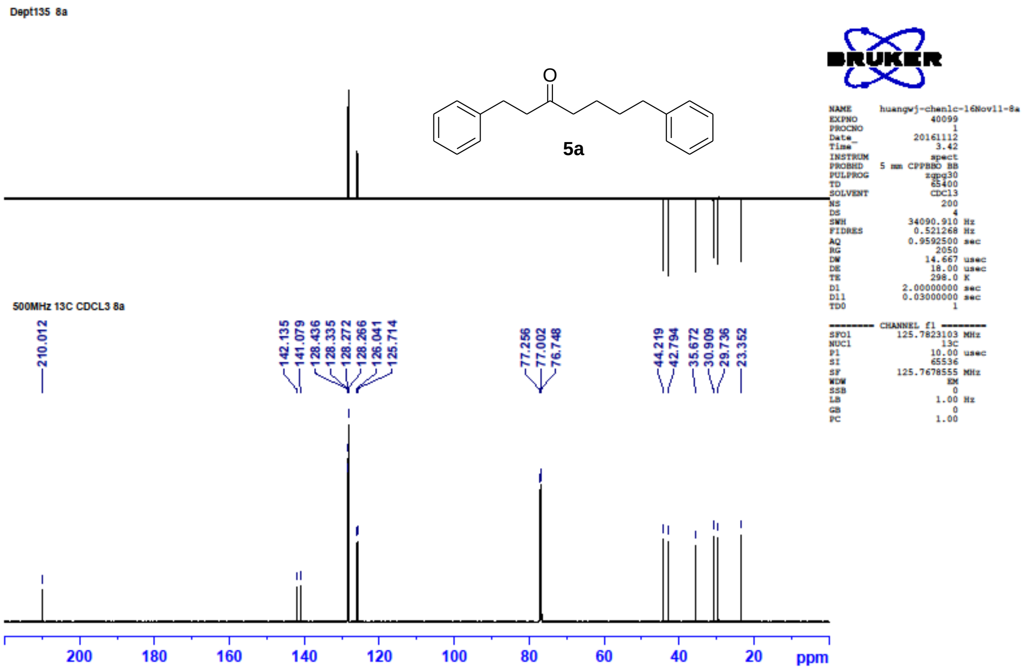

Supplementary Figure S26. ^{13}C NMR ($CDCl_3$, 125 MHz) spectrum of compound **5a**

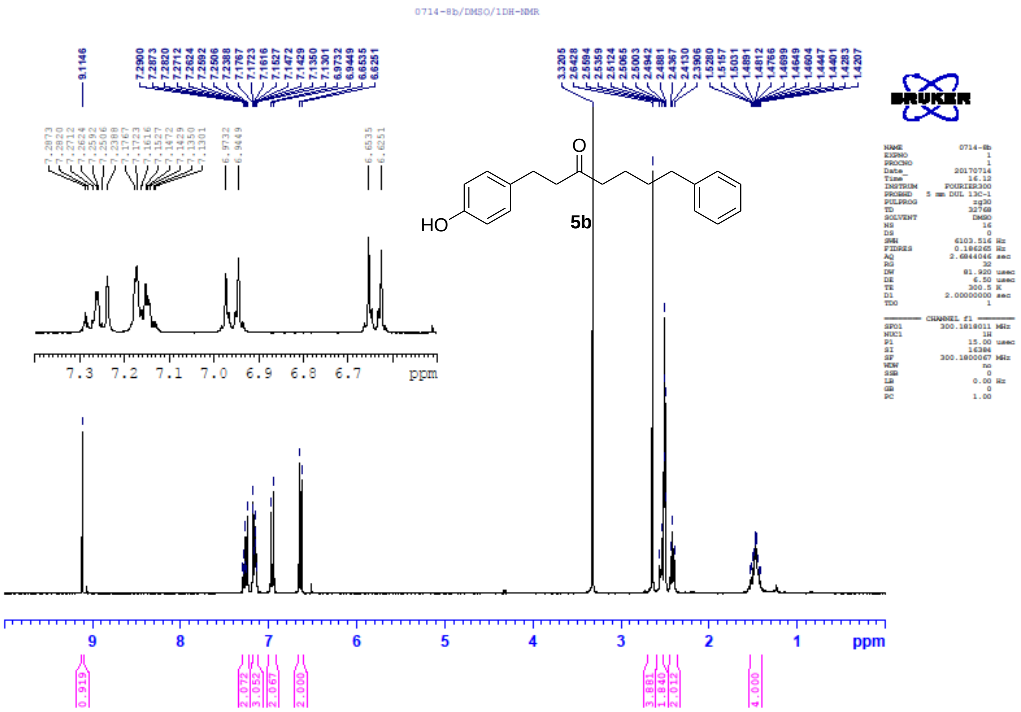

Supplementary Figure S27. ^{1}H NMR (DMSO-d_6, 300 MHz) spectrum of compound **5b**

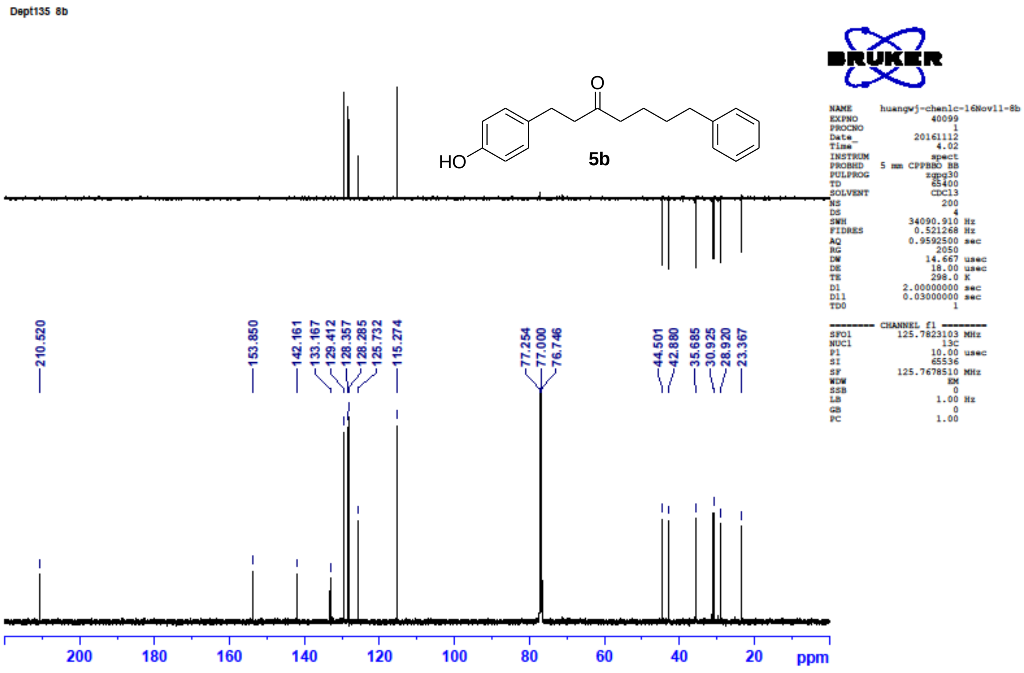

Supplementary Figure S28. ^{13}C NMR ($CDCl_3$, 125 MHz) spectrum of compound **5b**

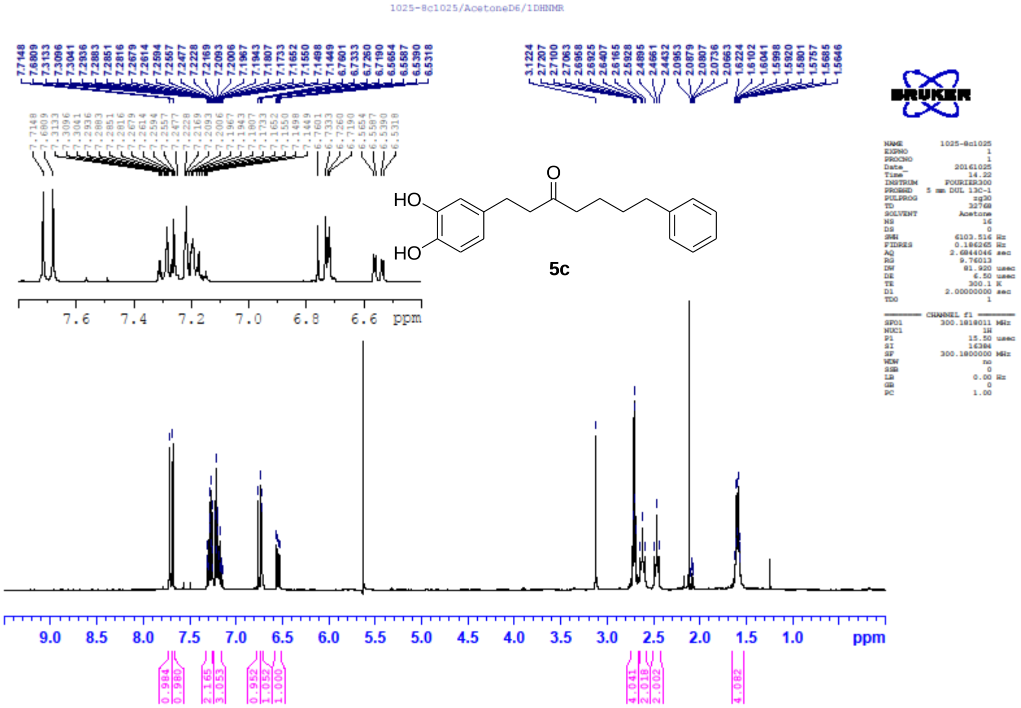

Supplementary Figure S29. ^{1}H NMR (Acetone-d_6, 300 MHz) spectrum of compound **5c**

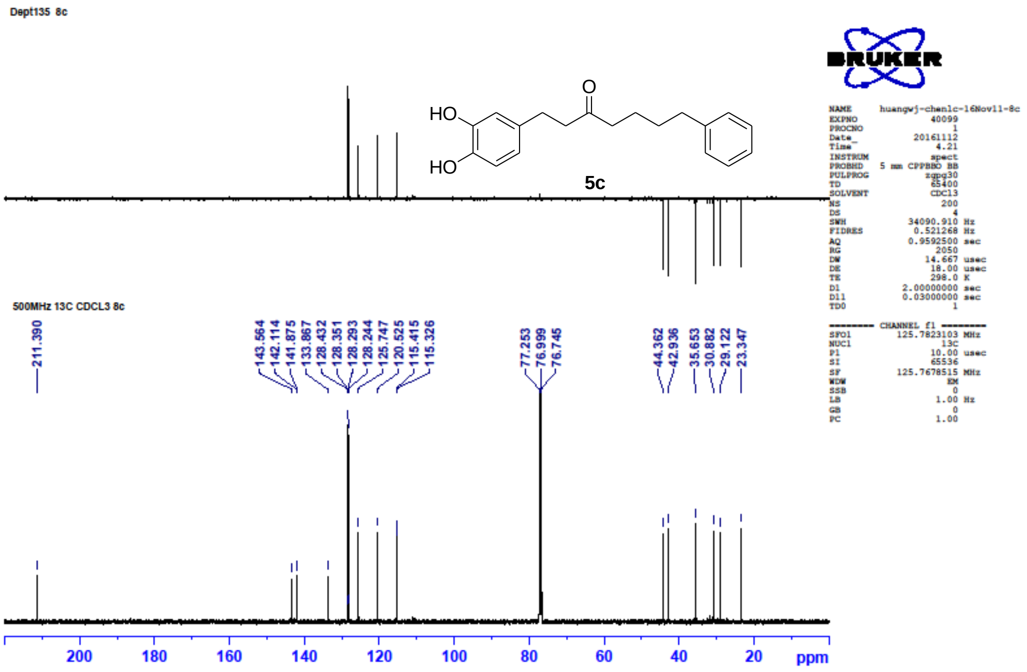

Supplementary Figure S30. ^{13}C NMR ($CDCl_3$, 125 MHz) spectrum of compound **5c**

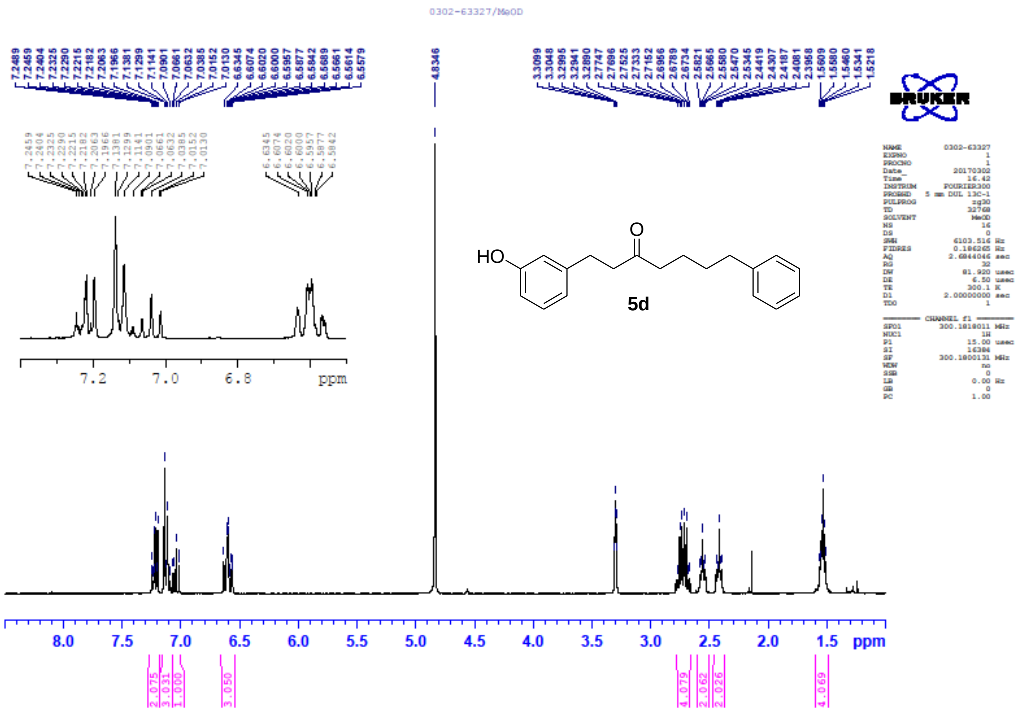

Supplementary Figure S31. ^{1}H NMR (MeOH-d_4, 300 MHz) spectrum of compound **5d**

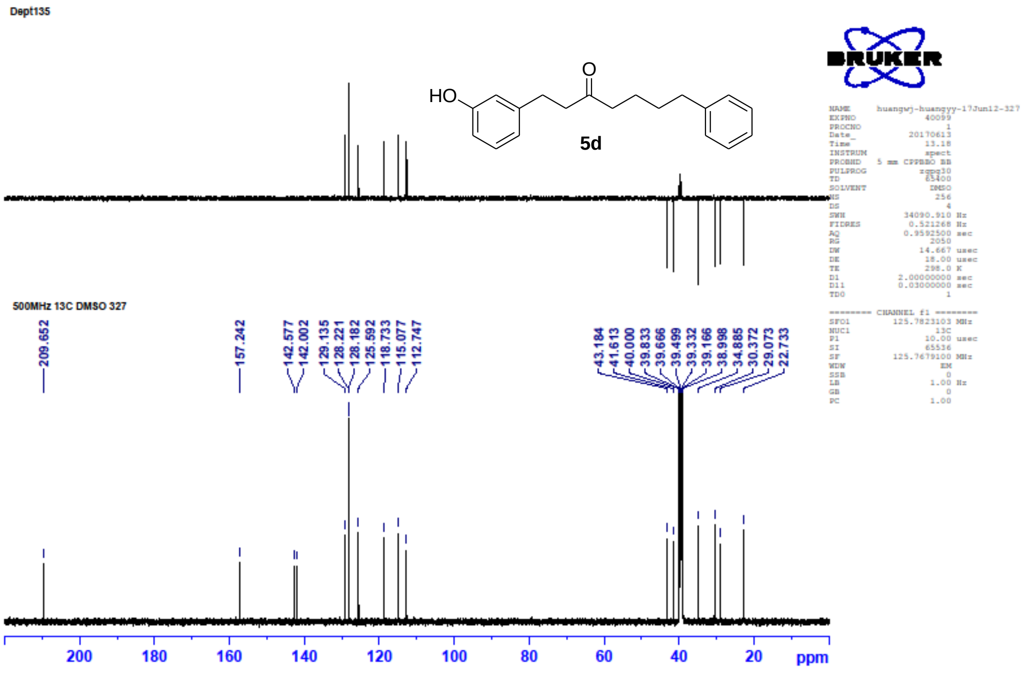

Supplementary Figure S32. ^{13}C NMR (DMSO-d_6, 125 MHz) spectrum of compound **5d**

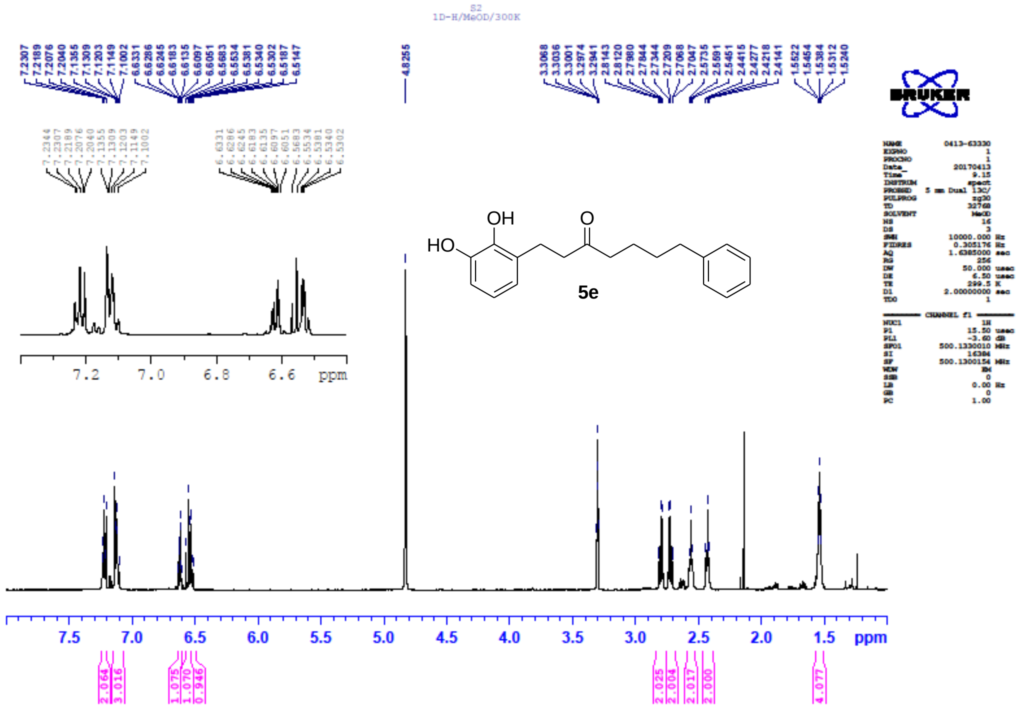

Supplementary Figure S33. ^{1}H NMR (MeOH-d_4, 300 MHz) spectrum of compound **5e**

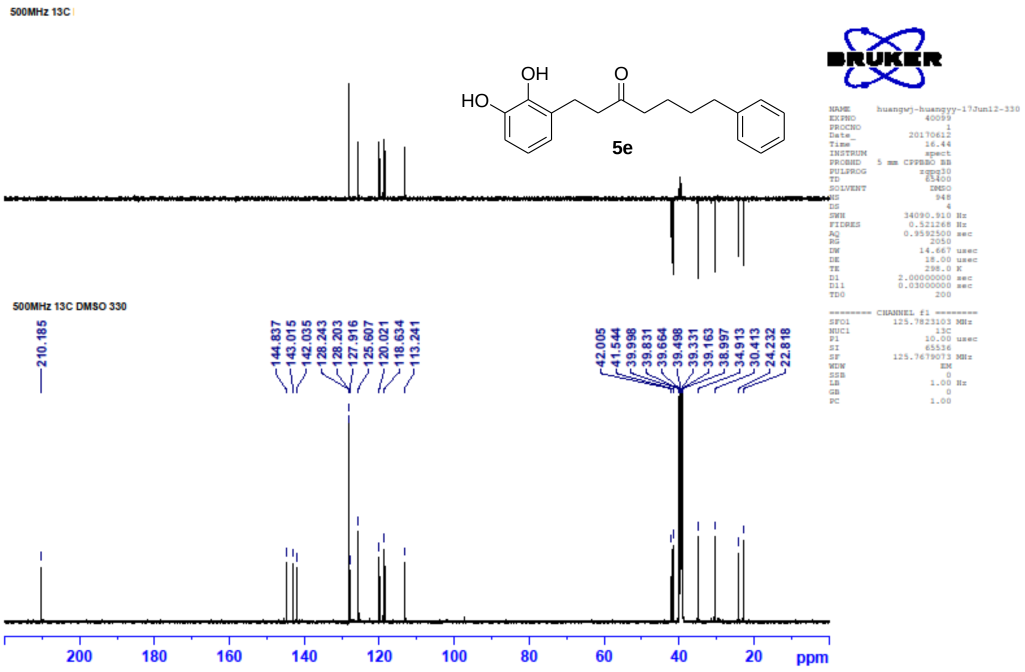

Supplementary Figure S34. ^{13}C NMR (DMSO-d_6, 125 MHz) spectrum of compound **5e**

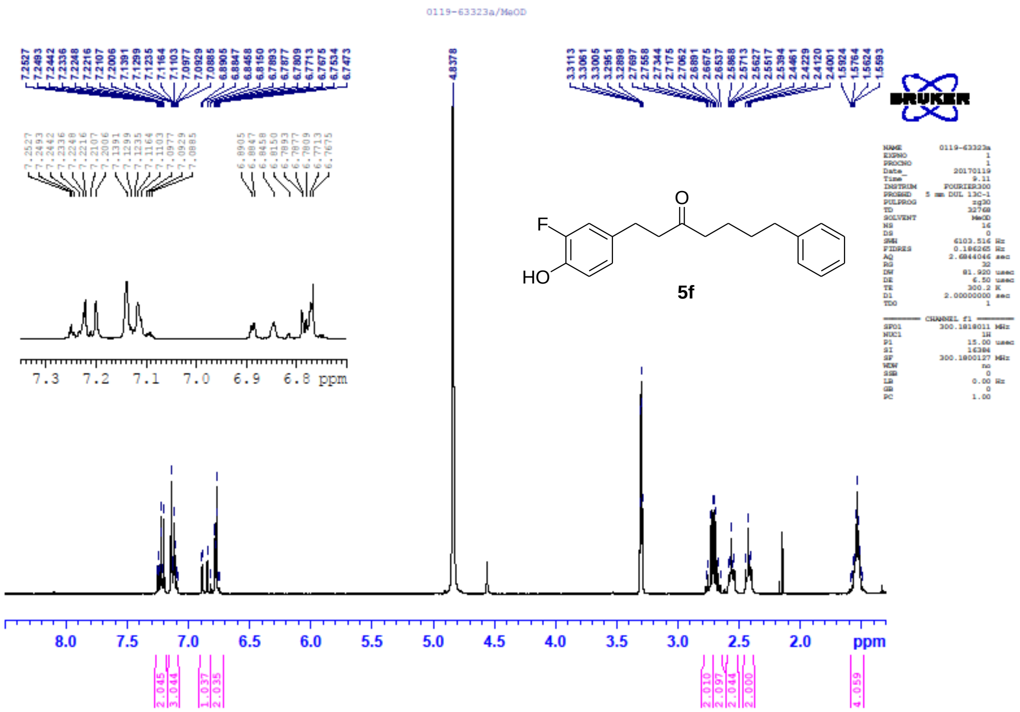

Supplementary Figure S35. ^{1}H NMR (MeOH-d_4, 300 MHz) spectrum of compound **5f**

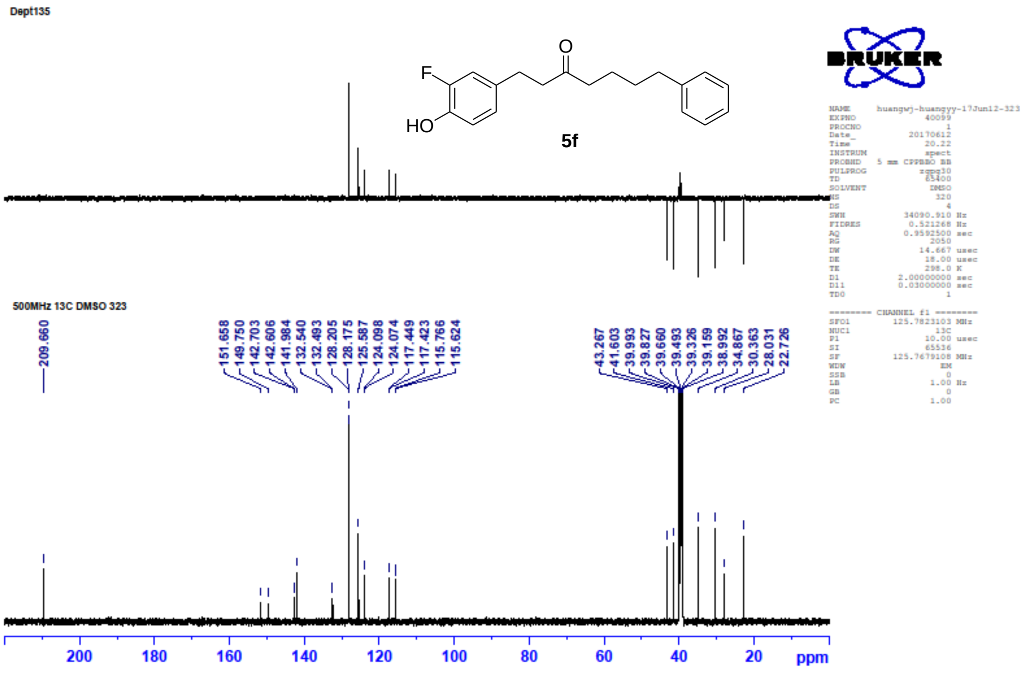

Supplementary Figure S36. ^{13}C NMR (DMSO-d_6, 125 MHz) spectrum of compound **5f**

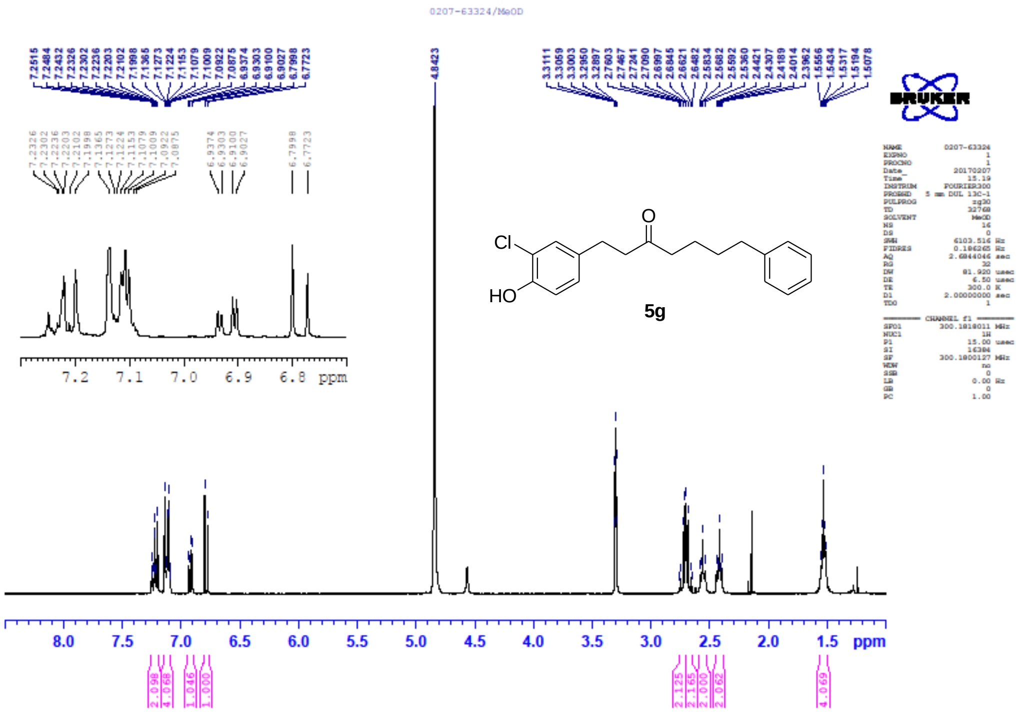

Supplementary Figure S37. ^{1}H NMR (MeOH-d_4, 300 MHz) spectrum of compound **5g**

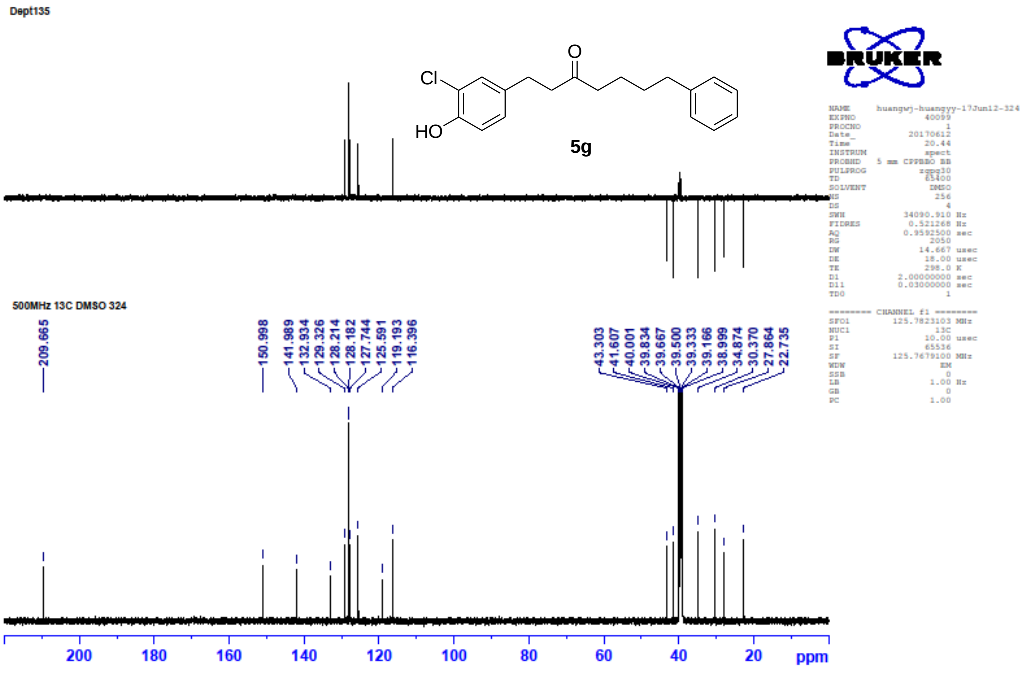

Supplementary Figure S38. ^{13}C NMR (DMSO-d_6, 125 MHz) spectrum of compound **5g**

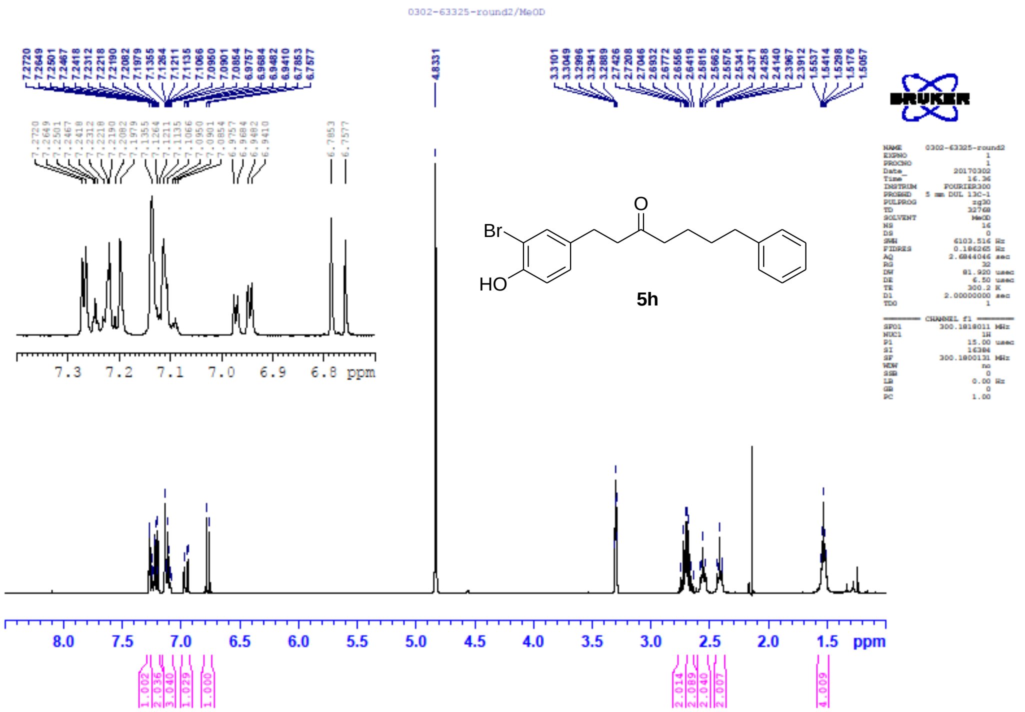

Supplementary Figure S39. ^{1}H NMR (MeOH-d_4, 300 MHz) spectrum of compound **5h**

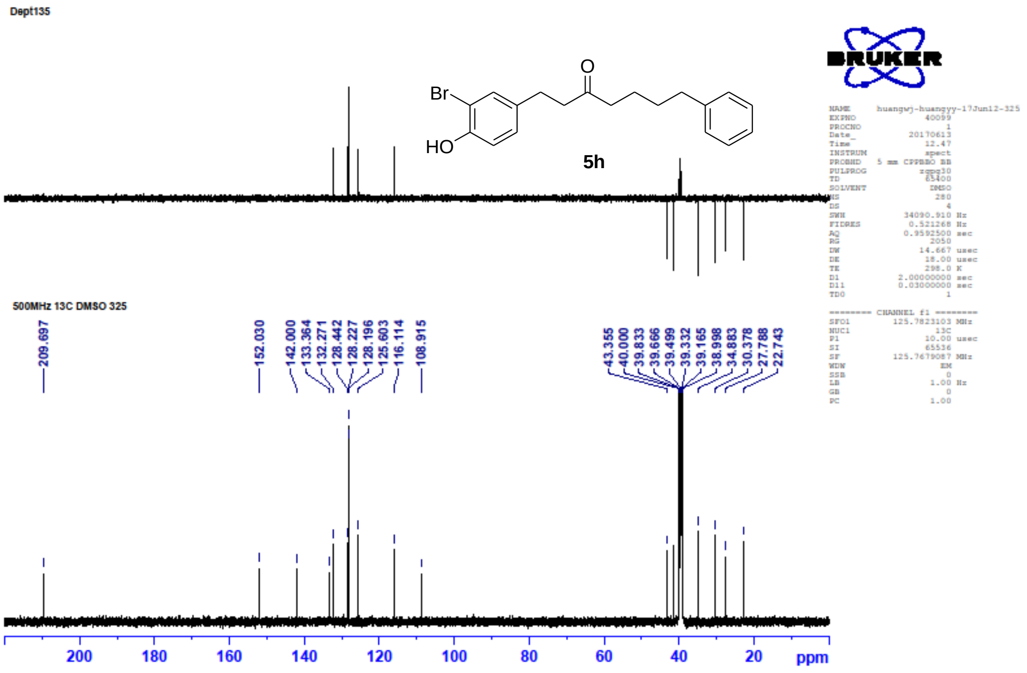

Supplementary Figure S40. ^{13}C NMR (DMSO-d_6, 125 MHz) spectrum of compound **5h**

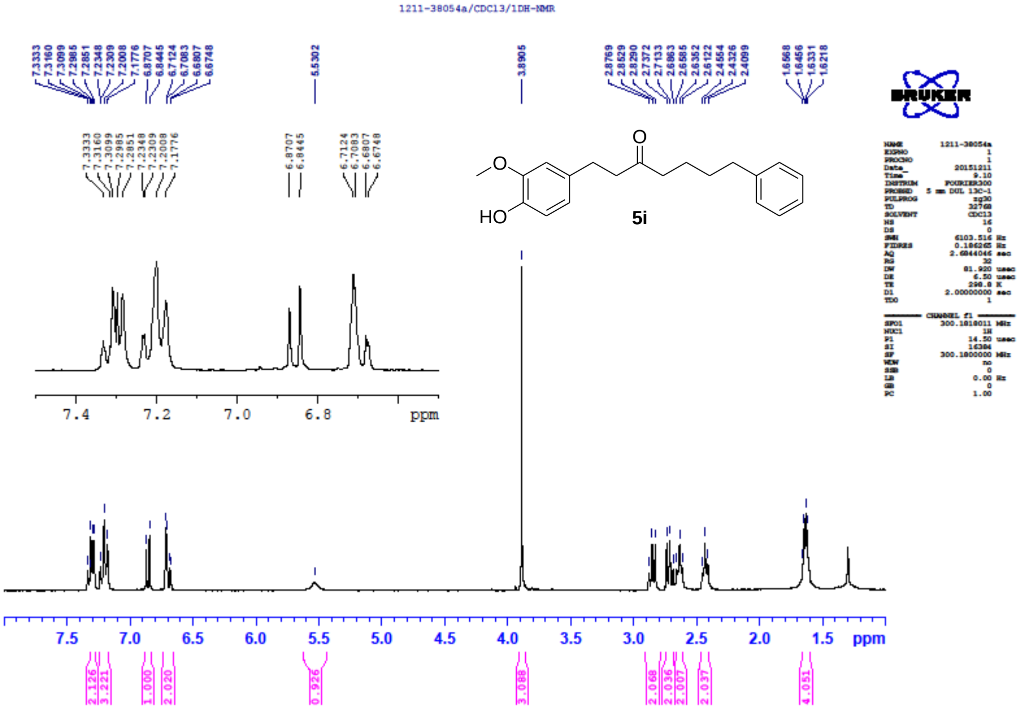

Supplementary Figure S41. ^{1}H NMR ($CDCl_3$, 300 MHz) spectrum of compound **5i**

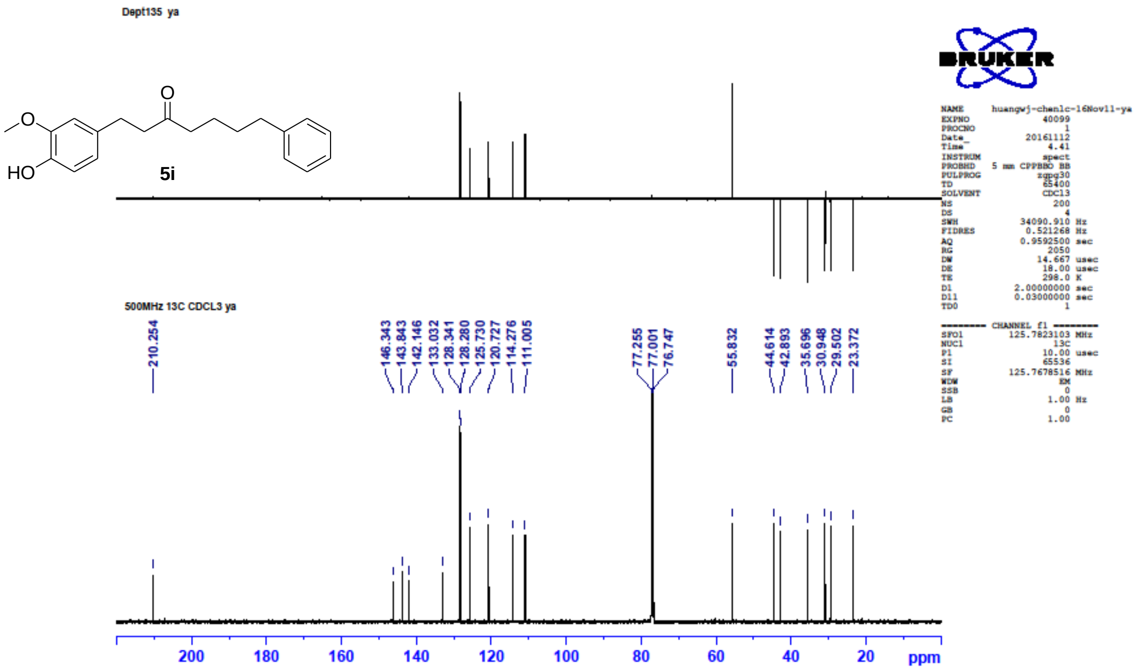

Supplementary Figure S42. ^{13}C NMR ($CDCl_3$, 125 MHz) spectrum of compound **5i**

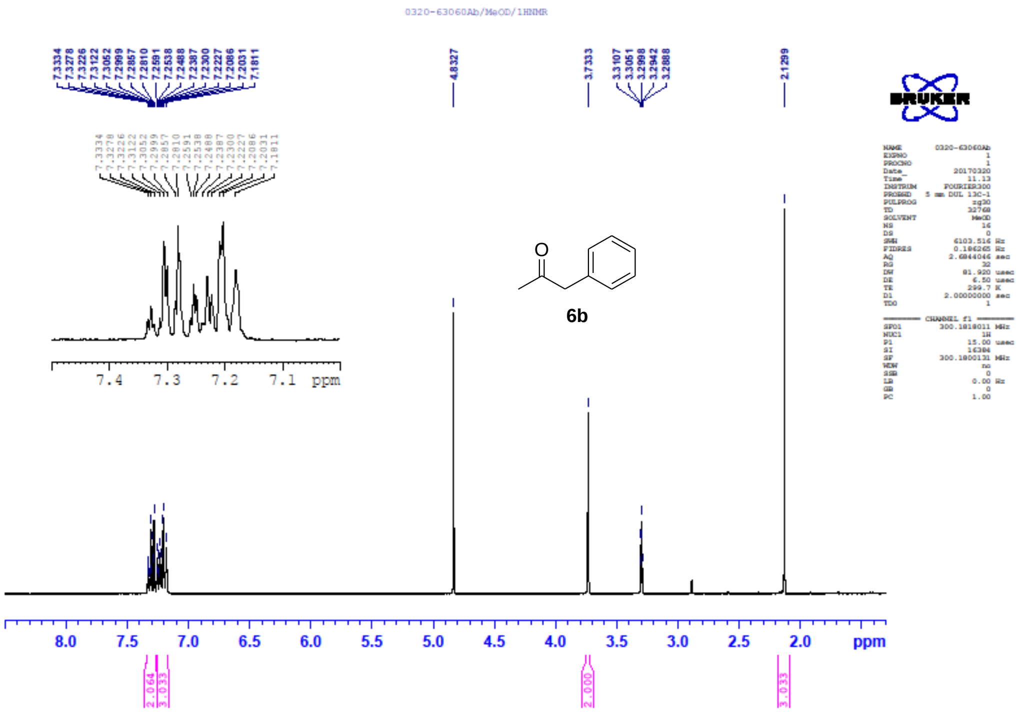

Supplementary Figure S43. ^{1}H NMR (MeOH-d_4, 300 MHz) spectrum of compound **6b**

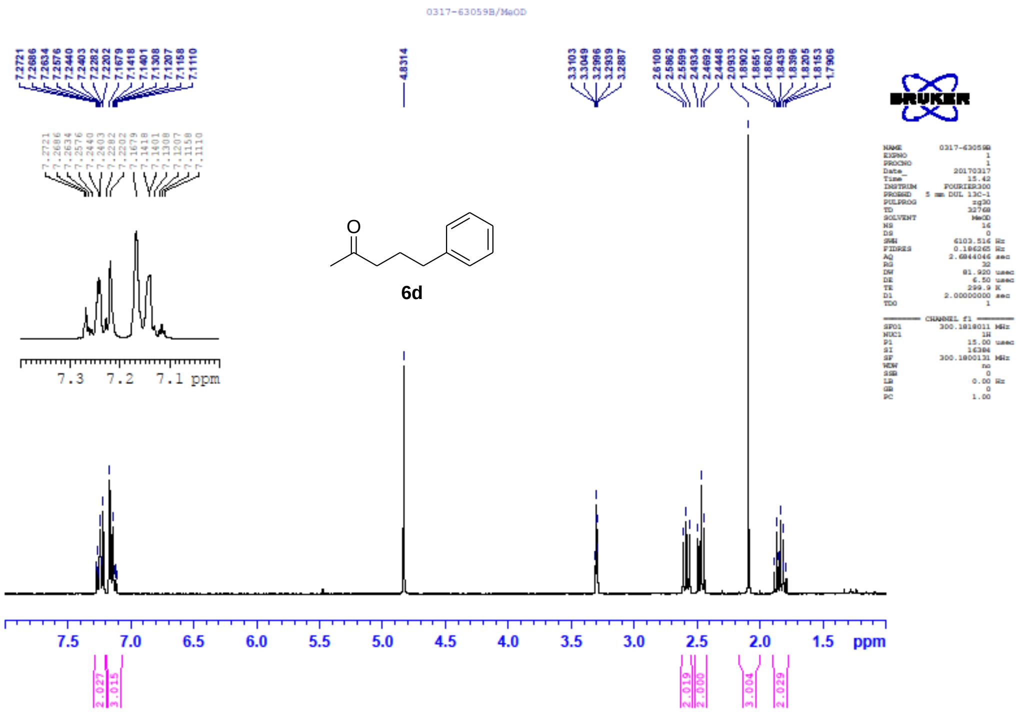

Supplementary Figure S44. ^{1}H NMR (MeOH-d_4, 300 MHz) spectrum of compound **6d**

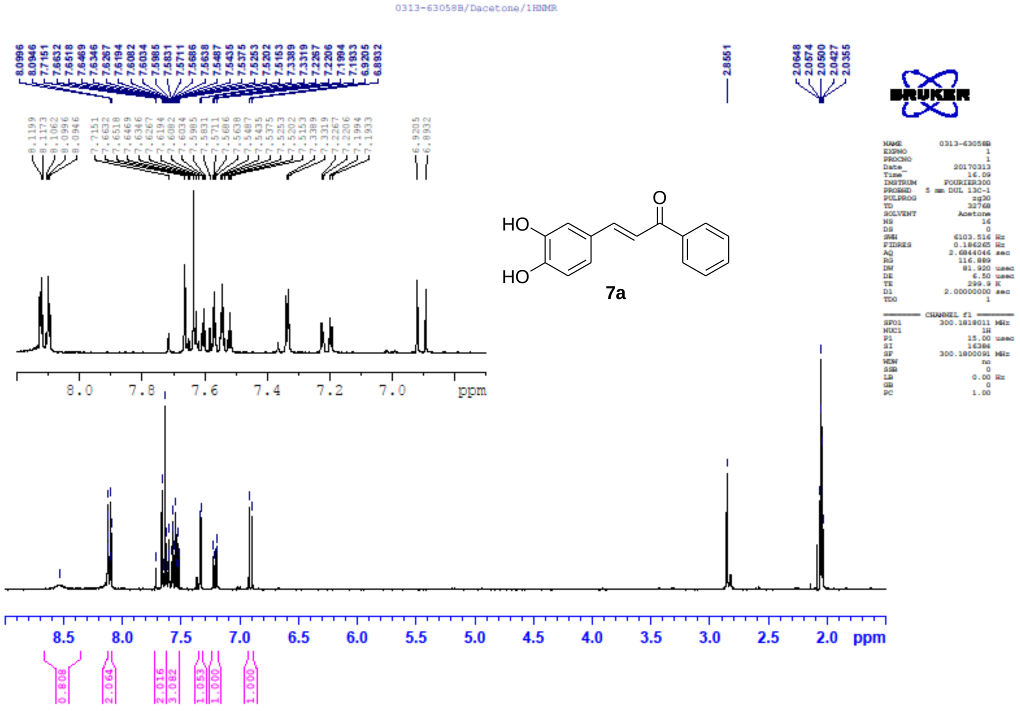

Supplementary Figure S45. ^{1}H NMR (Acetone-d_6, 300 MHz) spectrum of compound **7a**

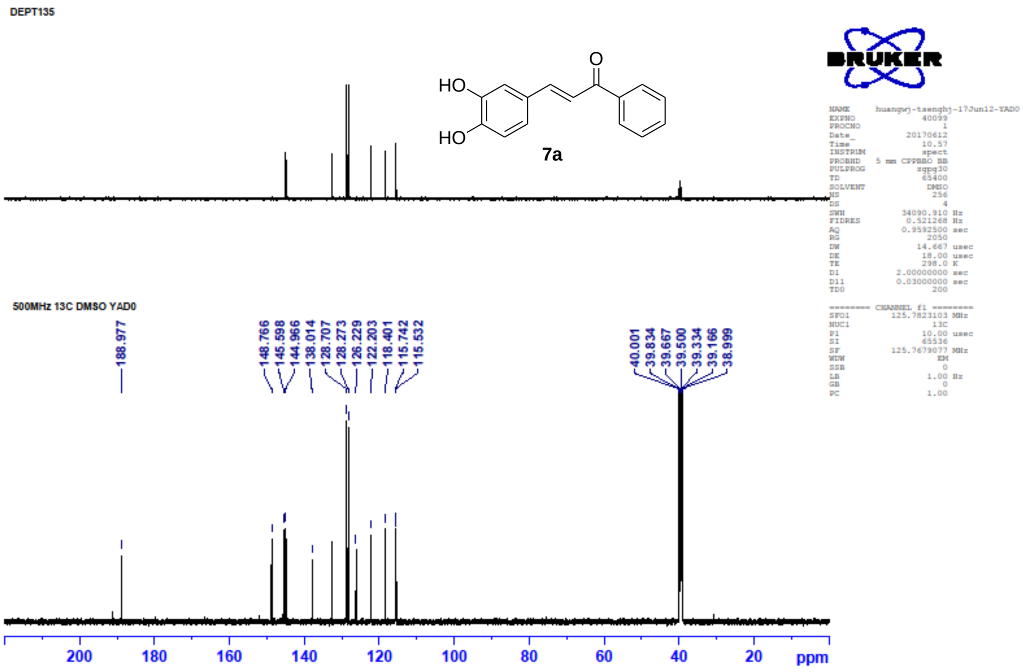

Supplementary Figure S46. ^{13}C NMR (DMSO-d_6, 125 MHz) spectrum of compound **7a**

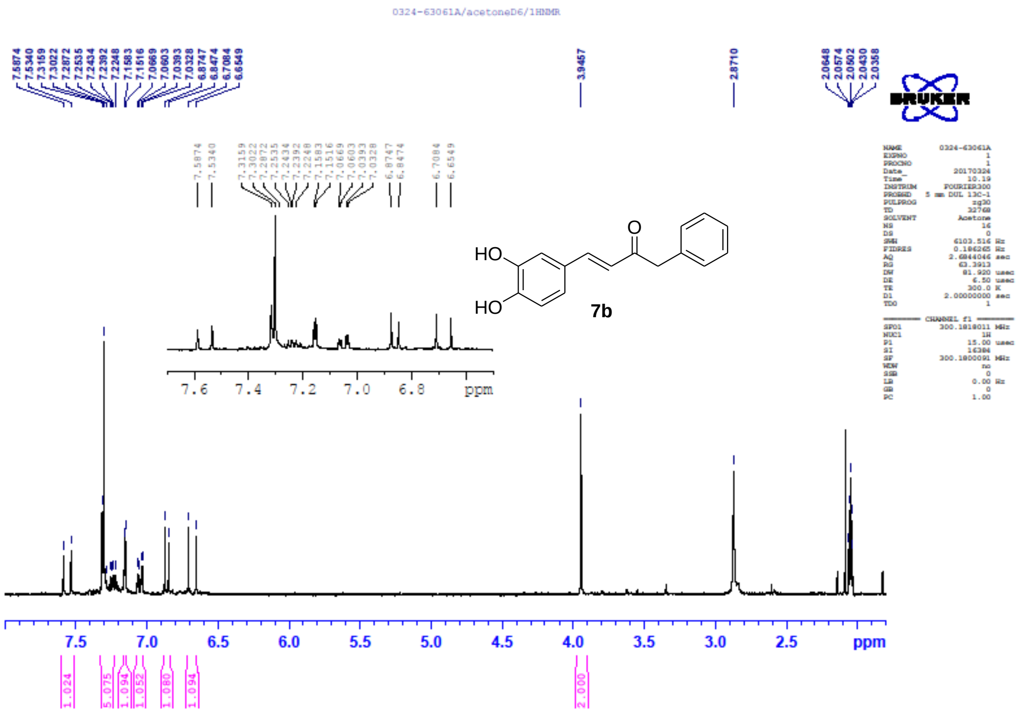

Supplementary Figure S47. ^{1}H NMR (Acetone-d_6, 300 MHz) spectrum of compound **7b**

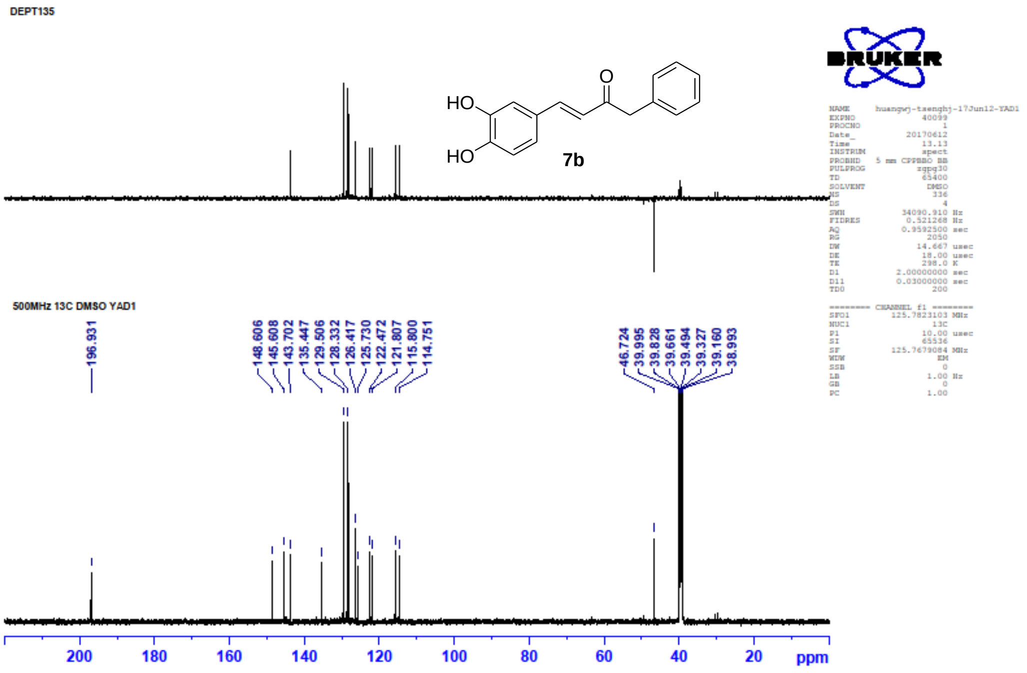

Supplementary Figure S48. ^{13}C NMR (DMSO-d_6, 125 MHz) spectrum of compound **7b**

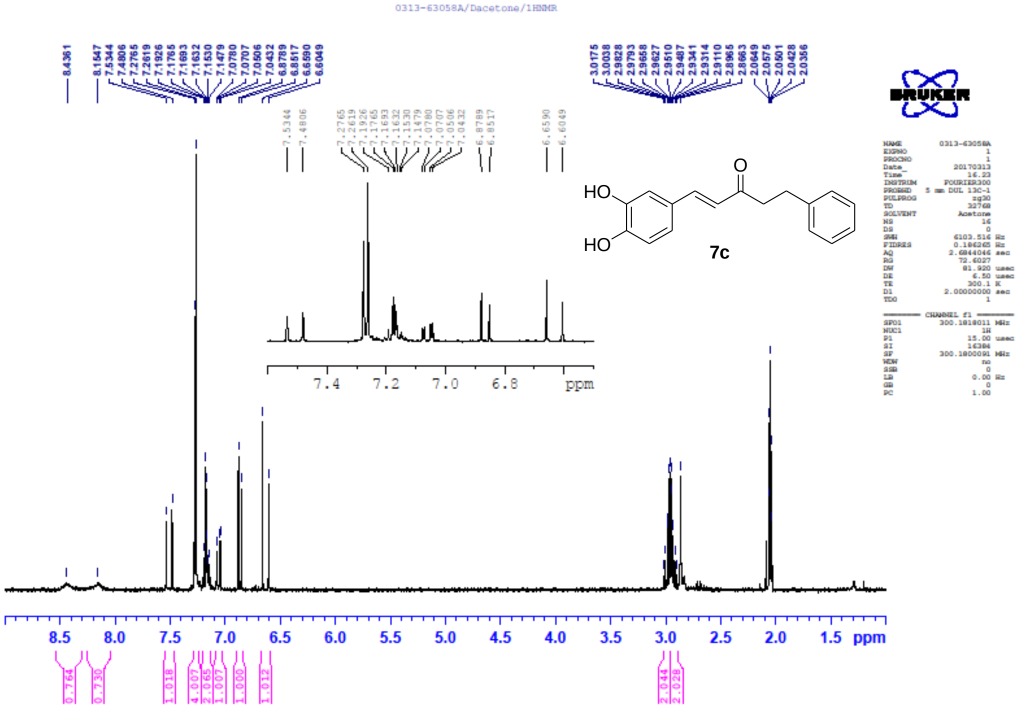

Supplementary Figure S49. ^{1}H NMR (Acetone-d_6, 300 MHz) spectrum of compound **7c**

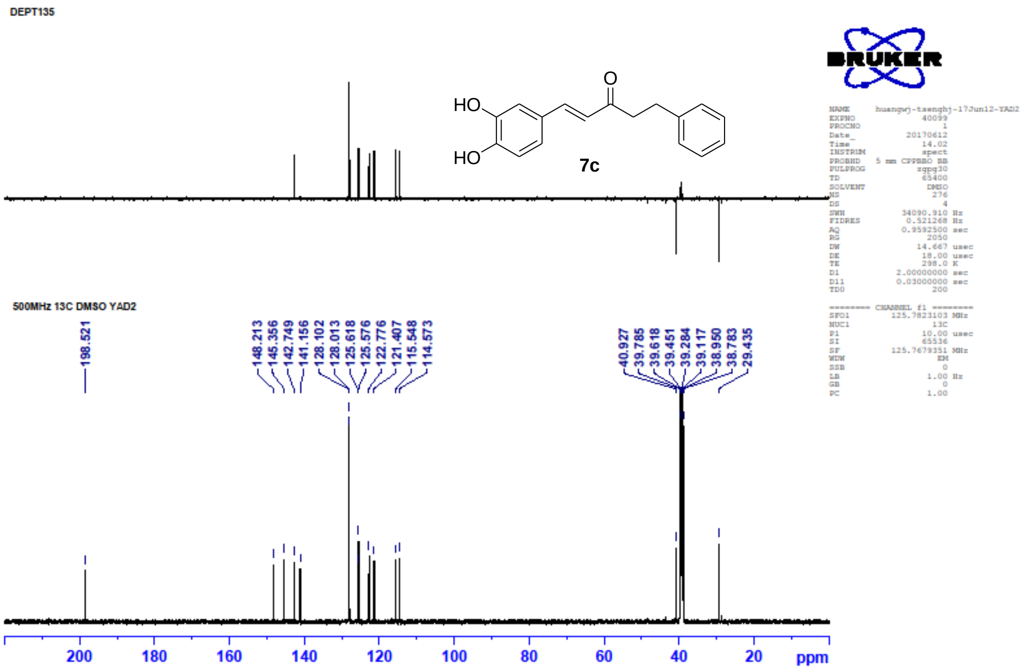

Supplementary Figure S50. ^{13}C NMR (DMSO-d_6, 125 MHz) spectrum of compound **7c**

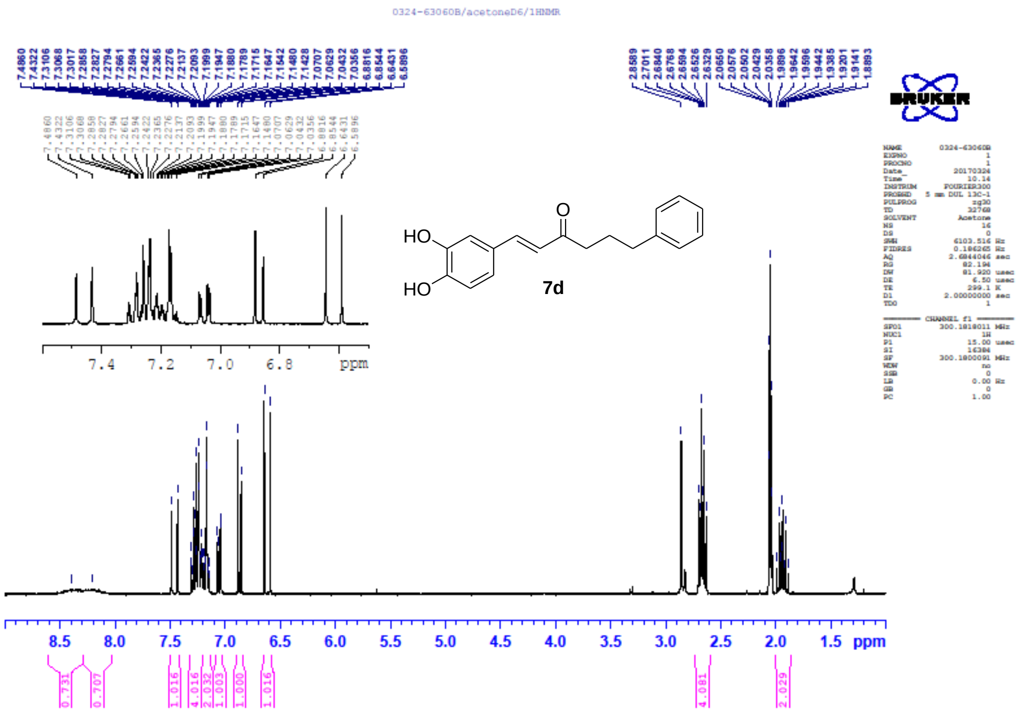

Supplementary Figure S51. ^{1}H NMR (Acetone-d_6, 300 MHz) spectrum of compound **7d**

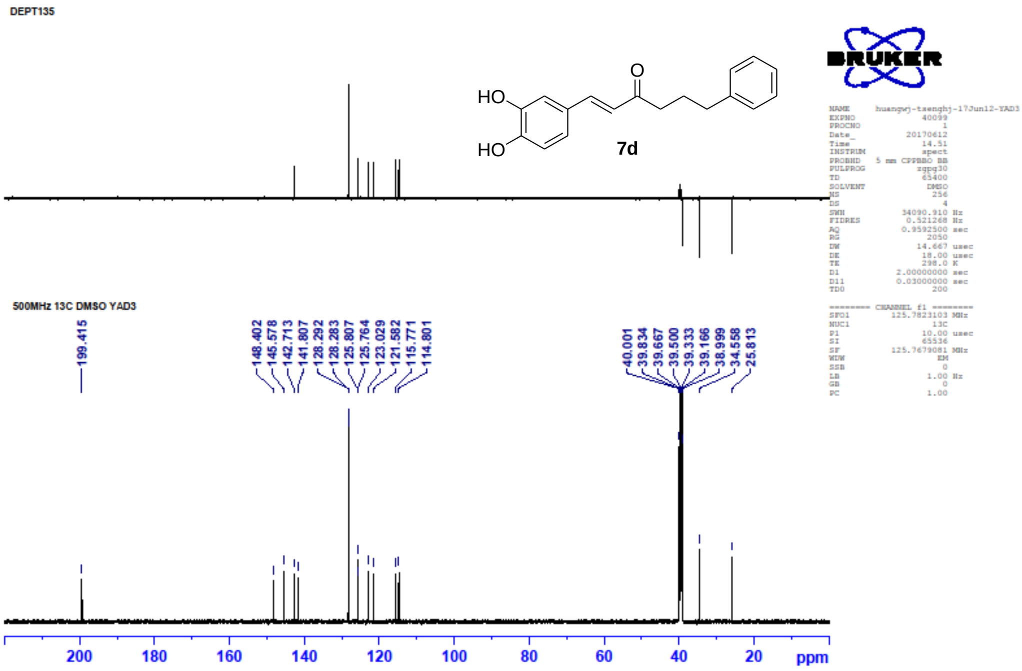

Supplementary Figure S52. ^{13}C NMR (DMSO-d_6, 125 MHz) spectrum of compound **7d**

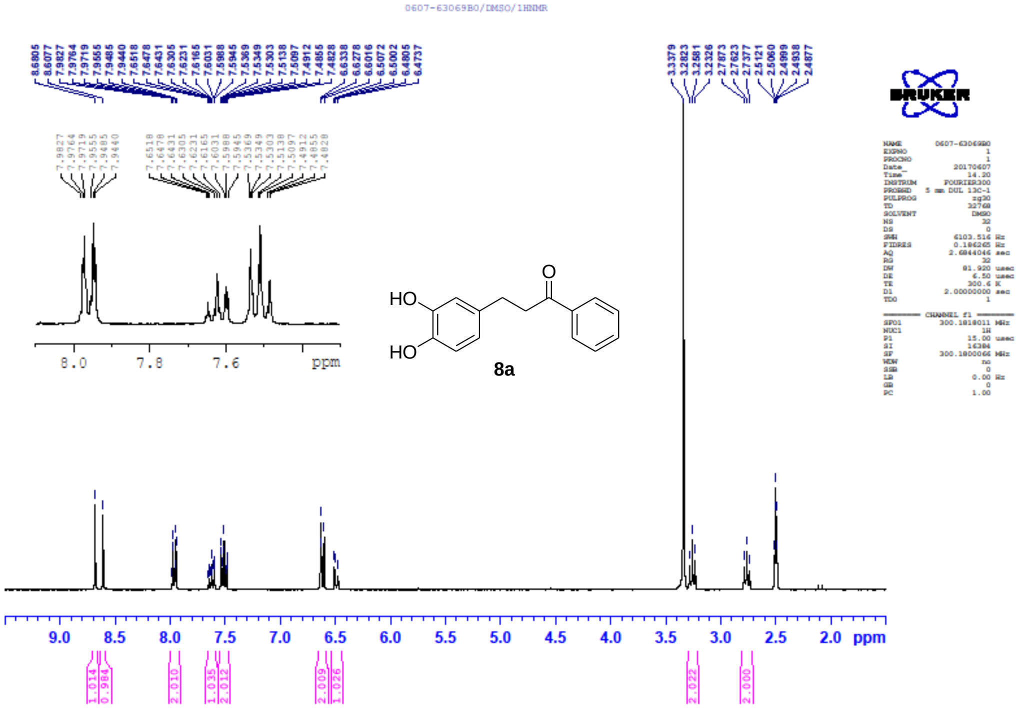

Supplementary Figure S53. ^{1}H NMR (DMSO-d_6, 300 MHz) spectrum of compound **8a**

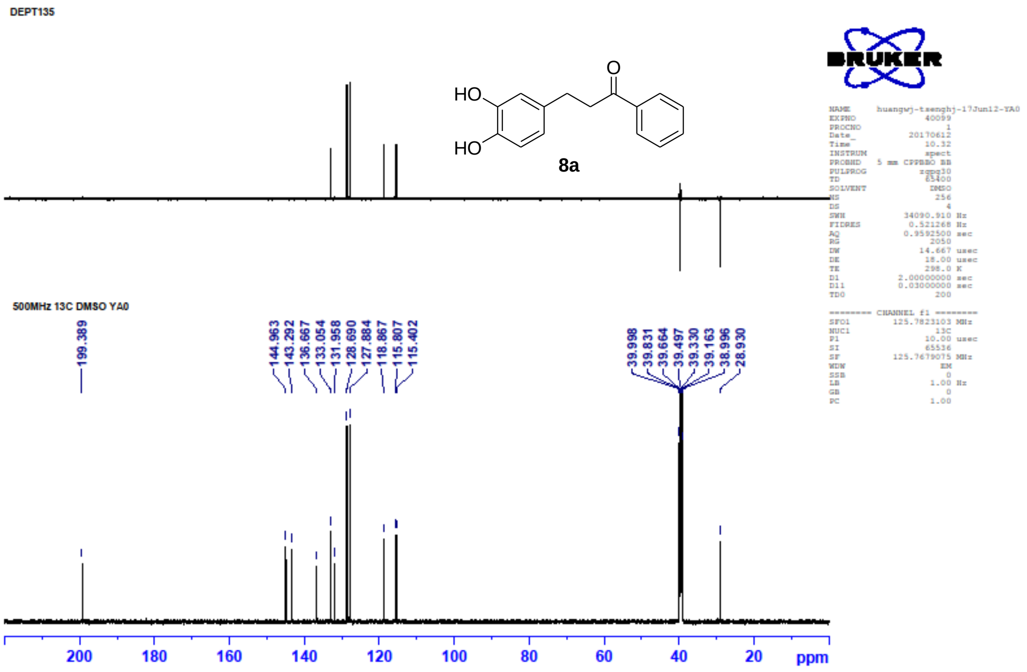

Supplementary Figure S54. ^{13}C NMR (DMSO-d_6, 125 MHz) spectrum of compound **8a**

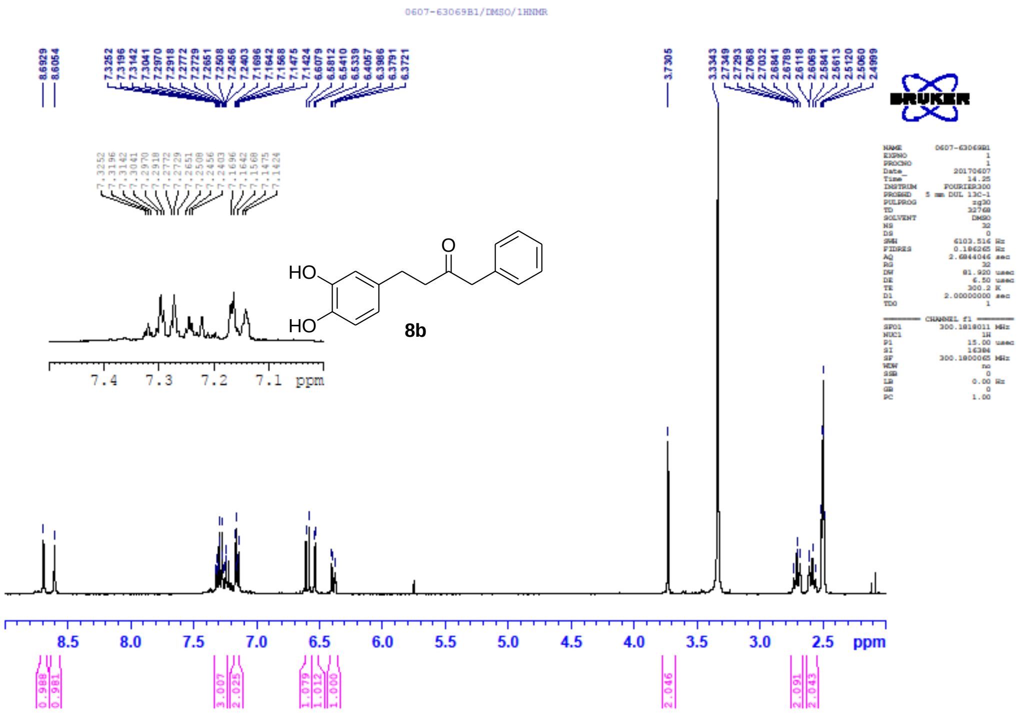

Supplementary Figure S55. ^{1}H NMR (DMSO-d_6, 300 MHz) spectrum of compound **8b**

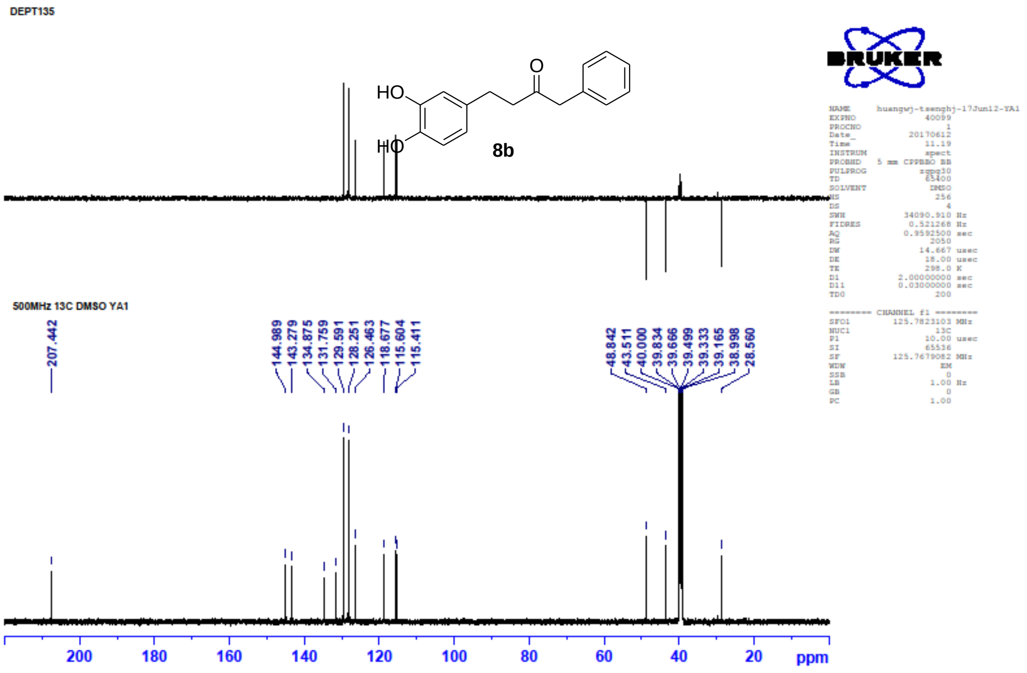

Supplementary Figure S56. ^{13}C NMR (DMSO-d_6, 125 MHz) spectrum of compound **8b**

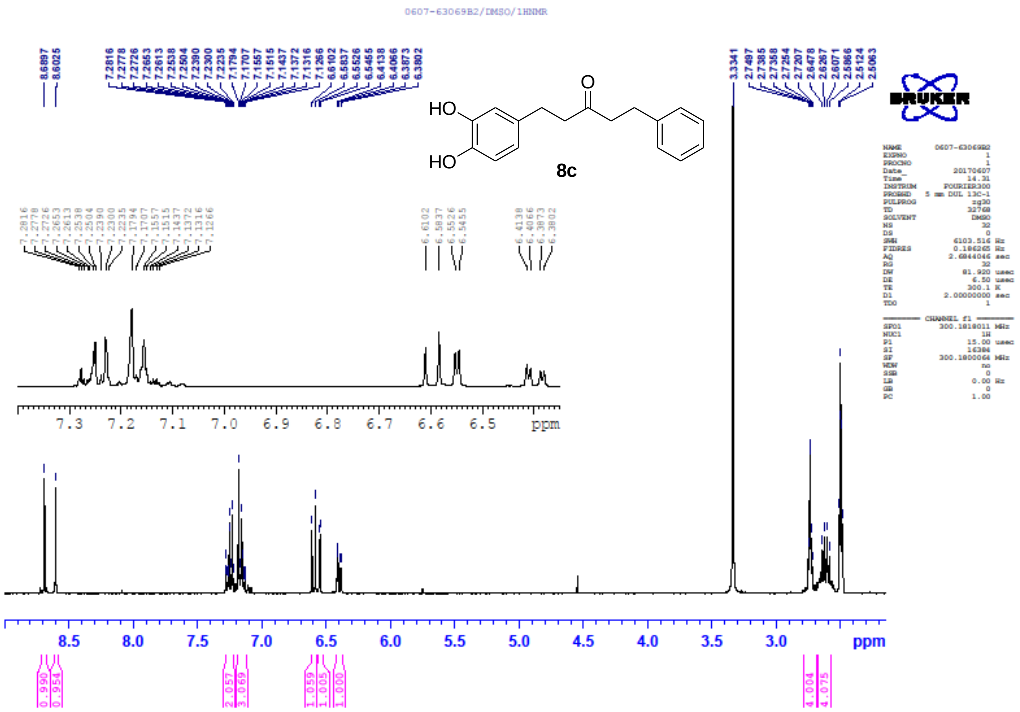

Supplementary Figure S57 ^{1}H NMR (DMSO-d_6, 300 MHz) spectrum of compound **8c**

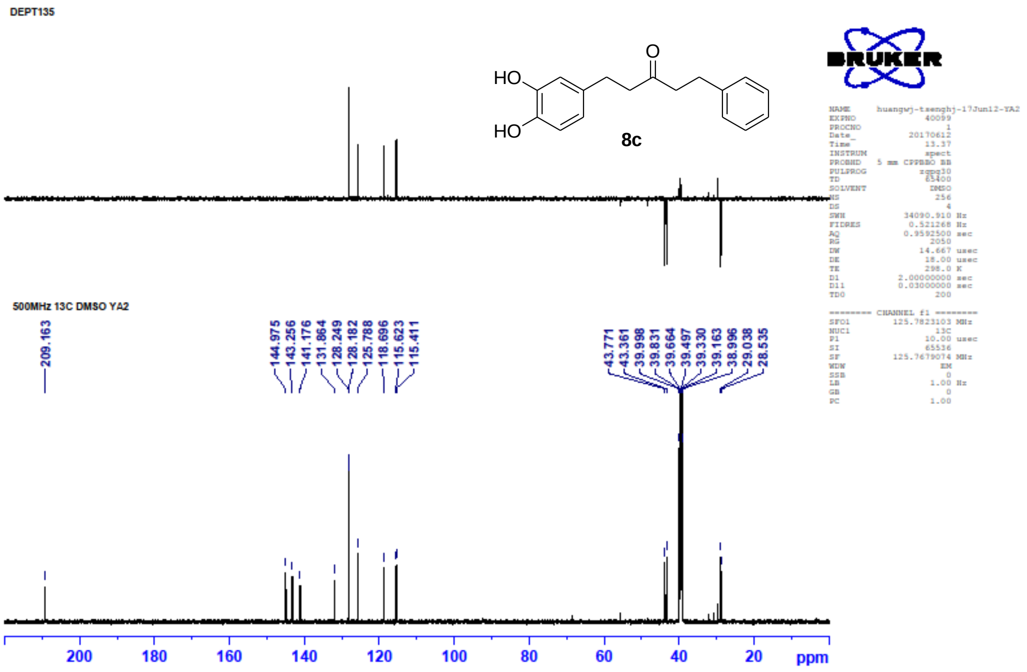

Supplementary Figure S58. ^{13}C NMR (DMSO-d_6, 125 MHz) spectrum of compound **8c**

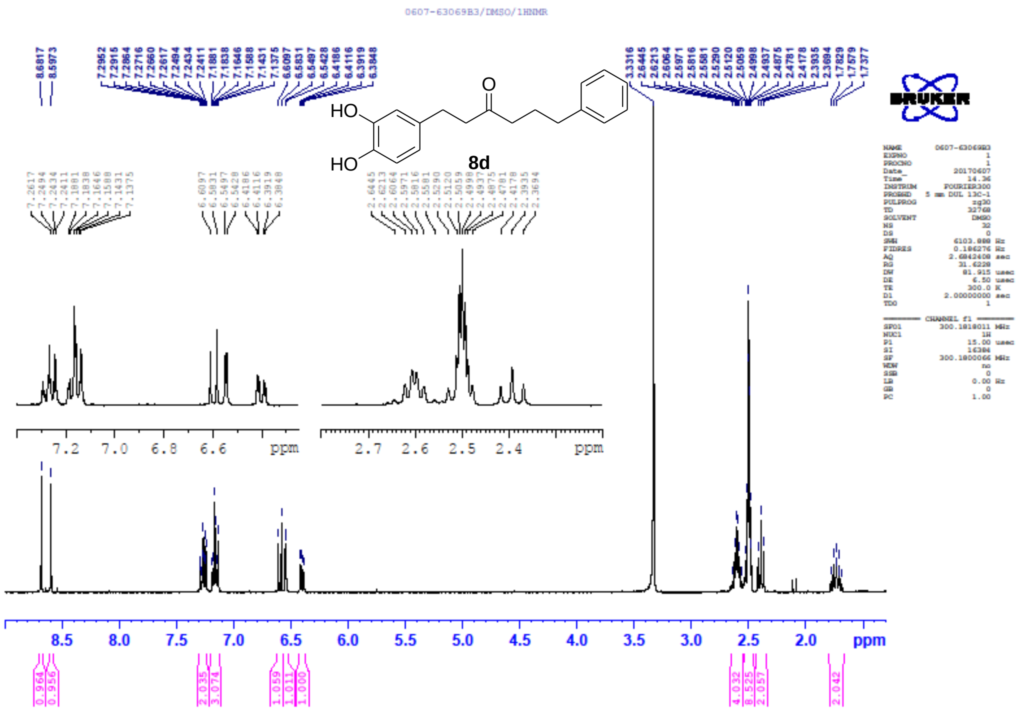

Supplementary Figure S59. ^{1}H NMR (DMSO-d_6, 300 MHz) spectrum of compound **8d**

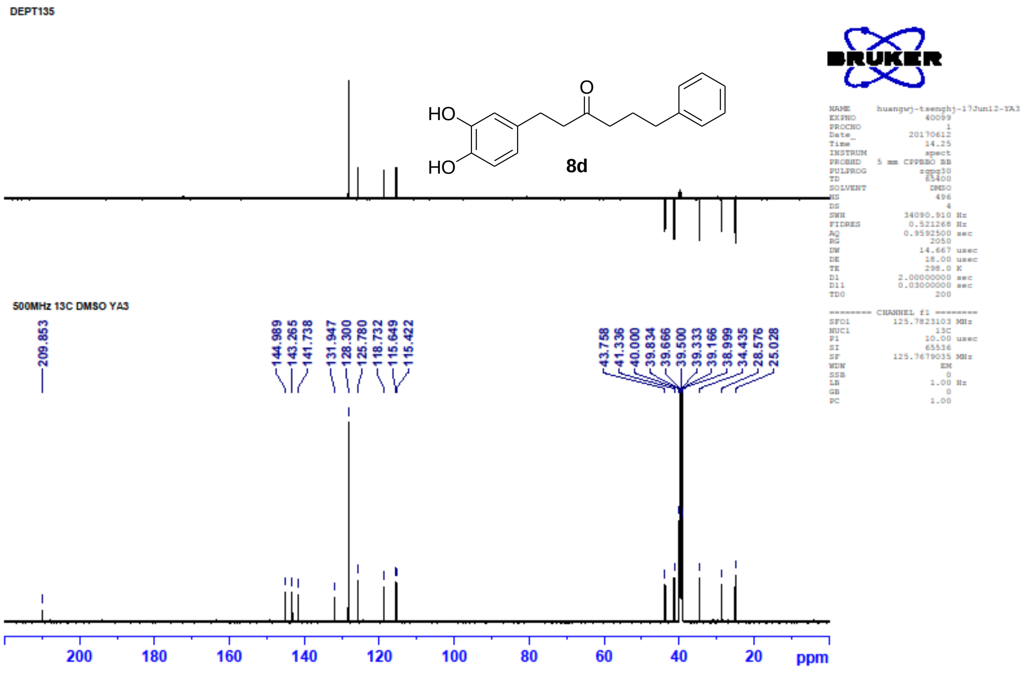

Supplementary Figure S60. ^{13}C NMR (DMSO-d_6, 125 MHz) spectrum of compound **8d**

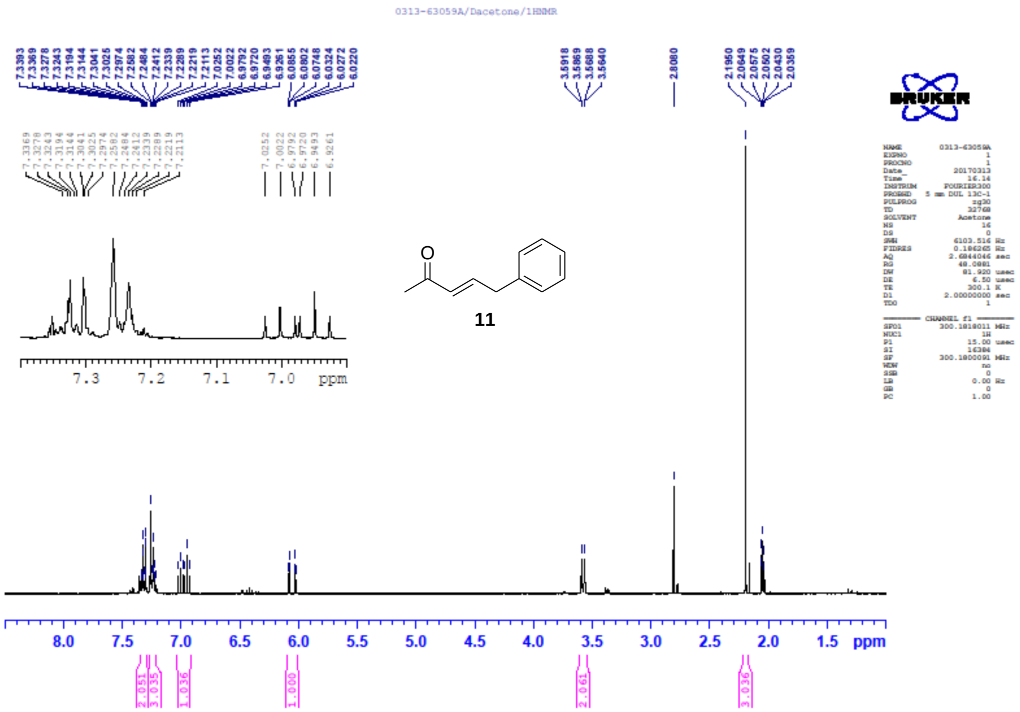

Supplementary Figure S61. ^{1}H NMR (Acetone-d_6, 300 MHz) spectrum of compound **11**

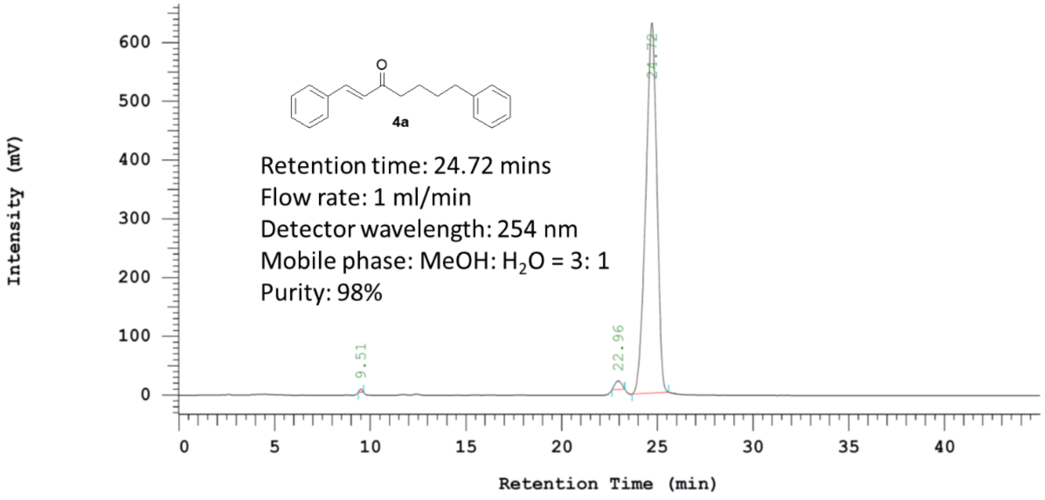

Supplementary Figure S62. HPLC chromatogram of compound **4a**

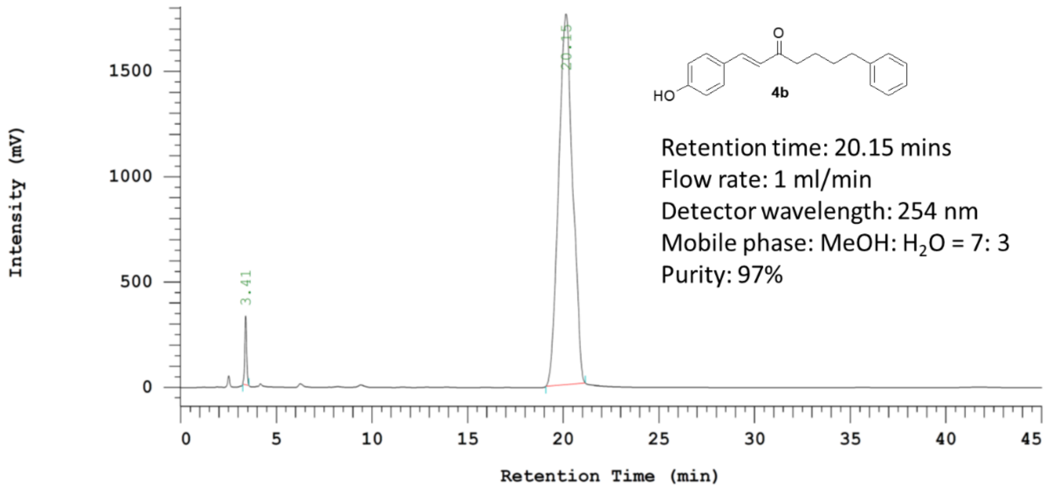

Supplementary Figure S63. HPLC chromatogram of compound **4b**

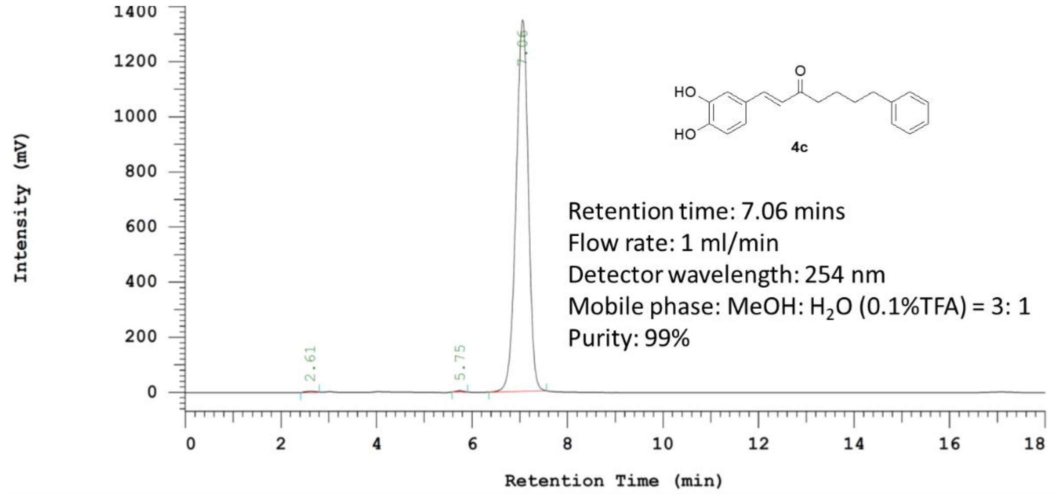

Supplementary Figure S64. HPLC chromatogram of compound **4c**

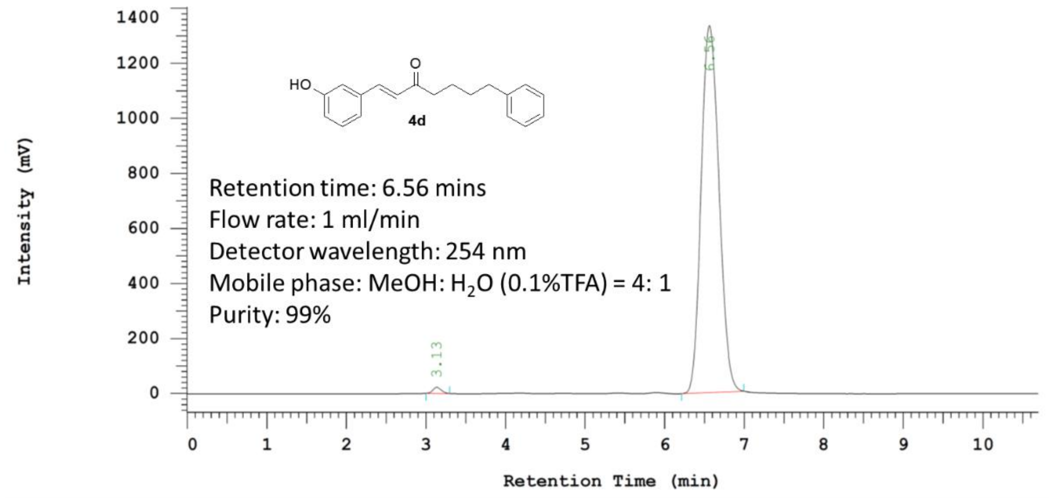

Supplementary Figure S65. HPLC chromatogram of compound **4d**

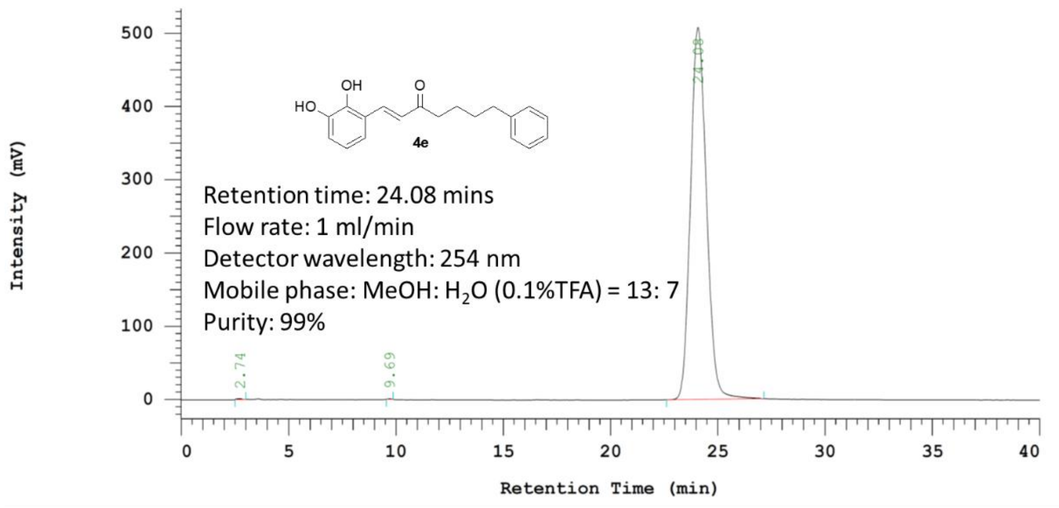

Supplementary Figure S66. HPLC chromatogram of compound **4e**

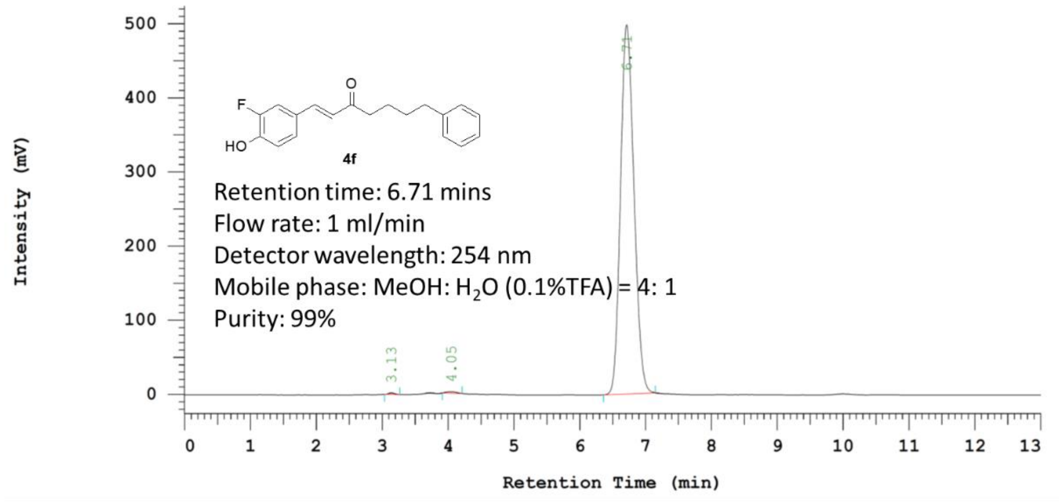

Supplementary Figure S67. HPLC chromatogram of compound **4f**

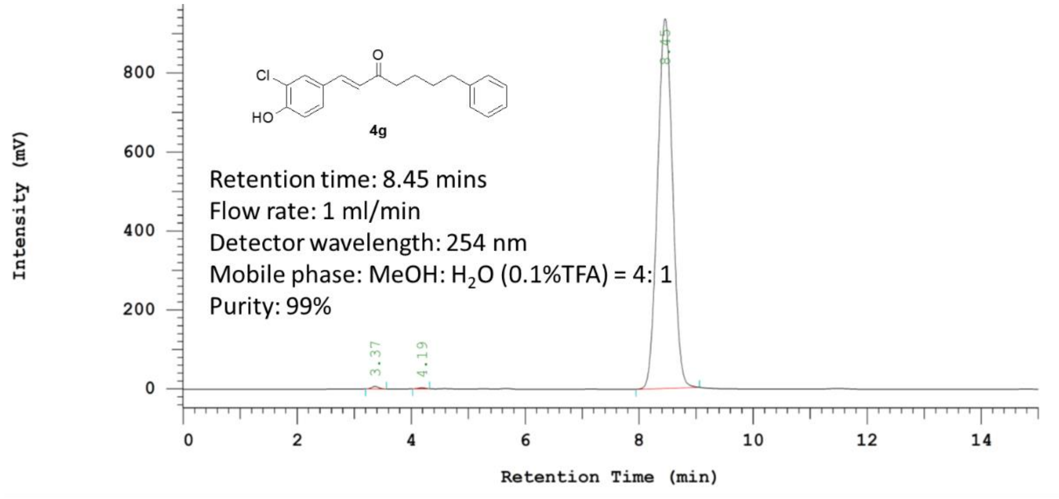

Supplementary Figure S68. HPLC chromatogram of compound **4g**

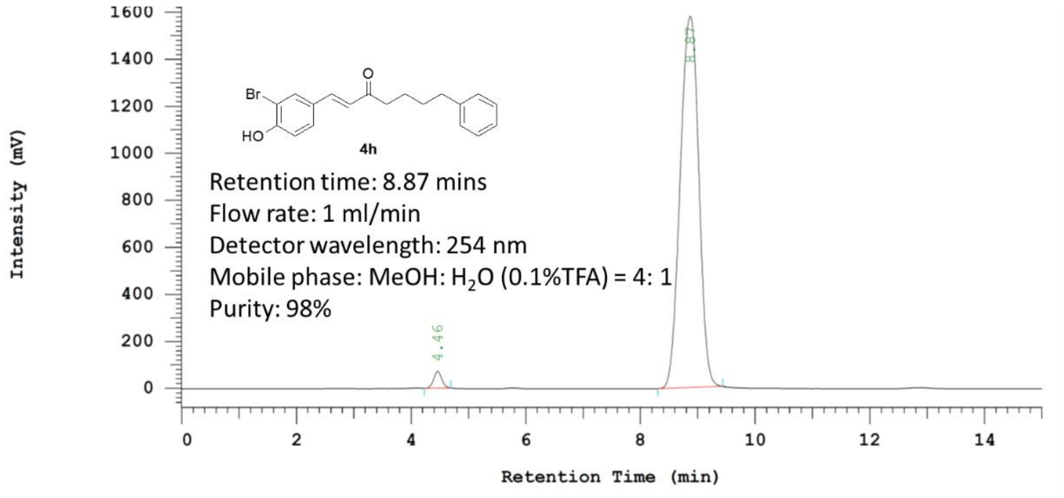

Supplementary Figure S69. HPLC chromatogram of compound **4h**

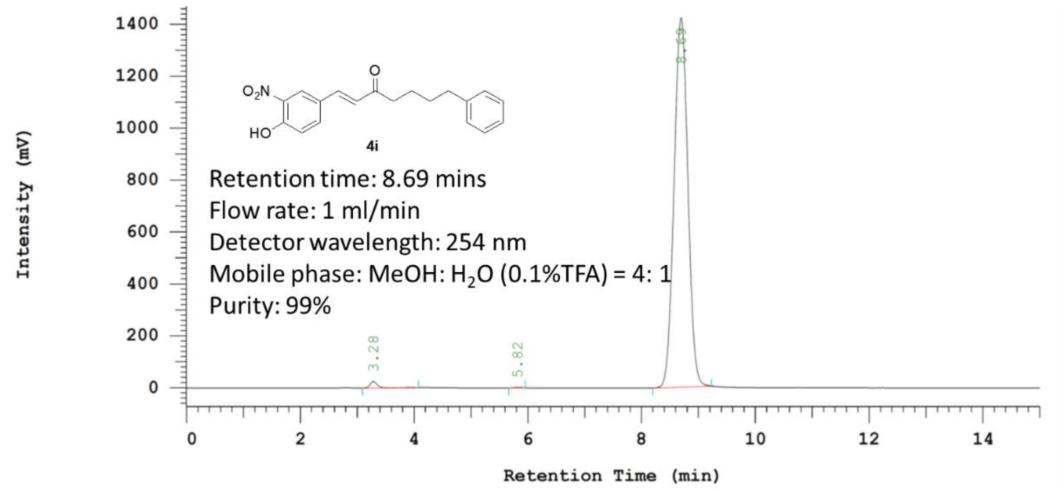

Supplementary Figure S70. HPLC chromatogram of compound **4i**

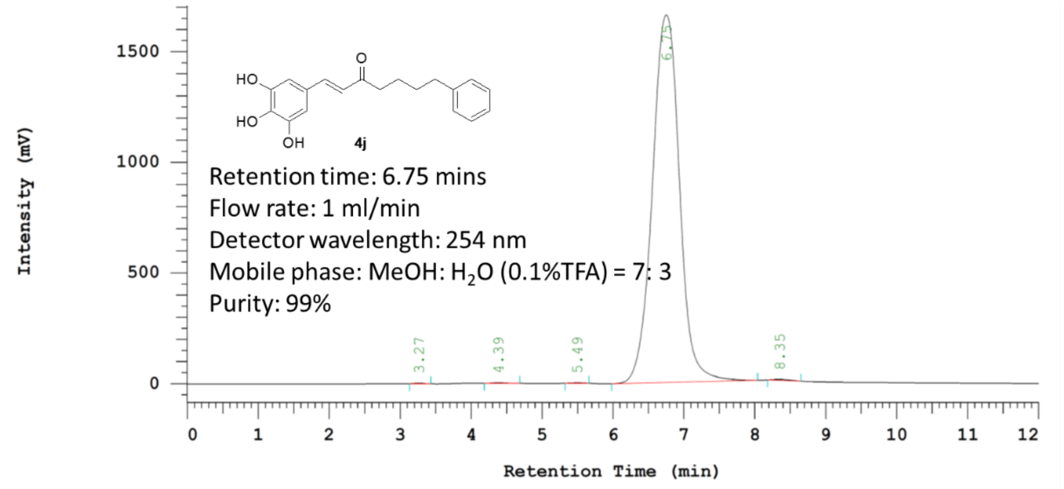

Supplementary Figure S71. HPLC chromatogram of compound **4j**

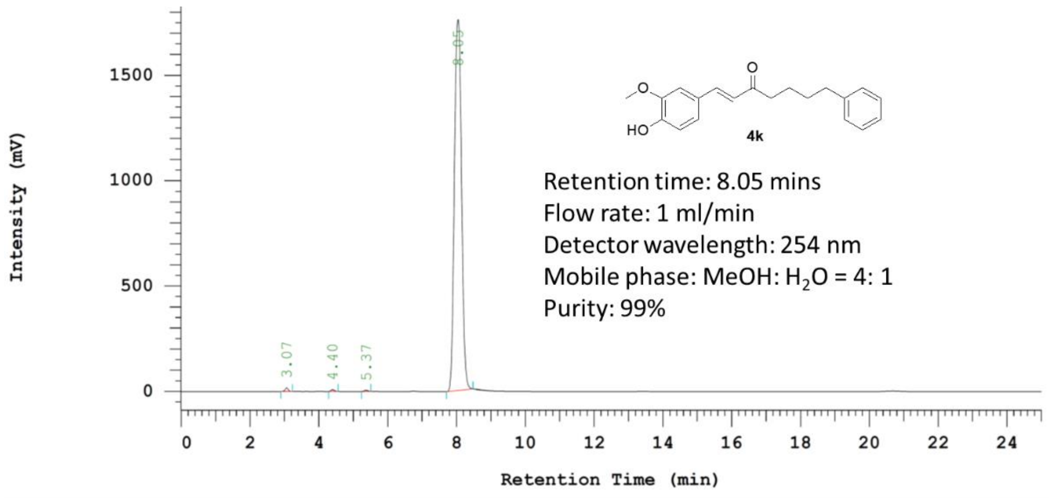

Supplementary Figure S72. HPLC chromatogram of compound **4k**

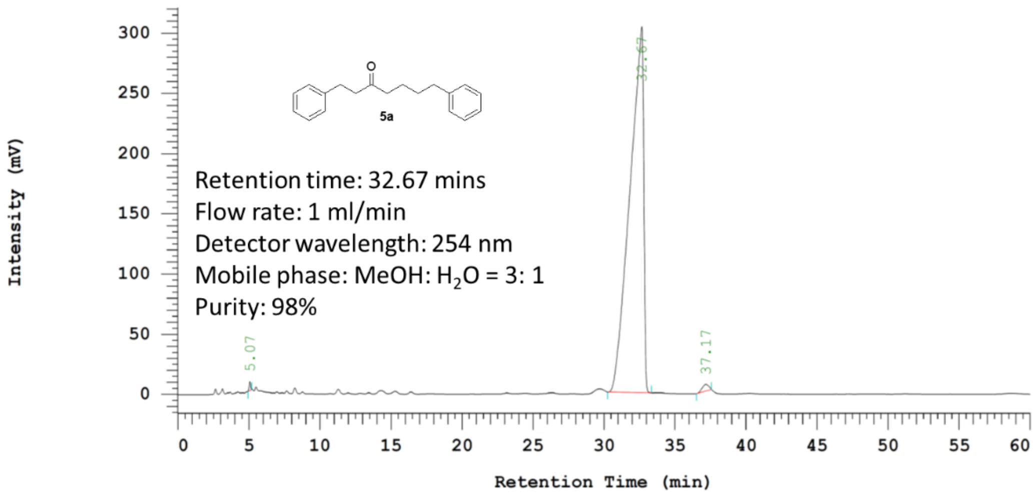

Supplementary Figure S73. HPLC chromatogram of compound **5a**

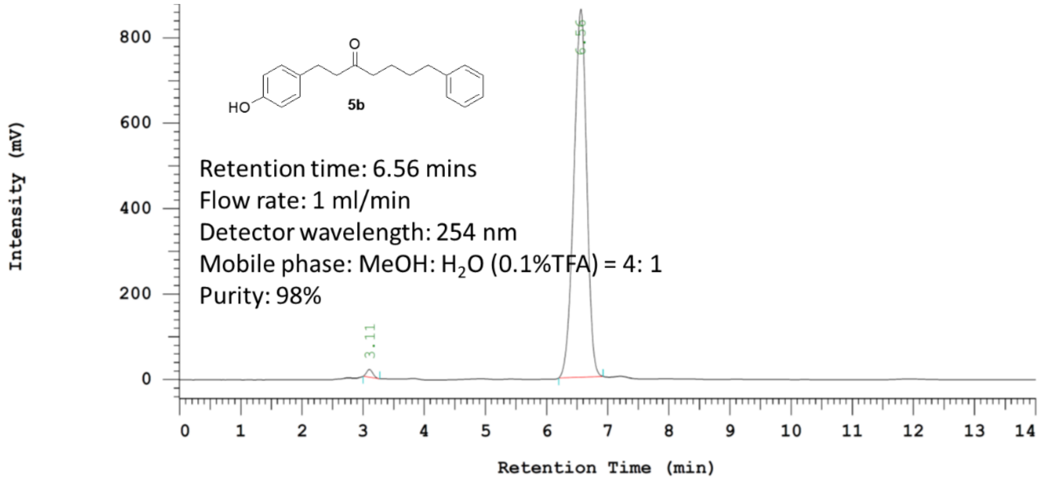

Supplementary Figure S74. HPLC chromatogram of compound **5b**

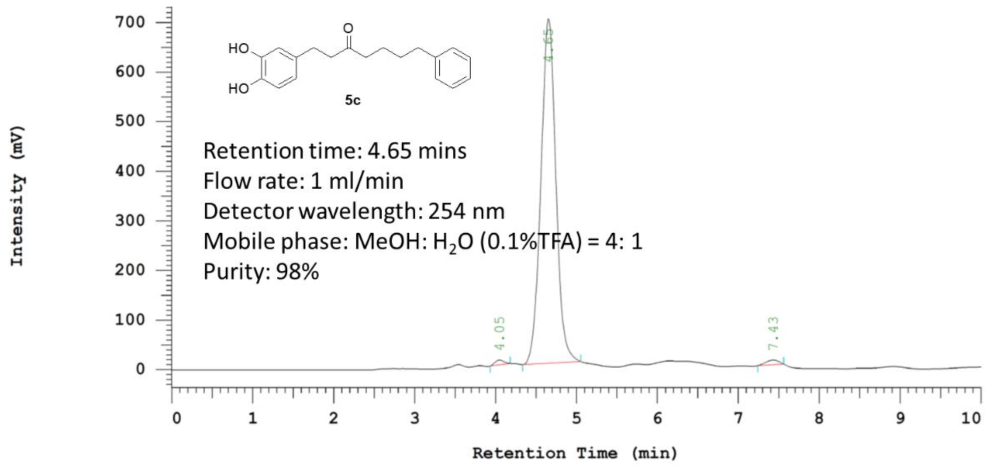

Supplementary Figure S75. HPLC chromatogram of compound **5c**

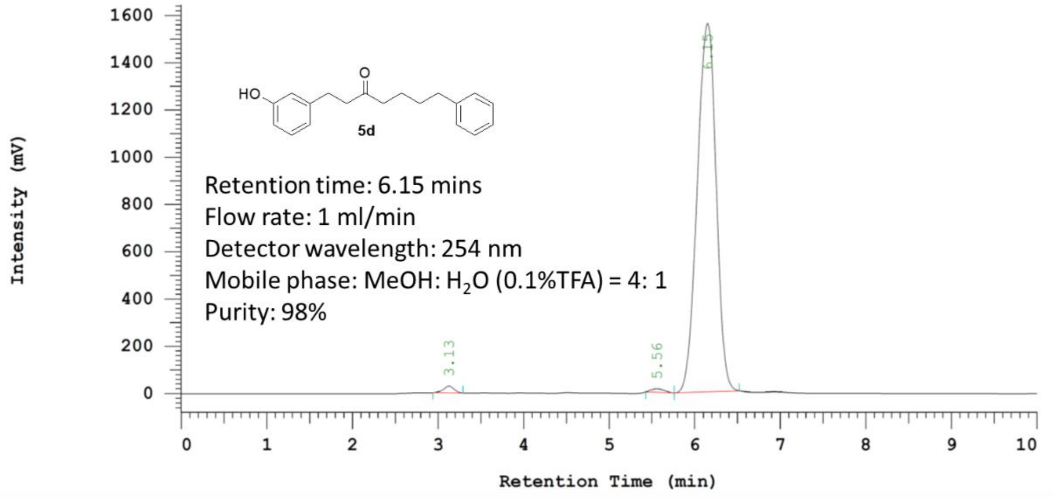

Supplementary Figure S76. HPLC chromatogram of compound **5d**

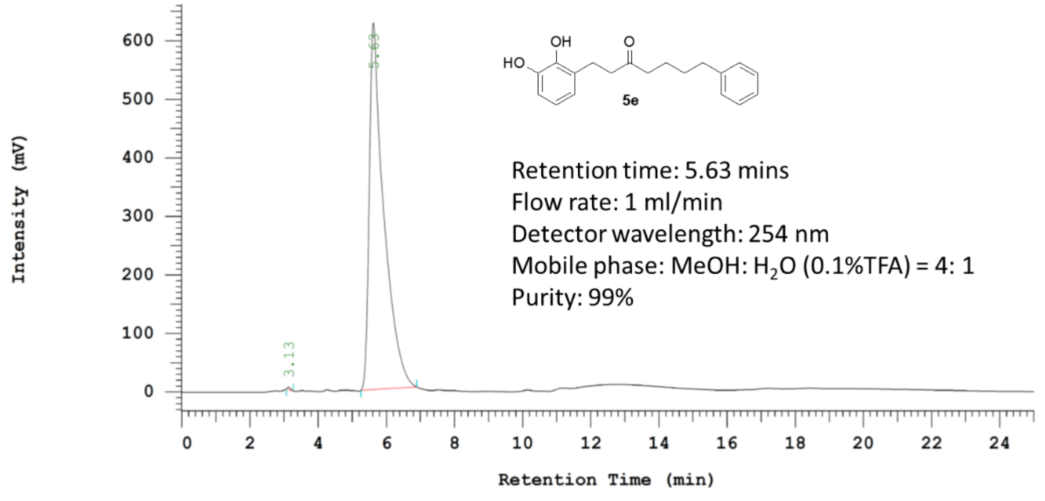

Supplementary Figure S77. HPLC chromatogram of compound **5e**

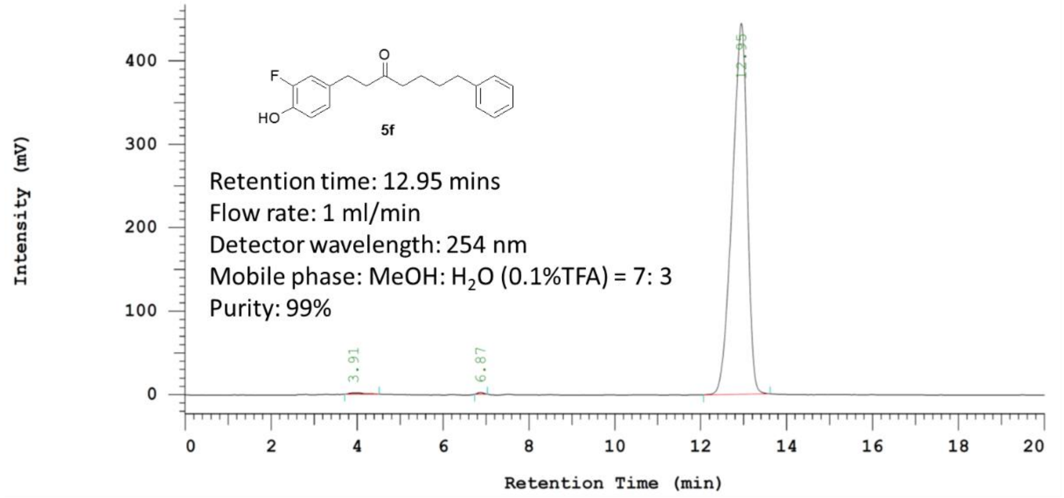

Supplementary Figure S78. HPLC chromatogram of compound **5f**

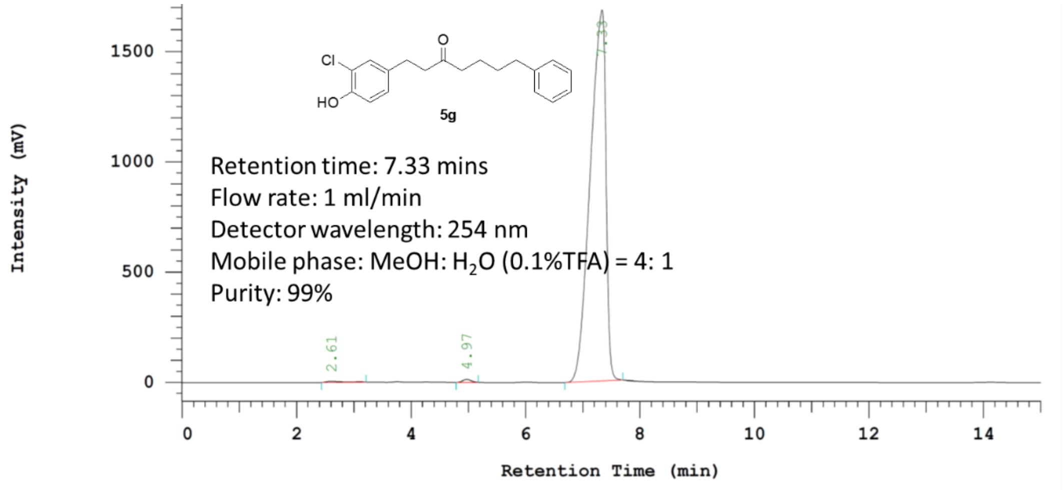

Supplementary Figure S79. HPLC chromatogram of compound **5g**

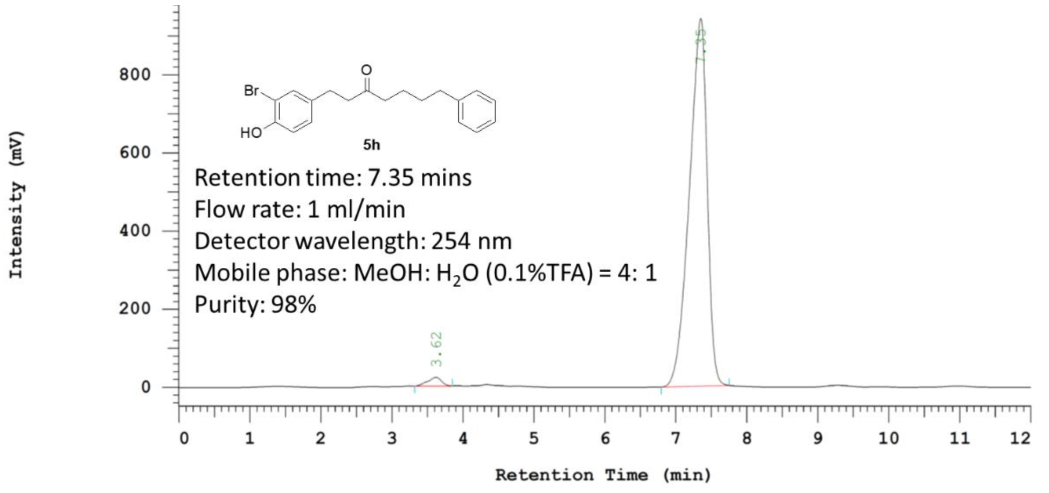

Supplementary Figure S80. HPLC chromatogram of compound **5h**

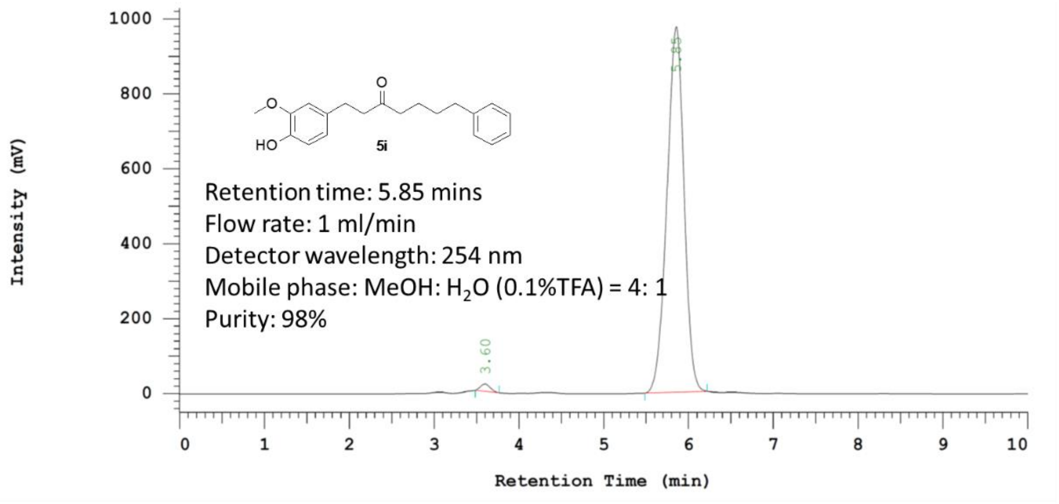

Supplementary Figure S81. HPLC chromatogram of compound **5i**

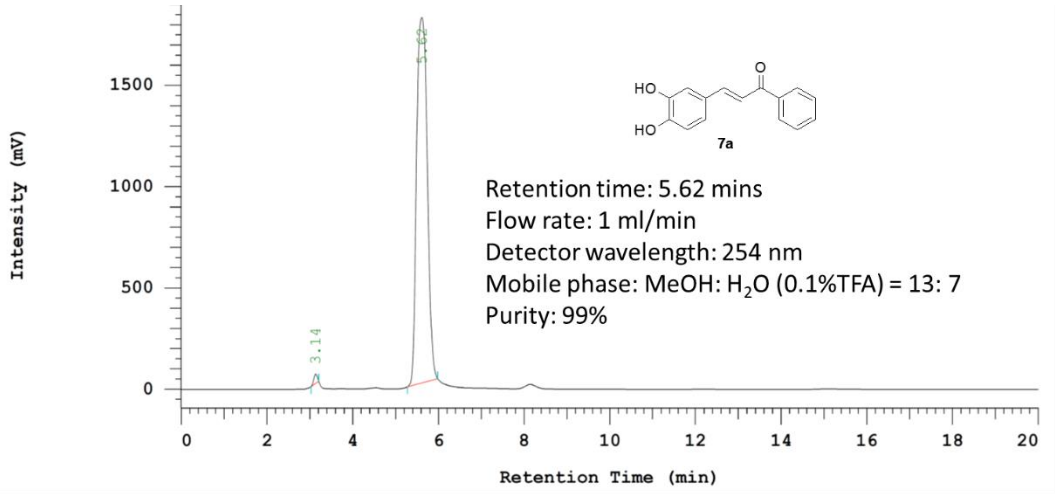

Supplementary Figure S82. HPLC chromatogram of compound **7a**

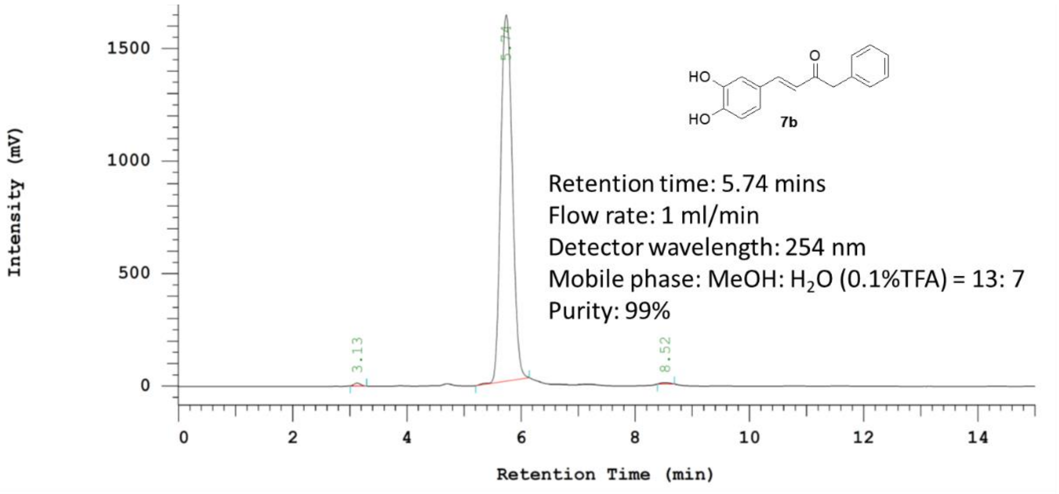

Supplementary Figure S83. HPLC chromatogram of compound **7b**

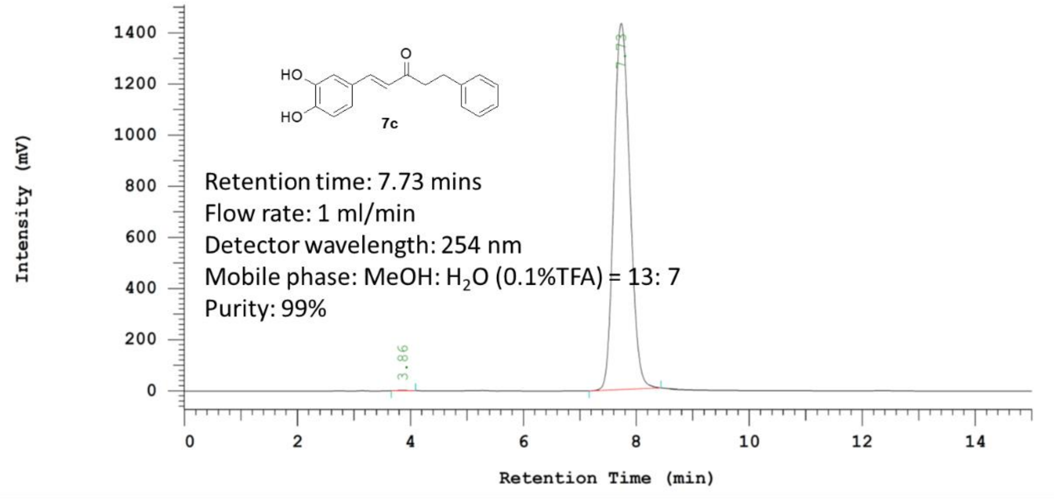

Supplementary Figure S84. HPLC chromatogram of compound **7c**

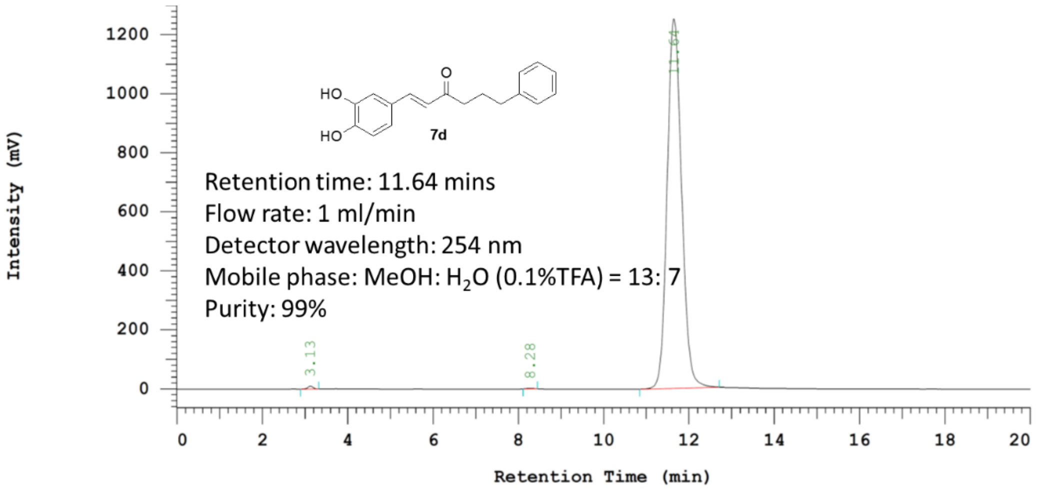

Supplementary Figure S85. HPLC chromatogram of compound **7d**

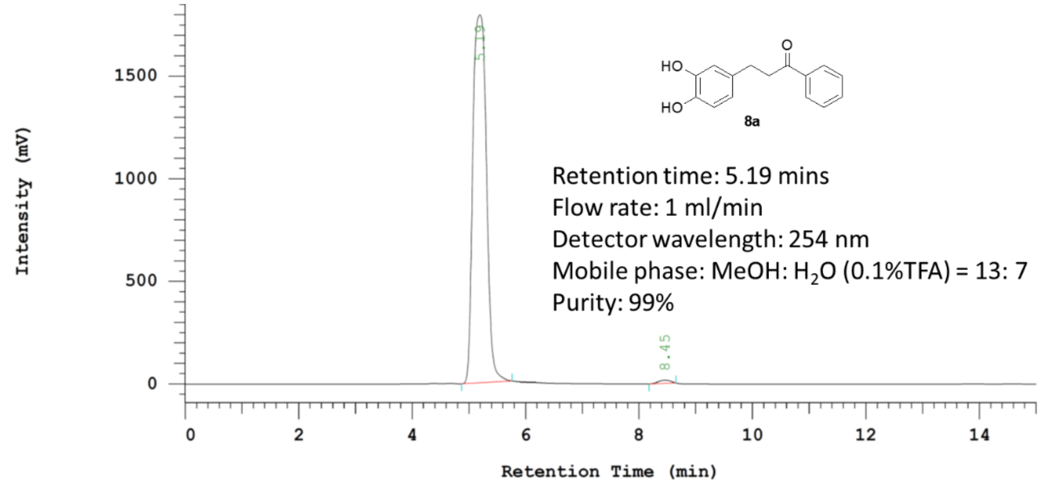

Supplementary Figure S86. HPLC chromatogram of compound **8a**

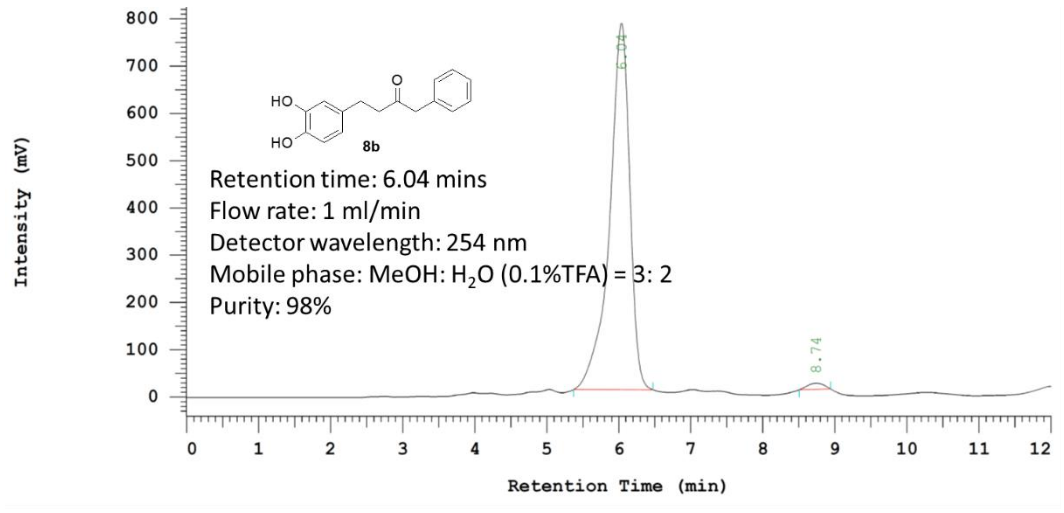

Supplementary Figure S87. HPLC chromatogram of compound **8b**

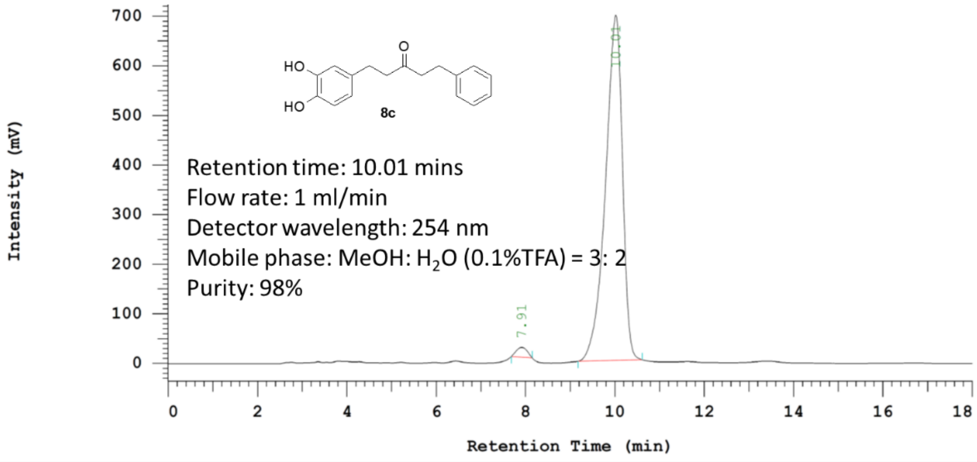

Supplementary Figure S88. HPLC chromatogram of compound **8c**

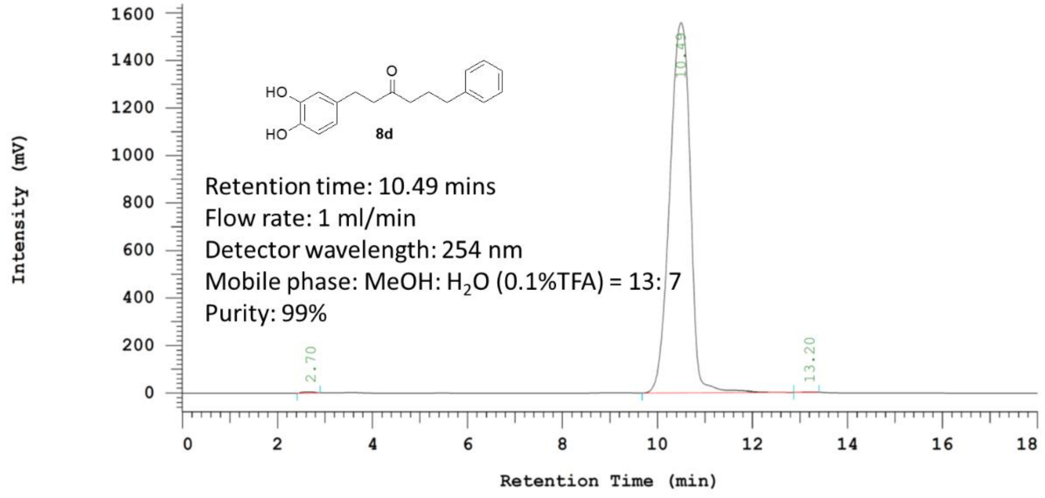

Supplementary Figure S89. HPLC chromatogram of compound **8d**

4 Chemical structures of synthetic compounds

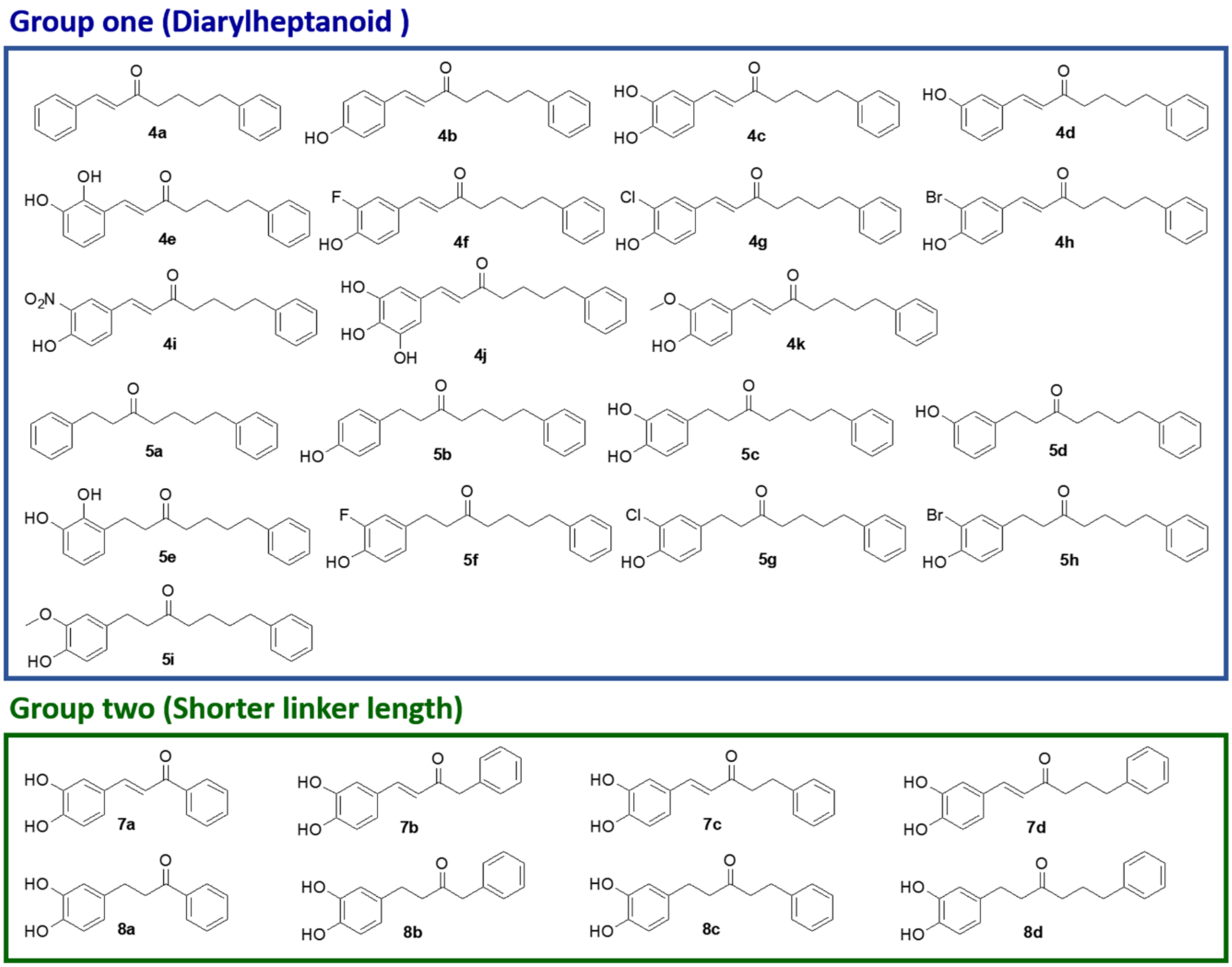

Supplementary Figure S90. Chemical structures of synthetic compounds.

5 Maximum common substructure (MCSS) and Root-Mean-Square Deviation (RMSD) of compounds

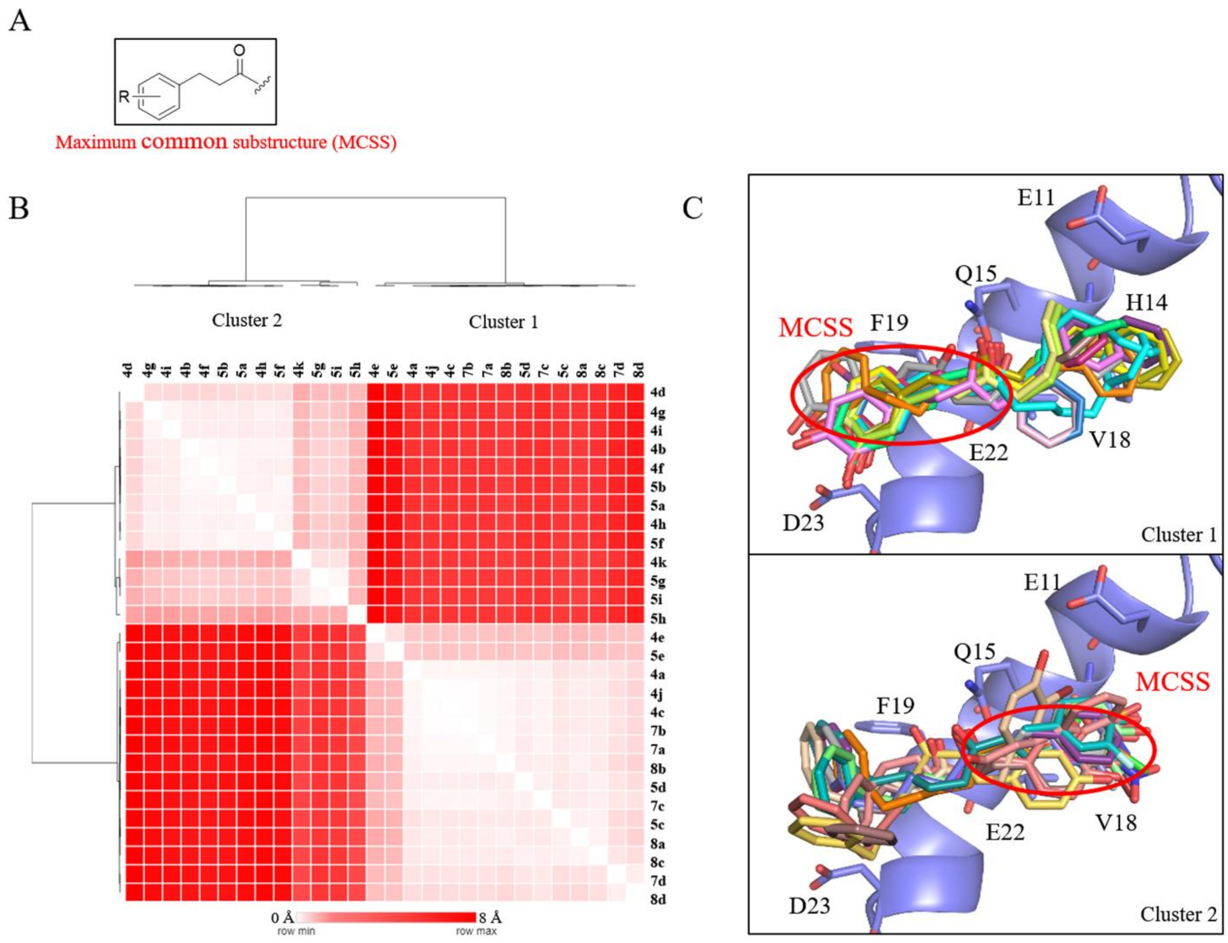

Supplementary Figure S91. Maximum common substructure (MCSS) of compounds and Root-Mean-Square Deviation (RMSD) of compound poses. (A) MCSS of compounds **4a-4k**, **5a-5i**, **7a-7d**, **8a-8d**. (B) The RMSD matrix of compound poses docked in A$\beta_{1\text{-}42}$ are colored white (0 Å) to red (8 Å) and classified in two clusters. The RMSD values between 2 compound poses is calculated by MCSS. (C) MCSS of two clusters of compound docking poses in A$\beta_{1\text{-}42}$ 3D structure. The red circle highlights two binding areas of the compounds in A$\beta_{1\text{-}42}$.

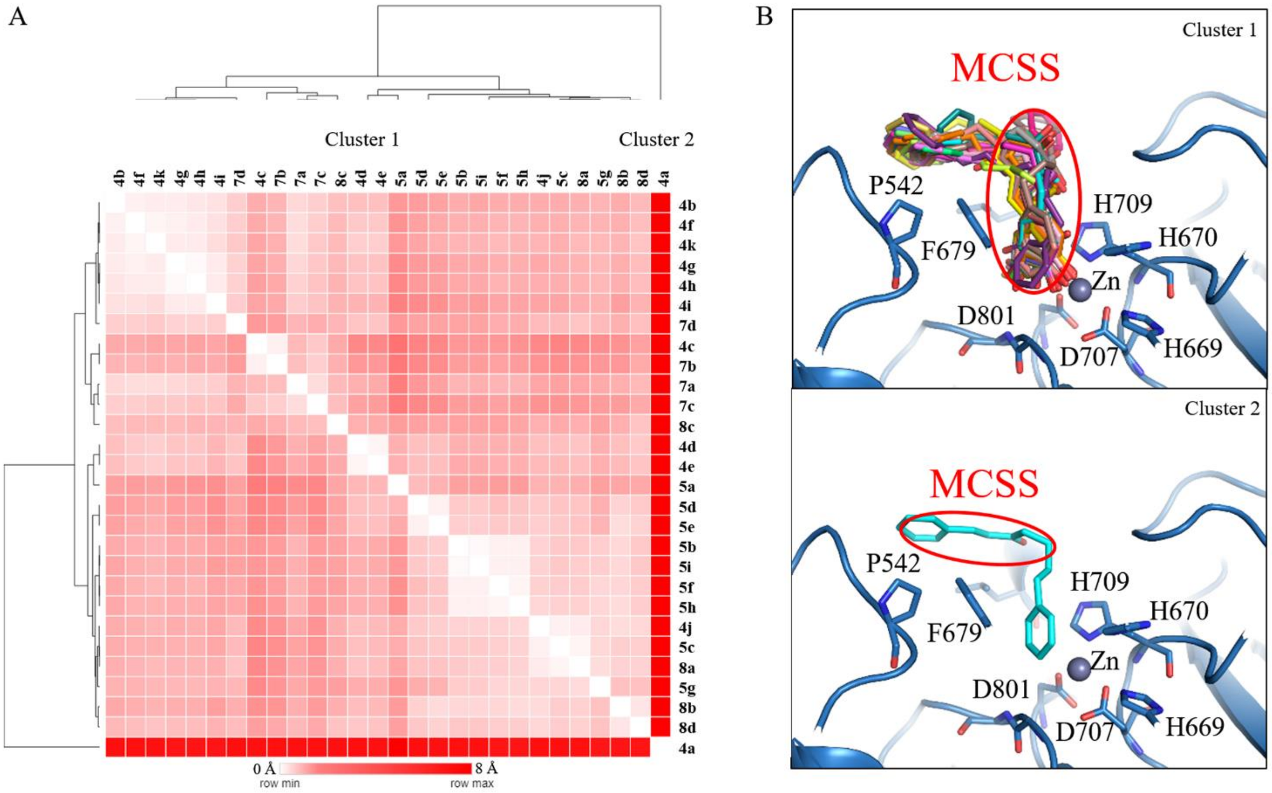

Supplementary Figure S92. Root-Mean-Square Deviation (RMSD) of compound poses in HDAC7. (A) The RMSD matrix of compound poses docked in HDAC7 are colored white (0 Å) to red (8 Å) and classified in two clusters. The RMSD values between 2 compound poses is calculated by MCSS. (B) MCSS of the two clusters of compound docking poses in HDAC7 3D structure. The red circle highlights the compound binding in the hydrophobic tunnel (top) and the cap region (bottom).

6 Cytotoxicity effect and neuroprotective effect on SH-SY5Y cells by trypan blue exclusion test

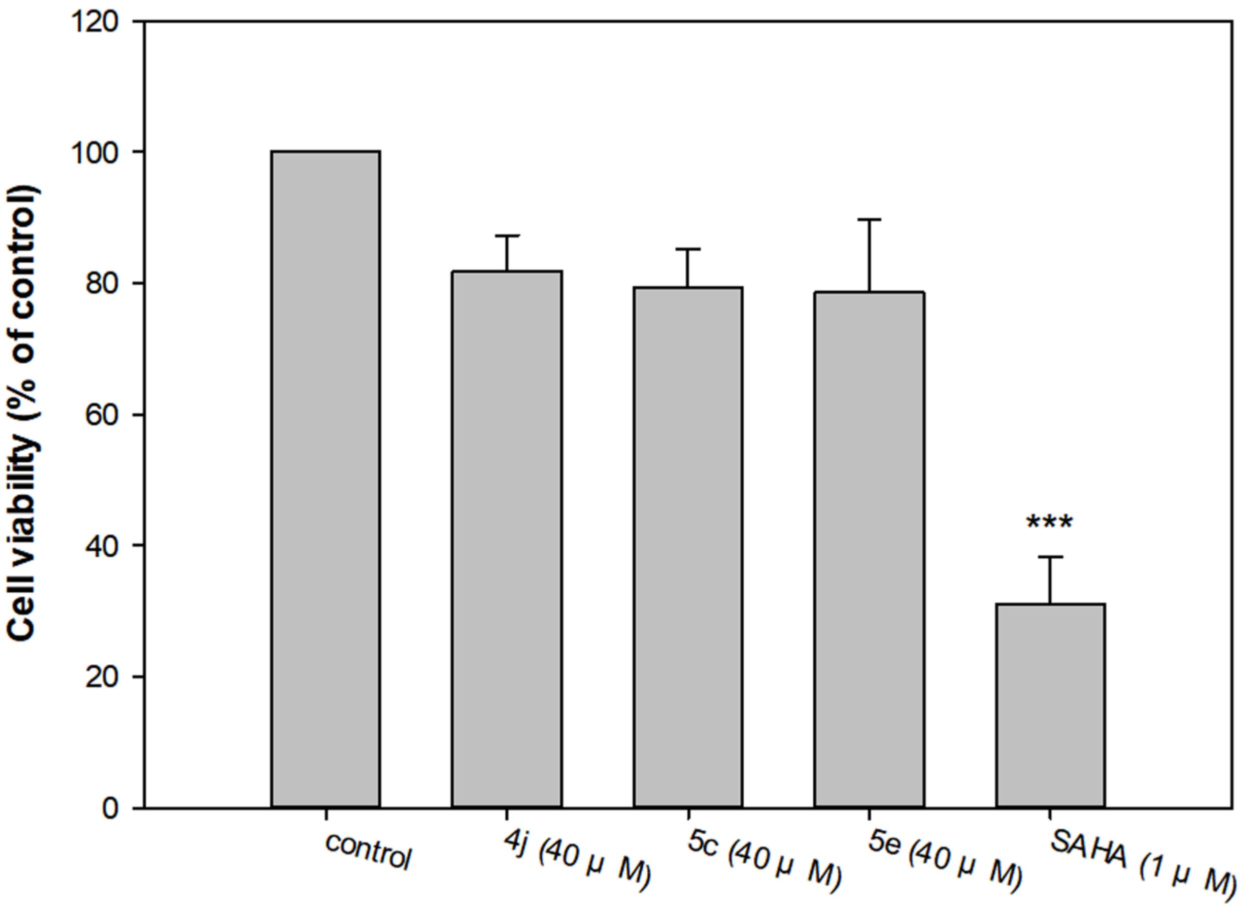

Supplementary Figure S93. Determination of the viability of compounds **4j**, **5c**, **5e** and SAHA of human neuroblastoma SH-SY5Y cells by trypan blue exclusion test. All data were expressed as mean ± SD of three experiments and each consisted of six replicates. *$p < 0.05$, **$p < 0.01$, ***$p < 0.001$ vs control. Statistical analysis was performed using one-way ANOVA followed by Bonferroni test.

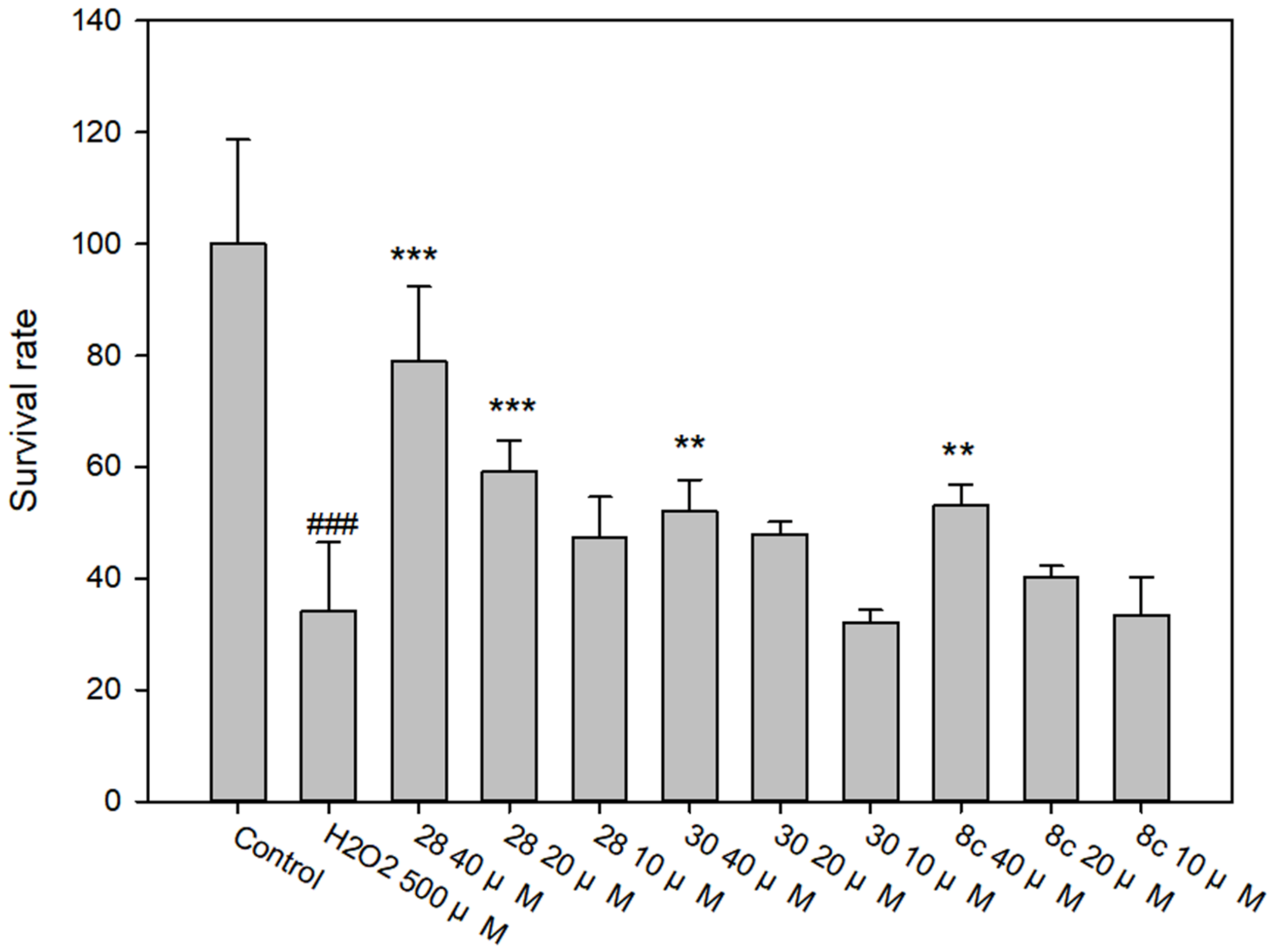

Supplementary Figure S94. Determination of the neuroprotective effects of compounds **4j**, **5c** and **5e** by trypan blue exclusion test. The compounds are tested at the concentration of 10, 20 and 40 μM on cell injury induced by H_2O_2 (500 μM) in human neuroblastoma SH-SY5Y cells. All data were expressed as mean ± SD of two experiments and each consisted of quadruplicates. $^{\#}p < 0.05$, $^{\#\#}p < 0.01$, $^{\#\#\#}p < 0.001$ vs control; $^{*}p < 0.05$, $^{**}p < 0.01$, $^{***}p < 0.001$ vs H_2O_2 alone. Statistical analysis was performed using one way ANOVA followed by Bonferroni test.

7 Total energy of compounds in A$\beta_{1\text{-}42}$ and HDAC7

Supplementary Table S1. Total energy of compounds **4a-k**, **5a-i**, **7a-d** and **8a-d** in A$\beta_{1\text{-}42}$

Compound	Total energy of protein-compound complex (ΔE, Kcal/mol)
**4a**	-1,457.92
**4b**	-1,468.04
**4c**	-1,493.03
**4d**	-1,452.67
**4e**	-1,478.33
**4f**	-1,467.62
**4g**	-1,467.18
**4h**	-1,475.26
**4i**	-1,454.85
**4j**	-1,486.42
**4k**	-1,442.18
**5a**	-1,455.49
**5b**	-1,470.53
**5c**	-1,478.29
**5d**	-1,476.85
**5e**	-1,470.98
**5f**	-1,470.34
**5g**	-1,450.90
**5h**	-1,457.12
**5i**	-1,458.74
**7a**	-1,488.09
**7b**	-1,488.54
**7c**	-1,489.96
**7d**	-1,497.85
**8a**	-1,485.43
**8b**	-1,488.94
**8c**	-1,492.30
**8d**	-1,490.31

Supplementary Table S2. Total energy of compounds **4a-k**, **5a-i**, **7a-d** and **8a-d** in HDAC7

Compound	Total energy of protein-compound complex (ΔE, Kcal/mol)
**4a**	-16,080.90
**4b**	-16,121.70
**4c**	-16,124.20
**4d**	-16,090.80
**4e**	-16,104.60
**4f**	-16,085.20
**4g**	-16,080.60
**4h**	-16,090.60
**4i**	-16,050.10
**4j**	-16,106.10
**4k**	-16,101.90
**5a**	-16,093.50
**5b**	-16,149.50
**5c**	-16,133.70
**5d**	-16,145.20
**5e**	-16,150.80
**5f**	-16,103.20
**5g**	-16,118.90
**5h**	-16,129.80
**5i**	-16,150.50
**7a**	-16,083.50
**7b**	-16,127.20
**7c**	-16,122.70
**7d**	-16,105
**8a**	-16,071.40
**8b**	-16,148.70
**8c**	-16,133.40
**8d**	-16,143.90

Supplementary Table S3. ADMET prediction of compounds **4j**, **5c** and **5e**

Compound	BBB penetration [a]	Human intestinal absorption [b]	Aqueous solubility [c]
**4j**	2 (Medium)	0 (Good)	3 (Good)
**5c**	1 (High)	0 (Good)	3 (Good)
**5e**	1 (High)	0 (Good)	3 (Good)

[a] The prediction of blood-brain permeation after oral administration.
[b] Intestinal absorption is defined as percentage absorbed after oral administration.
[c] The aqueous solubility of a compound in water at 25 ℃.

8 Reference:

Degenhardt, K., Sundararajan, R., Lindsten, T., Thompson, C., and White, E. (2002). Bax and Bak independently promote cytochrome C release from mitochondria. *J. Biol. Chem.* 277**,** 14127-14134.

Pan, S., Cai, H., Gu, L., and Cao, S. (2017). Cleistanthin A inhibits the invasion and metastasis of human melanoma cells by inhibiting the expression of matrix metallopeptidase-2 and -9. *Oncol. Lett.* 14**,** 6217-6223.

Zanoni, M., Piccinini, F., Arienti, C., Zamagni, A., Santi, S., Polico, R., Bevilacqua, A., and Tesei, A. (2016). 3D tumor spheroid models for in vitro therapeutic screening: a systematic approach to enhance the biological relevance of data obtained. *Sci. Rep.* 6**,** 19103.
